# Functional decline in facial expression generation in older women: A cross-sectional study using three-dimensional morphometry

**DOI:** 10.1371/journal.pone.0219451

**Published:** 2019-07-10

**Authors:** Chihiro Tanikawa, Sadaki Takata, Ruriko Takano, Haruna Yamanami, Zere Edlira, Kenji Takada

**Affiliations:** 1 Department of Orthodontics and Dentofacial Orthopedics, Graduate School of Dentistry, Osaka University, Suita, Osaka, Japan; 2 Center for Advanced Medical Engineering and Informatics, Osaka University, Suita, Osaka, Japan; 3 Department of Fashion & Beauty Sciences, Osaka Shoin Women’s University, Higashi-Osaka, Osaka, Japan; 4 Corporate Culture Department, Shiseido Co., ltd., Tokyo, Japan; 5 Shiseido Global Innovation Center, Shiseido Co., ltd., Yokohama, Kanagawa, Japan; 6 Faculty of Dentistry, National University of Singapore, Singapore, Republic of Singapore; Institute of Automation, Chinese Academy of Sciences (CASIA), CHINA

## Abstract

Elderly people show a decline in the ability to decode facial expressions, but also experience age-related facial structure changes that may render their facial expressions harder to decode. However, to date there is no empirical evidence to support the latter mechanism. The objective of this study was to assess the effects of age on facial morphology at rest and during smiling, in younger (n = 100; age range, 18–32 years) and older (n = 30; age range, 55–65 years) Japanese women. Three-dimensional images of each subject’s face at rest and during smiling were obtained and wire mesh fitting was performed on each image to quantify the facial surface morphology. The mean node coordinates in each facial posture were compared between the groups using t-tests. Further, the node coordinates of the fitted mesh were entered into a principal component analysis (PCA) and a multifactor analysis of variance (MANOVA) to examine the direct interactions of aging and facial postures on the 3D facial morphology. The results indicated that there were significant age-related 3D facial changes in facial expression generation and the transition from resting to smiling produced a smaller amount of soft tissue movement in the older group than in the younger group. Further, 185 surface configuration variables were extracted and the variables were used to create four discriminant functions: the age-group discrimination for each facial expression, and the facial expression discrimination for each age group. For facial expression discrimination, the older group showed 80% accuracy with 2 of 66 significant variables, whereas the younger group showed 99% accuracy with 15 of 144 significant variables. These results indicate that in both facial expressions, the facial morphology was distinctly different in the younger and older subjects, and that in the older group, the facial morphology during smiling could not be as easily discriminated from the morphology at rest as in the younger group. These results may help to explain one aspect of the communication dysfunction observed in older people.

## Introduction

Facial expressions play an important role in the communication of emotions and thoughts. It is no exaggeration to say that the face is an organ of communication. Aging is related to impairment of various motor and cognitive functions. A recent study that examined the perception of emotions found that facial expressions have reduced signal clarity when shown on older faces, especially for smiling [1]. This suggests that aging results in dysfunctional communication.

However, the mechanisms underlying age-related communication dysfunction are incompletely understood. Aging of the face affects facial configurations and their changes during facial expressions. A Moire 3D analysis system determined that facial sagging becomes progressively more noticeable with aging [2]. Three-dimensional analysis of labial morphology showed a significant effect of age on labial thickness and area [3]. A study comparing 3D faces of mothers and daughters found that the greatest atrophy associated with aging was observed in the upper lip, lateral canthi, labial commissures, and gonial angle [4]. When optical images were used to distinguish nasolabial lines, it was found that the lines were significantly increased age-dependently [5]. A study that investigated age effects on the relationship between teeth and facial soft tissue found that the perioral soft tissues dropped down in older subjects and the soft tissue descended along the entire labial arch [6]. Another study found that young people had a larger lip area and thickness than elderly people [3] [7]. A recent study showed that facial features at rest are more reliable aging biomarkers than blood profiles [8], in which eye slopes were identified as highly associated with age. These results show that aging affects the facial configuration at rest; however, there remain unanswered questions about the effects of aging on facial configurations during smiling.

In the present study, we focused on female subjects, because the functional decline in facial expression recognizability was considered more important for women under the forthcoming super-aging societies wherein women have a life expectancy at birth 4.7 years longer than that of men, averaged across countries [9]. Further, a meta-analysis showed that women smile more frequently than men do [10], and this suggests the existence of the interaction of sex effects on functional decline.

Recently, Tanikawa et al. [11] developed an objective method for evaluating the 3D soft tissue configuration of the face. Using that method, the present study aimed (1) to clarify whether the entire facial surface differs in form between older women and younger women; and (2) to clarify the mechanisms of age-related nonverbal communication dysfunction.

## Materials and methods

**Fig 1:**
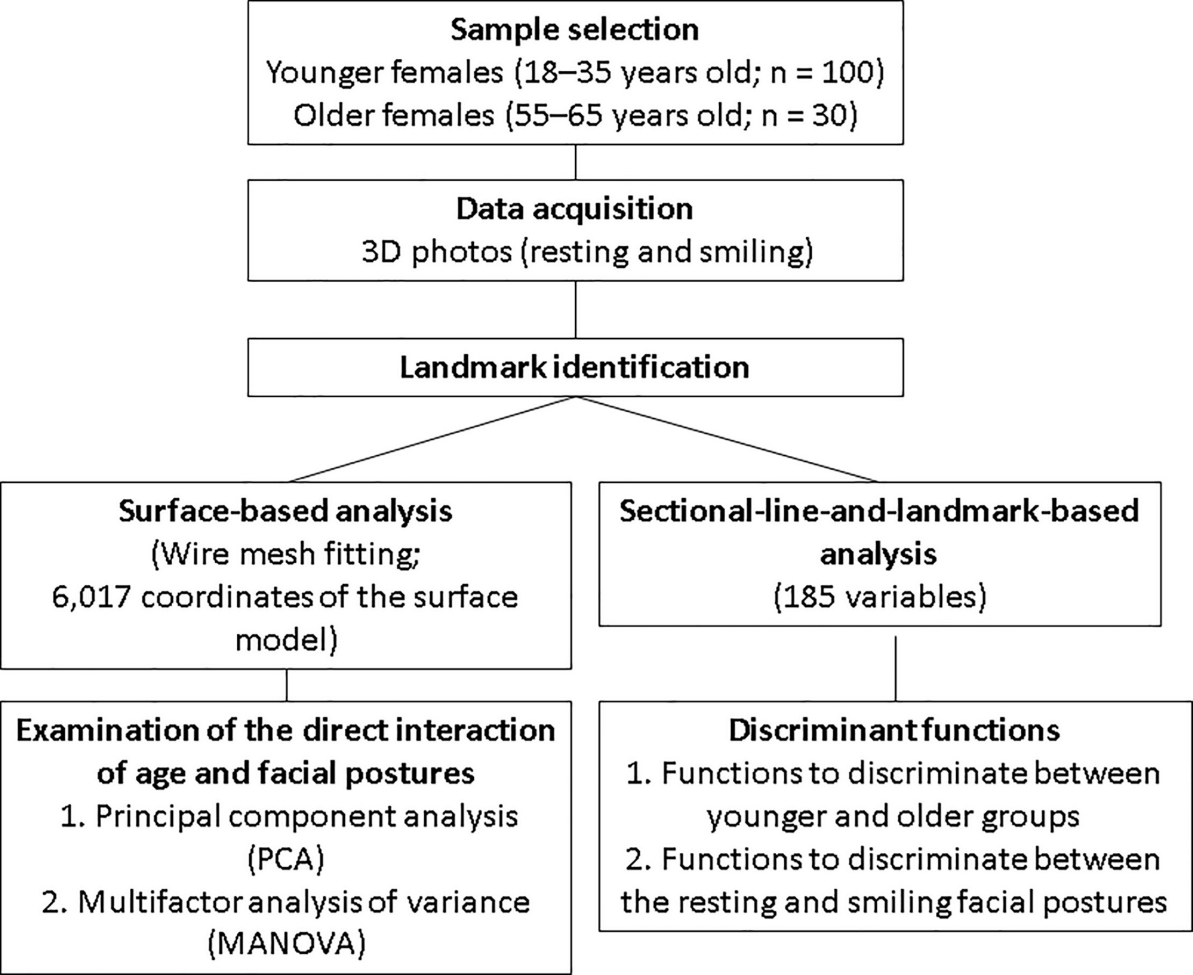
Overview of the data analysis from data acquisition to statistical analysis.

This was a cross-sectional cohort study, and Institutional Ethics Committee Approval was obtained (Osaka University, H20-E19-3). All procedures conducted conformed to the guidelines of the Declaration of Helsinki. Overview of the data analysis from data acquisition to statistical analysis is shown in Fig 1.

### Data acquisition and coordinate system

We included a total of 130 Japanese adult women consisting of two groups: the younger group (n = 100) was recruited from female students and faculty of a dental school (date range, Dec, 2009 –Jan, 2012); the older group (n = 30) was recruited by a private investigation agency (Osaka, Japan; date range, Dec, 2009 –Jan, 2010). The inclusion criteria were as follows: sex (female), age range (younger = 18–35, older = 55–65), no congenital facial deformities including cleft lip or palate, no facial paralysis, no noticeable scars or skin disease in the neck and dentofacial regions (or history thereof), no history of any psychiatric disorder, no subjectively or objectively discernible jaw dysfunction, a body mass index ranging from 18.50 to 24.99, an overbite ranging from 1.0 mm to 5.0 mm, an overjet ranging from 0.0 mm to 7.0 mm, and a straight soft-tissue facial profile [11].

An IRB-approved written informed consent form was distributed to and signed by all subjects.

### Data acquisition

The subjects were asked to sit on a fixed chair with a natural head position without head support, in front of a three-dimensional image-capturing device (3dMDcranial System, 3dMD, Atlanta, GA, USA). They were then asked to perform two tasks: (1) to form a resting facial expression, and (2) to form the peak of a posed smile. The accuracy of the facial expressions was described in previous studies [12, 13]. The subjects were instructed vocally for each task and asked to maintain the expressions for about 2 seconds. After two to three rehearsals, each expression was recorded once with the three-dimensional image-capturing device. Recordings of each type of expression were made with a resting interval of about 20 seconds between the expressions. The experimenter operated the system from a position out of the subject's view.

### Landmark identification

As described in detail previously [12, 13], each 3D facial image was displayed on a 17-in LCD monitor (1701FP, Dell, Inc., Round Rock, TX, USA), scaled down to 75% of its actual size. The positions of 10 single and 8 paired landmarks (glabella [Gla], nasion [N], exocanthion [Ex], endocanthion [En], palpebrale superius [Ps], palpebrale inferius [Pi], porion [Po], orbitale [Or], pronasale [Prn], alar curvature point [Ac], subnasale [Sn], labiale superius [Ls], stomion [Sto], cheilion [Ch], labiale inferius [Li], submentale [Sm], pogonion [Pog], gnathion [Gn]) [14] (S1 Fig and S1 Table) were identified by visual inspection of the image using a computer mouse cursor, and digitized using commercial software (Face Rugle, Medic Engineering Co., Kyoto, Japan). The process was repeated twice for each image, and the landmark coordinates from the two digitizations produced were averaged to yield the final landmark coordinates. A previous study [11] that investigated intra-observer reliability confirmed a mean absolute landmark difference of 0.32 mm (range, 0.07 mm to 0.52 mm) between the repeated measures. This result falls into the range considered reliable-to-highly-reliable.

### Coordinate system

A 3D coordinate system identical to that employed in our previous study (S1 Fig) [11] was used in the current study. Briefly, the sagittal plane (Y-Z plane) was defined by the exocanthions and endocanthions, and the axial plane (X-Z plane) was defined by the exocanthions, porion, and subnasale. The nasion was set as the origin.

### Analyses

We employed 2 types of analyses described in our previous study [11]: (1) surface-based analysis with creation of average faces; and (2) sectional-line-and-landmark-based analysis with discriminant analysis. The former was conducted to examine the morphological characteristics of the faces at rest and during smiling; the latter was conducted to create discriminant functions, that is, age group discrimination for each facial expression, and facial expression discrimination for each age group. Details are described below.

### Surface-based analysis

#### Averaged faces and accentuated average faces

For each participant and each facial expression, a fitting of high-resolution template meshes based on the assignment of landmarks to each 3D facial image was performed using software (HBM-Rugle, Medic Engineering Co., Kyoto, Japan). This method automatically generated homogeneous models consisting of 6,017 points (i.e., fitted mesh or semi-landmark nodes) on a wire mesh, with landmark anchors (Ex, En, Ps, Pi, Prn, Ac, Sn, Ls, Sto, Ch, Li, Sm, and Pog). As described in detail previously [15], this technique permits the extraction of relevant surface anatomy from facial data while removing or smoothing out non-relevant data, yielding high-resolution 3D surface data that provides enough detail to facilitate a quantitative assessment while maintaining small file sizes that are easily manipulatable and portable to a range of visualization technologies. The arithmetic means of the coordinate values and the color values of each corresponding point on the wire mesh were computed and used to generate 3D-averaged facial images for each subject group.

To quantitatively facilitate intuitive understanding of aging of the facial forms, accentuated averaged faces, (*AccA*(*Y*)) and (*AccA*(O)), were calculated for the younger and older groups, respectively, to highlight the differences between the two subject groups, where

(AccA(Y))=(A(Y))+w((A(Y))−(A(O)))(w=2)

(AccA(E))=(A(E))+w((A(O))−(A(Y)))(w=2),

where (*A*(*Y*)) and (*A*(O)) are the arithmetic means of the coordinate values for the younger group and older group, respectively, and *w* is an arbitrary weight value.

#### Comparison between the averaged faces of the sample groups

The arithmetic means of the coordinate values of each corresponding point on the wire mesh were statistically analyzed for determining whether there were significant differences between the younger and older groups using a two-sample t-test. A significance probability map [11, 15] of the X, Y, and Z value was generated to visualize the significant differences.

#### Examination of the interaction of age and facial postures

To examine the direct interaction of age and facial postures, we first performed a principal component analysis (PCA) for the 6,017 coordinates of the aforementioned surface model. The significant PCs were determined by a scree plot analysis. Significant PCs were entered into a multifactor analysis of variance (MANOVA) to test the significance of age and facial postures. After MANOVA, a dendrogram was computed by applying the single linkage method to the matrix of Mahalanobis distances between the subgroup means.

### Sectional-line-and-landmark-based analysis

#### Measurements

Sectional-line-and-landmark-based analysis was conducted following the technique used in our previous study [11]. Briefly, we extracted 5 categories of curving lines from the 3D images: inter-landmark contours, sagittal sections, axial sections, facial outlines, and supraorbital ridge outlines (S2 Table). The curving lines were used to extract 142 measurements, as described in previous study [11]. In addition, 28 inter-landmark distances and 15 ratios that had been reported in previous studies [14, 16–17] were determined and employed. Therefore, 185 variables were employed in total. For definition of the variables, please see S1 Appendix, S1–S6 Figs.

#### Statistics

A t-test was performed to determine whether the mean of each variable differed significantly between the older group and the younger group. The variables for which P < 0.01 were entered as predictor variables into a stepwise discriminant function analysis for age group discrimination. Paired t-tests were performed for each group to determine whether there were differences in each variable between the resting and smiling postures. The variables showing P < 0.01 were also used to determine a discriminant function for facial expression discrimination via stepwise processes. Statistical programs included with R (http://www.r-project.org/) were used. The statistical technique involved the creation of a linear equation that could be used to predict to which group a case belonged. The form of the equation or function was:

$$\mathrm{Discriminant}\;\mathrm{function}=v_{1}x_{1}+v_{2}x_{2}+\cdots +v_{n}x_{n}+a$$

where *v*_i_ is the discriminant coefficient (weight for that variable), *a* is a constant, and *n* is the number of predictor variables. Standardized discriminant coefficients were also calculated. In the stepwise discriminant function analysis, the criterion for adding or removing a variable was the significance level of the F-value (P < 0.01) [18].

## Results

### Qualitative analysis of the averaged faces

**Fig 2:**
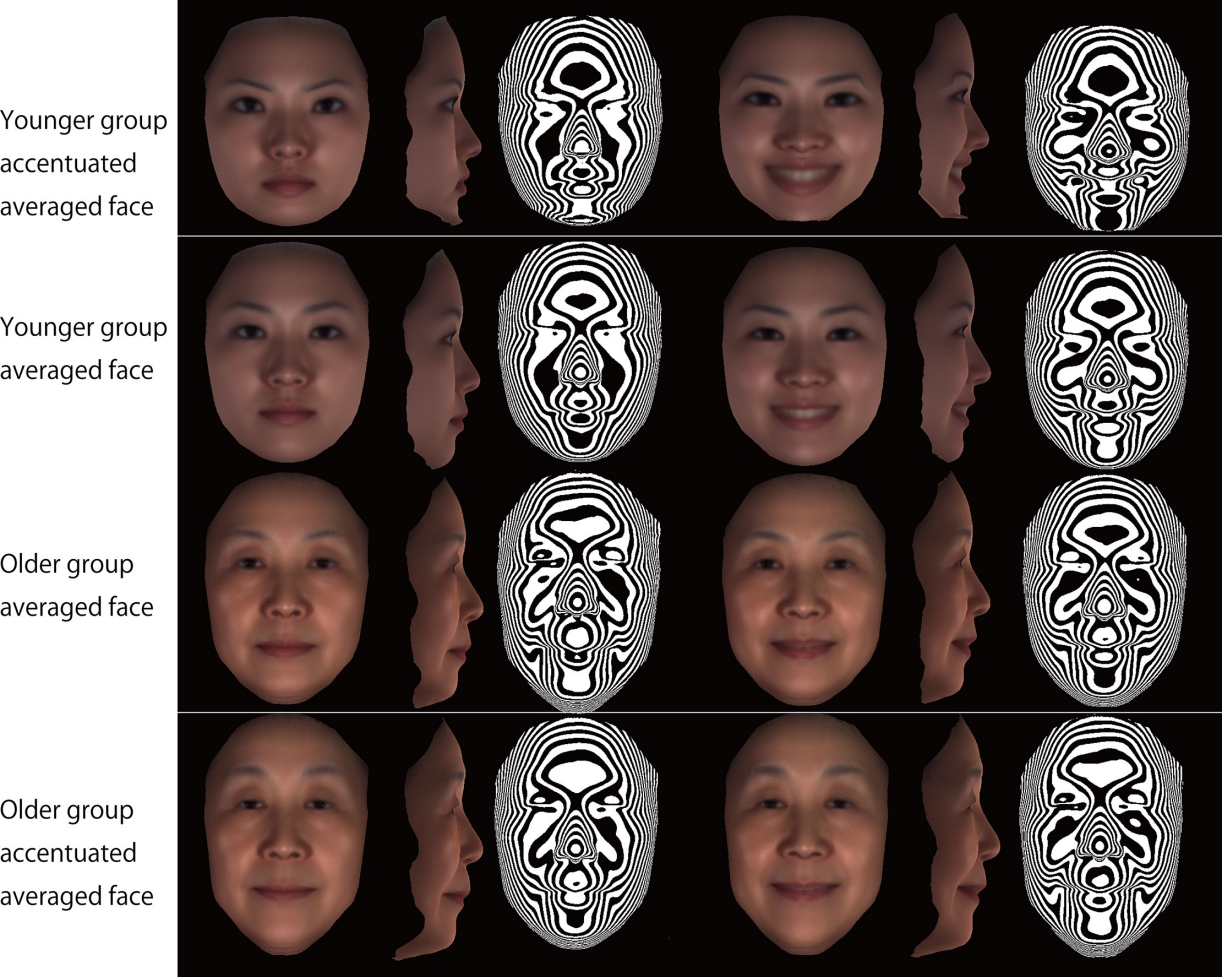
Computer-generated model faces to facilitate intuitive understanding of aging effects by illustrating site-specific accentuated facial topography. Left, frontal view of rest posture; second from left, lateral view of rest posture; third from left, contour map of rest posture; third from right, frontal view of smile posture; second from right, lateral view from the right; right, contour map of smile posture.

The final sample consisted of a younger group (n = 100; mean, 24.5 ± 3.7 years; age range, 18–32 years) and an older group (n = 30; mean, 61.0 ± 4.8 years old; age range, 55–65 years). Fig 2 depicts the averaged and accentuated averaged faces that were computed for the older and younger groups. Comparison of those faces, particularly the accentuated averaged faces, clearly demonstrates the manner in which the older face differed from the younger face in three dimensions. From the lateral view, subjects in the older group showed a concave profile of the subnasal region, with thin lips. Interestingly, the inclination of the lip fissures at rest differed between the groups, that is, the corner of the mouth in the older group was higher and the commissure was longer, whereas the younger group showed an inferior position of the corner of the mouth and shorter lip commissures. Also, the inclination of the lip fissures showed smaller changes from rest to smiling in the older group than in the younger group. From the frontal view, the eye fissures of the subjects in the older group were vertically smaller.

Furthermore, the contour maps showed that at rest the older group possessed greater nasolabial folds, that is, grooves that ran from each side of the nose to the corners of the mouth, whereas the younger group showed smaller nasolabial fold. In the transition from rest to smiling, the nasolabial folds did not change in the older group, but in the younger group they became larger. Overall, the changes in the nasolabial fold were greater in the younger group. For the upper lip, the older group showed a transversely wider and flatter shape, whereas the younger group showed a more prominent shape. The lower lip to chin had similar characteristics to the upper lip: the younger group showed smaller but more prominent lower lips. Also, the older group had grooves that ran from each side of the corners of the mouth in the direction of the mandibular angles.

In short, the older faces at rest were very similar to the older face during smiling due to differences in the lip commissure and nasolabial fold. The older people had a transversely widened lip commissure even at rest, which was similar to the smiling features in the younger group. Furthermore, the older group has a substantial nasolabial fold even at rest, whereas the younger group showed a nasolabial fold only when smiling.

### Quantitative surface-based analysis

**Fig 3:**
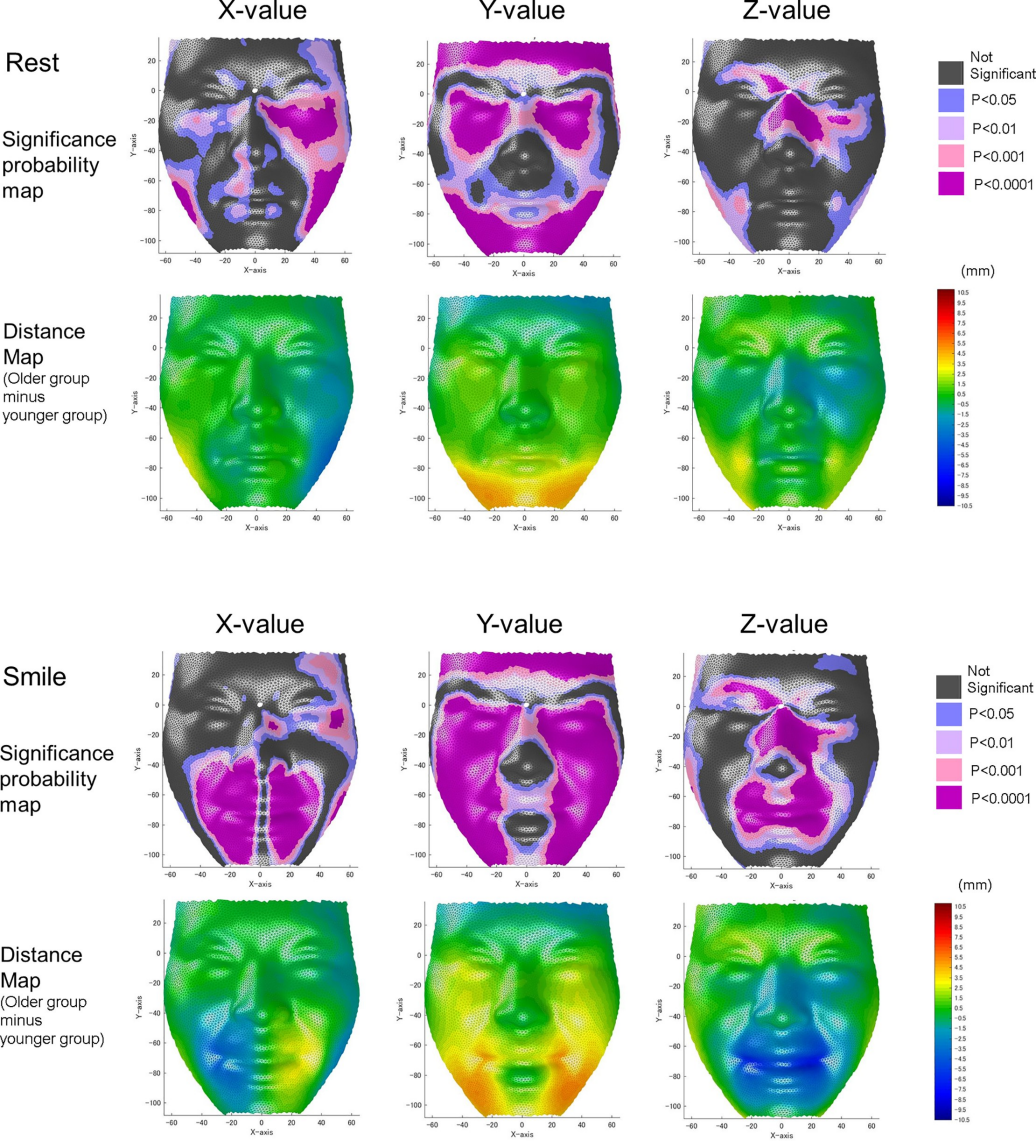
Significance maps [top (at rest) and second from the bottom (at peak of smiling)] and difference maps [younger minus older; second from the top (at rest) and bottom (at peak of smiling)]. For the significance maps, blue designates P ≤ 0.05; pale pink, P ≤ 0.01; dark pink, P ≤ 0.001; and purple, P ≤ 0.0001. For the difference maps, red indicates that the younger group exhibited greater values than the older group, whereas blue indicates that the older group exhibited greater values than the younger group. Differences are represented in mm.

**Fig 4:**
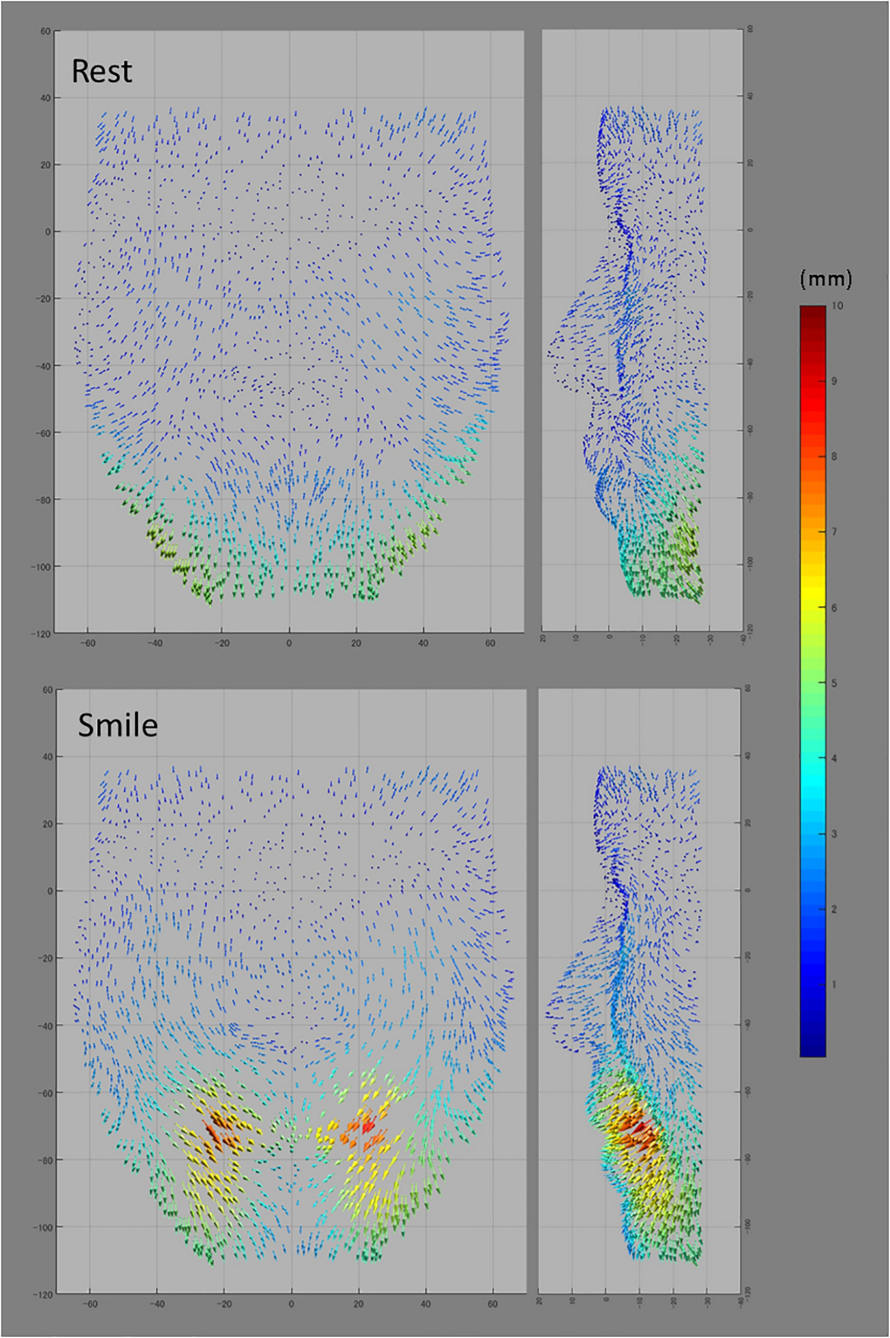
Vectors from the average mesh points of the younger group (arrow base) to those of the older group (arrow apex). Greater magnitudes are indicated by red and smaller magnitudes by blue. (Left, frontal view; right, lateral view).

Fig 3 shows significance and difference maps of the X-, Y-, and Z-values, and Fig 4 shows representative vectors of the average mesh points, for the younger and older groups.

At rest, comparison of the X-values (transverse direction) showed that the infraorbital region and gonial angle were transversely wider in the older group than in the younger group (P < 0.01), whereas the corners of the mouth were transversely smaller in the older group. Comparison of the Y-values (vertical direction) showed that the vertical positions of the upper and lower eyelids, cheeks, corners of the mouth, lower lip, and chin were located at more inferior positions in the older group than in the younger group, when the nasion was set as the origin (P < 0.01). Comparison of the Z-values (anteroposterior direction) showed that the corners of the mouth were more retruded in the older group than in the younger group (P < 0.01). In contrast, the nasal bridge and infraorbital regions were significantly more protuberant in the older group than in the younger group (P < 0.01).

At the peak of the smile, comparison of the X-values showed that the corners of the mouth were transversely smaller in the older group than in the younger group (P < 0.01). Comparison of the Y-values showed that the vertical positions of the upper eyelids, lower cheeks, upper lip, and chin were lower in the older group than in the younger group, when the nasion was set as the origin (P < 0.01). Comparison of the Z-values showed that the mouth, nasal walls, and lateral regions of the eyes were more protuberant in the older group than in the younger group (P < 0.01).

### Interactions of age and facial postures

**Fig 5:**
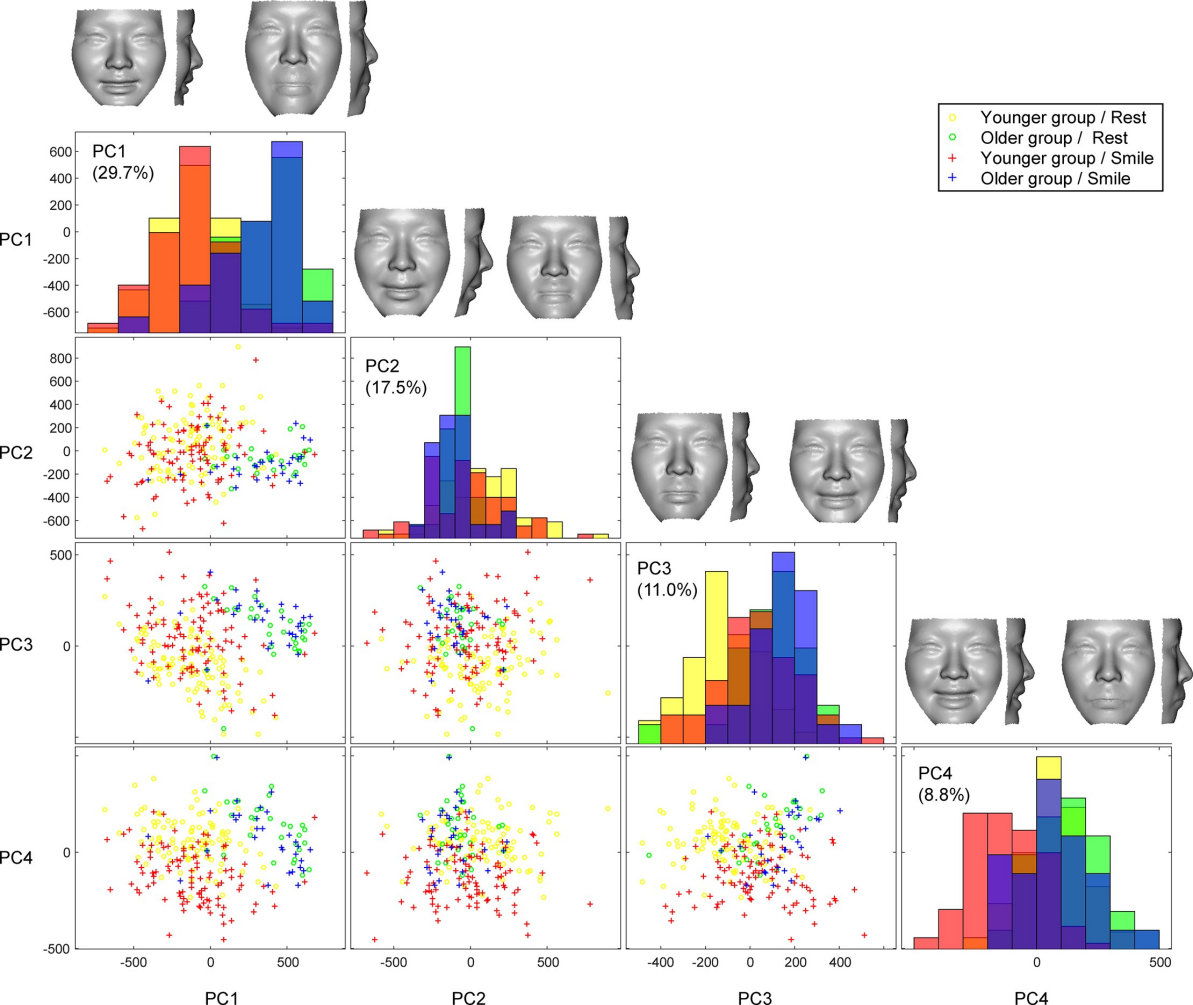
A scatter plot matrix of the principal component (PC) scores for rest and smiling postures in the younger and older groups with a histogram in diagonal cells. PCs 1–4 explains 67.0% of shape variation across samples. The PC1 shows a clear separation between age groups. Yellow denotes facial configurations at rest in younger group; green, those at rest in older group; red, those at the peak of smiling in younger group; blue, those at the peak of smiling in older group. Shape changes associated with PCs 1–4 are shown in the top column.

**Fig 6:**
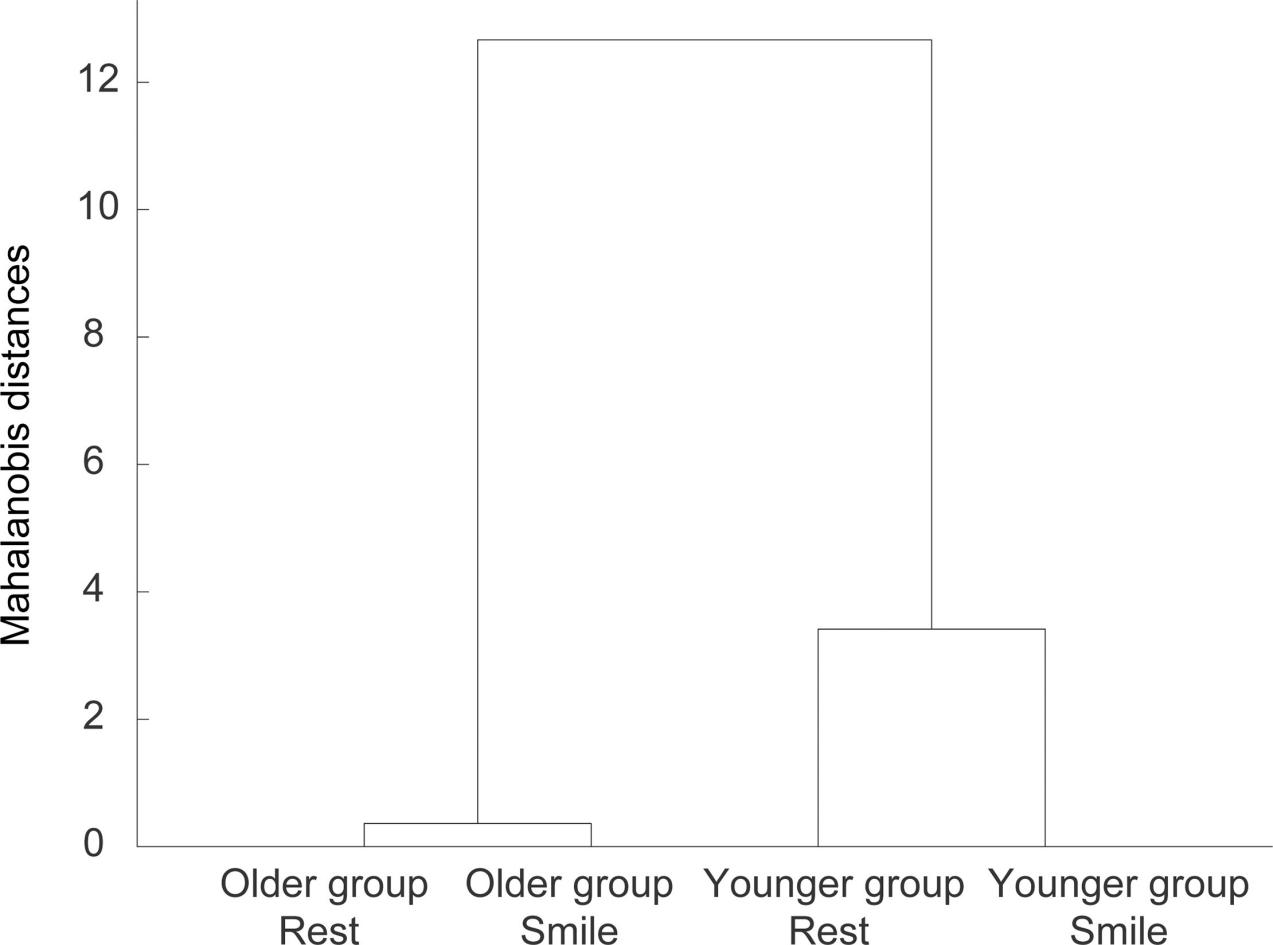
Dendrogram produced by applying the single linkage method to the matrix of Mahalanobis distances between subgroup means. X-axis, subgroups (i.e., older group and younger group / rest and smile postures); Y-axis, Mahalanobis distances between subgroup means followed by a multifactor analysis of variance (MANOVA).

Direct interactions of aging and facial postures on the 3D facial morphologies were examined using a PCA and MANOVA. The first four significant PCs, which explained 67.0% of the sample’s variance, were determined to be significant by a scree plot analysis. Visualization of the between-group structure of the surface data revealed a distinct separation between age groups (PC 1) and, to a lesser extent, a noticeable expression of facial postures (PCs 3 and 4; Fig 5). The Mahalanobis distances between the two age groups were 13.0 and 11.4 for the rest and smile postures, respectively. In contrast, the Mahalanobis distances between the rest and smile postures were 0.4 and 3.4 in the older and younger groups, respectively (Fig 6). These results indicate that the older group showed relatively smaller differences in 3D facial morphology between rest and smile facial postures in comparison to the younger group. Age and facial postures were highly significant (P<0.01, Table 1), and these two factors showed significant interaction (P<0.01). This result means that there were significant age-related 3D facial changes in facial expression generation.

**Table 1:** Multifactor analysis of variance (MANOVA) of the surface-based model.

	Df	Pillai	Approx F	Num Df	Den Df	Pr(>F)
Age	1	0.680	134.68	4	253	<2e-16*
Facial postures	1	0.380	38.75	4	253	<2e-16*
Age : Facial postures	1	0.070	4.79	4	253	0.00096*
Residuals	256					

* P<0.01

### Discriminant analysis

To describe aforementioned age-related 3D facial changes while smiling in details, we conducted sectional-line-and-landmark-based analyses and developed two types of discriminant functions: functions to discriminate between younger and older groups for each facial posture, and functions to discriminate between the resting and smiling facial postures for each age group. Eighty-three out of the 185 variables differed significantly between the groups in the rest posture, and 93 differed in the smiling posture (P < 0.01). Furthermore, 66 variables showed significant changes from resting to smiling in the older group, and 144 showed significant changes in the younger group. Thus, the younger group showed almost twice the number of variables with significant changes from rest to smile, indicating that the younger group showed greater morphological changes. Detailed results for the differences between the two groups at rest and while smiling are presented in S3–S9 Tables, S1–S9 Figs, and S2 Appendix.

### Discriminant analysis for the discrimination of young vs. older faces

All four of the obtained discriminant functions were statistically significant (P < 0.01). Of the statistically significant variables, 11 met the criteria for inclusion in the stepwise analysis for the resting condition (Table 2), and 6 met the criteria for the smiling condition (Table 3).

**Table 2:** Discriminant coefficient, standardized discriminant coefficient, partial F-value, and P-value for each of the 11 variables selected in the stepwise analysis for the discriminant function to separate faces in the older group and younger group in the resting posture. For definition of the variables, please see S1 Appendix, S1–S6 Figs.

Facial characteristics to discriminate younger vs. older groups in the resting posture	Discriminant coefficient	Standardized discriminant coefficient	Partial F-value	P-value (F-value)	Structure matrix
Greater sagging of skin in orbital and infraorbital regions in older group (Ex-Ac//z_|L1-L2|/L1^L2, right)	-0.42	-0.45	52.11	4E-11	-0.26
Greater sagging of skin in cheeks of zygomatic region in older group (Prn//axial_|P|, left)	-0.28	-0.56	18.82	3E-05	-0.16
Greater sagging of facial outline relative to the deepest point of nasofrontal region in older group (N//axial_|P|, left)	-0.16	-0.30	12.37	0.0006	-0.13
More protruded position of nasal ala relative to outer corners of eyes in older group (Ex-Ac//z_∠L1-L2, left)	-0.15	-0.61	10.47	0.0015	-0.12
Greater facial width at eyebrow level in older group (Gla//axial_|E-E|)	-0.12	-0.72	13.59	0.0003	0.13
Vertically longer subnasal region in older group (Ac-Ch//z_|L1-L2|, left)	0.20	0.61	25.06	2E-06	0.18
Greater facial width at base of lower lip level in older group (Li//axial_|E-E|)	0.12	0.95	44.85	6E-10	0.24
Flabby cheeks at the level of the chin in older group (Sm//axial_∠E-P-M, right)	0.09	0.37	45.03	6E-10	0.24
Flabby cheeks at the level of the upper lip in older group (Ls//axial_∠E-P-M, right)	0.07	0.35	47.19	3E-10	0.25
Greater sagittal protrusion of orbital and infraorbital regions in older group (En-Ac//z_∫(L1-L2))	0.19	0.29	58.28	5E-12	0.27
More convex upper lip curvature from lateral view in older group (Prn//sagittal_v12)	0.08	0.54	101.38	< 2.22e-16	0.36
Constant	6.35				

**Table 3:** Discriminant coefficient, standardized discriminant coefficient, partial F-value, and P-value for each of the 6 variables selected in the stepwise analysis for the discriminant function to separate faces in older group and younger group in the smiling posture. For definition of the variables, please see please see S1 Appendix, S1–S6 Figs.

Facial characteristics to discriminate younger vs. older groups in the smiling posture	Discriminant coefficient	Standardized discriminant coefficient	Partial F-value	P-value (F-value)	Structure matrix
Vertically longer distance between outer corners of eyes [Ex] and mouth [Ch] in older group (Ex-Ch//z_|L1-L2|, left)	-0.14	-0.65	80.61	2.97E-15	-0.46
More convex upper lip curvature from lateral view in older group (Prn//sagittal_v12)	-0.04	-0.38	51.03	6.11E-11	-0.37
More protruded upper and lower lips in older group (Prn//sagittal_v8)	-0.11	-0.48	19.53	2.09E-05	-0.23
Greater sagging of skin in orbital and infraorbital regions in older group (En-Ac//z_|L1-L2|/L1^L2)	0.52	0.67	19.06	2.59E-05	0.23
Greater sagging of facial outline at mandibular angle in older group (Facial outline_∠Go’-Gn, right)	0.12	0.46	32.15	9.02E-08	0.29
Smaller height-to-width ratio of eyes in older group (|Ps-Pi|/|Ex-En|)	9.11	0.50	40.18	3.63E-09	0.33
Constant	-54.44				

In descending order of the absolute value of the structure matrix (the correlations between the variables and the discriminant functions), at rest, the older group exhibited a straight or convex profile of the subnasal region (absolute value of the structure matrix = 0.36), greater bags under eyes (0.26 and 0.27), flabby cheeks at the level of the lips and the chin (0.25 and 0.24), greater facial width of the lower face (0.24), inferior positioned mouth (0.18), greater sagging of skin in cheeks of zygomatic region and facial outline (0.16 and 0.13), greater facial width of the forehead (0.13), and protrusion of the nasal wing (0.12).

At the peak of smiling, the older group showed an inferior position of the corner of the mouth (0.46), a convex profile of the subnasal region (0.37), a smaller eye height (0.33), sagging of the face at the mandibular angle (0.29), greater bags under eyes (0.23), and greater protrusion of the mouth (0.23).

The discriminant analysis for the face at rest generated an overall correct response rate of 100%, whereas that for the peak of smiling showed 98.4% correct responses. Both discriminant functions obtained were statistically significant (P<0.01).

### Discriminant analysis for determination of facial postures in each group

For the discriminant analysis for smiling versus resting postures, 15 variables met the criteria for inclusion in the stepwise analysis (Table 4) for the younger group, and two met the criteria for the older group (Table 5).

**Table 4:** Discriminant coefficient, standardized discriminant coefficient, partial F-value, and P-value for each of the 15 variables selected in the stepwise analysis for the discriminant function to separate faces during smiling and resting postures in the younger group.

Facial characteristics to discriminate rest vs. smile postures in the younger group	Discriminant coefficient	Standardized discriminant coefficient	Partial F-value	P-value	Structure matrix
Increase in facial width at level of the upper lip during smiling (Ls//axial_|E-E|)	-0.28	-2.11	57.65	1E-12	-0.21
Increase in cheek protrusion at level of chin during smiling (Sm//axial_∠E-P-M, right)	0.05	0.24	37.16	6E-9	-0.17
Increase in cheek protrusion in orbital regions during smiling (Ex-Ch//z_|L1-P| (left, % to |L1-L2|)	-0.02	-0.24	35.51	1E-8	-0.17
Increase in facial width at level of base of columella level during smiling (Sn//axial_|E-E|)	0.23	1.67	11.83	7E-4	-0.10
Posterior movement of nasal dorsum during smiling (N//saggital_v7)	-0.18	-0.65	2.86	9E-2	0.05
Retrusive movement of nasal ala during smiling En-Ac//z_∠L1-L2, right)	0.10	0.40	5.97	2E-2	0.07
Upward movement of eyes during smiling (|N-En|)	0.21	0.32	6.43	1E-2	0.07
Decrease in lower lip height during smiling (|Sto-Li|)	0.11	0.21	46.39	1E-10	0.19
Retrusive movement of lower lip during smiling (Li//axial_|M|)	-0.21	-0.36	77.22	7E-16	0.24
Decrease in the lower face height during smiling (|Sn-Gn|/|N-Gn|)	15.73	0.45	78.78	4E-16	0.25
Retrusive movement of upper lip during smiling Prn//saggital_v6 (%;)	-0.05	-0.18	103.92	2E-16	0.28
Decrease in the protrusion of the cheek at level of lower lip during smiling (Li//axial_|P|, right)	0.32	0.47	174.71	2E-16	0.37
Decrease in the labio-mental fold during smiling (Prn//saggital_v11)	0.18	0.57	192.49	2E-16	0.38
Retrusive movement of lip commissure relative to nasal ala during smiling (Ac-Ch//z_∠L1-L2, right)	0.09	0.54	378.42	2E-16	0.54
Constant	-3.01				

**Table 5:** Discriminant coefficient, standardized discriminant coefficient, partial F-value, and P-value for each of the 2 variables selected in the stepwise analysis for the discriminant function to separate faces during smiling and resting in the older group.

Facial characteristics to discriminate rest vs. smile postures in the older group	Discriminant coefficient	Standardized discriminant coefficient	Partial F-value	P-value	Structure matrix
Decrease in lower face height during smiling (|Sn-Gn|/|N-Gn|)	-0.39	-0.81	35.13	2E-7	-0.81
Cheek protrusive movement in the buccal and oral regions during smiling (Ex-Ch//z_L1-L2|/L1^L2, left)	-19.34	-0.58	18.26	7E-5	-0.59
Constant	47.27				

In descending order of the absolute value of the structure matrix (the correlations between the variables and the discriminant functions), the younger group exhibited a significant retrusive movement of the lip commissure and the upper lip from rest to smile (0.54 and 0.28), a significant decrease in the labio-mental fold from rest to smile (0.38), a significant retrusive movement of the lower lip during smiling (0.37), a significant decrease in the lower face height from rest to smile (0.25), a significant decrease in the protrusion of the cheek during smiling at the level of the lower lip and at the chin (0.24 and 0.17), a significant increase in the facial width during smiling at the base of the upper lip level and at the base of the columella level (0.21 and 0.10), a significant lower lip height reduction from rest to smile (0.19), a significant increased cheek protrusion during smiling (0.17), a significant upward movement of the eyes during smiling (0.07), a significant retrusive movement of nasal ala during smiling (0.07), and the nasal dorsum inclined posteriorly (0.05).

In contrast, the older group exhibited a significant decrease in the lower face height from rest to smile (0.81) and a significant cheek protrusion from rest to smile (0.59).

The overall correct response rate of the discriminant analysis for facial postures was 99% in the younger group (99/100 for the resting condition and 99/100 for the smiling condition) with 15 variables, and 80% in the older group with two variables. This result suggests that the discrimination of smiling and resting faces was more difficult in the older group, and relied on a smaller number of morphological characteristics. In detail, for the older group 76.6% (23/30) of smile postures of older group were categorized correctly whereas 23.3% (7/30) were categorized incorrectly; 83.3% (25/30) of rest postures were categorized correctly whereas 16.7% (5/30) were categorized incorrectly. These results were consistent with those of the surface-based analyses that were performed with a MANOVA.

## Discussion

In the present study, to clarify the cause of facial expression decoding problems in elderly people, we focused on the 3D facial morphology at rest and at the peak of smiling. A MANOVA and discriminant analyses showed that there were significant age-related 3D facial changes in facial expression generation.

### Discriminant analysis between the younger and older groups

In the present study, 3D measurements of the human face were obtained to evaluate morphological differences between the faces of young and older adults at rest and at the peak of smiling. The age-related changes of the face at rest can be summarized in terms of three main characteristics: (1) vertically downward changes, including larger bags under the eyes, smaller height of the eye fissures, greater lower facial height, greater length of the subnasal region, and inferiorly positioned eyes, cheek, and mouth; (2) transverse widening changes, including greater facial width of the forehead and the lower face, and greater nasal width; and (3) protrusive changes of the infraorbital region, and a straight or convex profile of the subnasal region. These characteristics are consistent with qualitative observations in the field of cosmetic surgery [19–20] and with a two-dimensional study [21] that reported an overall inferolateral displacement of points with aging, including the lateral commissure, alar rim, and maximal projection of the cheek mass.

It was found that during the transition from resting to smiling, these age-related characteristics were largely maintained. However, the older group showed smaller movements of the eyes, nose, and cheek when compared with the younger group; thus sagging of the face at the mandibular angle was accentuated during smiling in the older group. With these characteristics, the discriminant analysis for the face at rest generated an overall correct response rate of 100%, whereas during smiling the rate was 98.4%. This result indicates that the facial morphology was distinctly different in the younger and older subjects for both facial expressions.

### Discriminant analysis between resting and smiling facial postures

We also found that the older group produced a 20% error rate in discriminating between resting and smiling facial postures, with only 2 of 66 variables making significant contributions, whereas the younger group showed only a 1% error rate, with 15 of 144 variables making significant contributions. The number of variables showing significant differences in the older group was almost half (66/144) that in the younger group, and the number of variables that were used in the discriminant functions was only 13% (2/15) of that in the older group. The characteristics most useful for discriminating an older smiling face from an older resting face were a significant decrease in the lower face height at the peak of smiling and a significant sagittal protrusive movement of the cheek in the buccal and oral regions at the peak of smiling. This result indicates that the facial morphology during smiling of the older people contains fewer morphological clues to the presence of a smile. Accentuated averaged older faces also enabled us to see that the older face at rest was very similar to the older face during smiling due to the transversely widened lip commissure and the greater nasolabial fold at rest. Thus, it can be said that in the older group, smiling faces could be more easily misunderstood as resting faces and vice versa. The MANOVA results also showed that there were significant age-related 3D facial changes in facial expression generation and this can be considered as a functional decline in facial expression generation in older people.

### Mechanisms of facial aging

In general, facial configurations are determined by the skin, subcutaneous fat, muscle, and underlying skeletal shape. Current and accepted mechanisms of facial aging involve all these external and intrinsic layers and their interactions. Of these, intrinsic aging of the subcutaneous fat, muscle, and bone are believed to be hormonally and genetically regulated, and are responsible for bone and soft tissue remodeling [20].

### Intrinsic layers: Muscles and nerves

#### Corner of the mouth

With regard to intrinsic aging effects, the present study showed that the corners of the mouth showed a significantly smaller backward and upward movement from the resting to smiling positions in the older group than in the younger group, whereas the vertical movement of the upper lip showed no significant difference between the two groups. These movements may be attributed to the actions of the levator labii superioris and the zygomaticus major, which originate from the zygomatic bones and end onto the upper lip and the modiolus of the mouth, respectively. Theoretically, three factors could affect the actions of the levator labii superioris and the zygomaticus major: loss of muscle mass, changes in the structure of the muscles, and changes in the nervous systems. Each of these may come into play.

Concerning muscle mass, there is controversy concerning atrophy of the mimetic muscles with age. A magnetic resonance imaging (MRI) study found that there was no significant difference between young and elderly subjects in the length or thickness of the levator labii superioris or the zygomaticus muscles at rest [22].

Concerning muscle structure, increased fatty infiltration in elderly women has been suggested [23]. A previous study suggested that the metabolic mechanisms within the skeletal muscle that regulate some aspects of lipid metabolism are potentially dysfunctional under conditions of reduced estrogen levels with aging [24]. Age-related changes in the prevalence and magnitude of the intramuscular fat are likely to contribute to the impaired production of muscle force [25]. In general, there are two types of muscles, “fast-twitch” (Type IIa, IIb) and “slow-twitch” (Type I) myosin fibers. The zygomaticus major in humans contains 60% fast-twitch fibers and 15% slow-twitch fibers. Slow-twitch fibers in humans tend to accumulate more intramuscular fat than fast-twitch oxidative fibers [26–27].

Concerning the nervous system, changes that accompany cranial and peripheral motor denervation were previously described as they affect muscle volume and fatty infiltration of the involved muscle groups [28]. Motor performance deficits in elderly adults may be caused by dysfunction of the central and peripheral nervous systems and the neuromuscular system [29]. Thus, it is reasonable to state that the dysfunction of the nerves with age may lead to fatty infiltration of the mimetic muscles and a significantly smaller amount of the lateral and upward movement of the corner of the mouth.

#### Eyes and eyelids

In the present study, it was also found that during a smile the younger group showed significant upward movement of the eyes, nose, cheek, and gonial angle, whereas the older group showed no significant vertical movement in this area. Upward movement of the eyes and nose may be attributed to the actions of the occipitofrontalis muscle and the levator labii superioris alaeque nasi muscle. In changing from resting to smiling postures, the older group showed a significantly greater decrease in eye height than the younger group; hence it may be assumed that the occipitofrontalis muscle in the older group showed decreased movement.

Further, during smiling, bulging eyelids were observed in the older group. A previous study using a computed tomography method [30] showed that the orbicularis oculi muscle was significantly thinner in the older group. Likewise, orbital fat prolapse was found to be significantly more prominent in the older group.

### External layer (skin) and its interaction with the intrinsic layer

#### Cheek and gonial angle

Regarding the smaller movement of the cheeks and gonial angle, skin aging may be responsible for this phenomenon. Skin aging is thought to be caused by ultraviolet light-induced and oxidative damage to the skin. A study found that facial skin aging was characterized by a progressive increase in extensibility associated with a decreased elasticity and a loss of tonicity, accompanied by progressive deepening of the facial creases [31]. Repeated facial animation over time, in conjunction with chronic sun exposure, may permanently cause skin and muscle fibrosis and initiate the onset of the dermal component of wrinkling [32].

The interaction of the skin and muscle also relate to the smaller movement of the cheek and gonial angle. The subcutaneous fat serves to weaken the muscular pull on the skin and acts as a glide plane between the skin and the muscle [20]. A study involving MRI showed that the thickness of the cheek fat pad was significantly greater in elderly subjects, and it was concluded that differential descent of the cheek mass with respect to the upper lip contributed to the deepening of the nasolabial fold with aging [22].

### Craniofacial skeletal remodeling

Craniofacial skeletal aging is also associated with aging. This includes changes in the contour of the maxilla, an increase in vertical maxillary dimension with retrusion of the lower maxillary skeleton, slight overall widening of the face, and a decrease in overall facial height [33]. In the present study, however, we could not identify a decrease in overall facial height. We suppose that this may be due to the abundant cervical fatty tissues in elderly people [34]. Analysis of anterior-posterior changes on computed tomography showed that there was a tendency for the lower maxillary skeleton at the pyriform to become retrusive with age relative to the upper face [35], indicating that a relative maxillary retrusion might be a factor in the development of the nasolabial fold in elderly people, which was observed in the present study.

### Limitations

Our study has several limitations. First, the study was limited by the use of only women. As previous studies found no sex differences in facial motions during smiling (except for greater movement in men as a result of size differences [36, 37]), it is likely that the results of the present study are also applicable to men, but further research are needed with male subjects. Second, our subject population had limited variation in age range. Current results may give some bias because facial forms cannot necessarily be explained as a linear function of chronological age, and thus should be interpreted cautiously. Those factors such as extremely biased nutritional intake environment or excessive exposure dose to ultraviolet rays may also be considered. Yet, the results will provide us with further insights into our better understanding of age-related communication dysfunction by comparing two different age groups, one representing young adults over the age 18, and the other group with an age range between 55 and 65 years, both city dwellers with no psychiatric disorder.

## Supporting information

S1 Appendix Sectional-line-and-landmark-based analysis. (DOCX)

S2 Appendix Results of the sectional-line-and-landmark-based analysis. (DOCX)

S1 Table Definitions of the soft tissue landmarks on the facial 3D images. (DOCX)

S2 Table Five types of curving lines. (DOCX)

S3 Table Result summary of facial characteristics unique to the older group when compared with the younger group at rest and on smiling. (DOCX)

S4 Table Means and their standard deviations (S.D.) of the 29 inter-landmark distances that had been reported in previous studies and the height-to-width ratio of the outline of the supraorbital ridge for each group. (DOCX)

S5 Table Means and their standard deviations (S.D.) of the 15 ratios that had been reported in previous studies and the height-to-width ratio of the outline of the supraorbital ridge for each group. (DOCX)

S6 Table Means and their standard deviations (S.D.) for the 28 variables for the contours Ex-Ac//z, En-Ac//z, Ex-Ch//z, and Ac-Ch//z for each side. (DOCX)

S7 Table Means and their standard deviations (S.D.) for the 21 variables for the contours N//sagittal and Prn//sagittal. (DOCX)

S8 Table Means and their standard deviations (S.D.) for the 58 variables for the contours Gla//axial, N//axial, Or//axial, Prn//axial, Sn//axial, Ls//axial, Li//axial, and Sm//axial. (DOCX)

S9 Table Means and their standard deviations (S.D.) for the 3 variables for the Facial outline contours for each side. (DOCX)

S1 Fig The coordinate system and landmarks used in the present study. (DOCX)

S2 Fig Schematic diagram illustrating the measurements for the contours Ex-Ac//z, En-Ac//z, Ex-Ch//z, and Ac-Ch//z. (DOCX)

S3 Fig Schematic diagram illustrating vector elements v1, v2, v3, v4, v5, v6, v7, and v8 of N//sagittal (i.e., the nasal profile). (DOCX)

S4 Fig Measurements for the contour Prn//sagittal (i.e., naso-lip-chin profile). (DOCX)

S5 Fig Schematic diagrams illustrating the measurements of the contours Gla//axial, N//axial, Sn//axial, Ls//axial, Li//axial, Sm//axial, Or//axial, and Prn//axial. (DOCX)

S6 Fig Schematic diagram illustrating the linear and angular measurements of the facial outline. (DOCX)

S7 Fig The mean contours of sagittal sections (N//sagittal and Prn//sagittal), mean facial outlines, and mean coordinates of the landmarks (Gla, Ex, En, Ps, Pi, Prn, Sn, Ls, Sto, Li, Ch, Pog, and Zy). (DOCX)

S8 Fig The mean contours of Ex-Ac//z, En-Ac//z, Ex-Ch//z, and Ac-Ch//z on the right and left sides in the older group and the younger group. (DOCX)

S9 Fig The mean contours of Gla//axial, N//axial, Or//axial, Prn//axial, Sn//axial, Ls//axial, Li//axial, and Sm//axial in the older group and the younger group. (DOCX)
