# Supplementary material for: Functional decline in facial expression generation in older women: A cross-sectional study using three-dimensional morphometry
Source: PLoS One. 2019 Jul 10;14(7):e0219451. doi: 10.1371/journal.pone.0219451 (PMC6636602; doi:10.1371/journal.pone.0219451)
Supplement: S1 Appendix — (DOCX) [file pone.0219451.s001.docx]

**S1 Appendix: Sectional-line-and-landmark-based analysis**

The facial morphology was examined by extracting 5 categories of curving lines from the 3-D images (S2 Table):

1. Section of the inter-landmark contour: 4 curving lines defined as the cross-sectional lines of the 3-D image surface and the plane passing through 2 landmarks (L_1_ and L_2_ in S2 Table) and parallel to the Z-axis
2. Sagittal section: 2 curving lines defined as the cross-sectional lines of the 3-D image surface and the plane passing through 1 landmark (L_1_ in S2 Table) and parallel to the Z-Y plane
3. Axial section: 8 curving lines defined as the cross-sectional lines of the 3-D image surface and the plane passing through 1 landmark (L_1_ in S2 Table) and parallel to the Z-X plane
4. Facial outline: the outer border of the face was extracted as a series of surface points with surface inclinations at 60° angles to the Z-axis. Please see the Appendix for details.
5. Supraorbital ridge outline: the shape of the supraorbital ridge was extracted from the contour map of the face as a series of the surface points that exhibited 1 mm of retrusion from the most prominent point between the eyebrows (Glabella).

The curving lines described in the categories 1), 2), 3), and 4) were used to extract 141 measurements as described in S2, S3, S4, S5, and S6 Figs. The curving line of the category 5) was used to define 1 measurement, the height-to-width ratio of the outline of the supraorbital ridge. In addition, 28 inter-landmark distances and 15 ratios that had been reported in previous studies (Farkas and Munro, 1987; Sarver, 1998, Carre et al., 2009) were determined and employed (See S3 Table for a list of these variables.). Therefore, 185 variables were employed in total. The facial size differences were standardized by normalizing the values of all of the linear variables to the Ex-Ex distance.
