# Supplementary material for: Functional decline in facial expression generation in older women: A cross-sectional study using three-dimensional morphometry
Source: PLoS One. 2019 Jul 10;14(7):e0219451. doi: 10.1371/journal.pone.0219451 (PMC6636602; doi:10.1371/journal.pone.0219451)
Supplement: S2 Appendix — (DOCX) [file pone.0219451.s002.docx]

**S2 Appendix: Results of the sectional-line-and-landmark-based analysis**

Results of the sectional-line-and-landmark-based analysis are summarized in S3 Table. Detailed results are presented in S7, S8, and S9 Figs; and S4, S5, S6, S7, S8, and S9 Tables.

**Inter-landmark distances** (S7 Figs, S4 and S5 Tables)

***X-value.*** At rest, the distance between the eyes (|Ex-Ex|) was significantly smaller in the older group than in the younger group (P < 0.001). Because the older group showed no significant changes from rest to smile whereas the younger group shows significant increase of the distance from resting to smiling (P < 0.001), the distance between the eyes was still significantly smaller in the older group even at the peak of smiling (P < 0.01).

The width of the nose (|Ac-Ac|) at rest was greater in the older group than in the younger group, suggesting a greater nasal width in the older group at rest and at the peak of smiling. Both groups showed a significant increase in the width of the nose; the younger group showed a greater widening movement from rest to smile than the older group (P < 0.001).

As for the width of the mouth (|Ch-Ch|) and the mouth-face index (|Ch-Ch|/|Zy’-Zy’|), the values at rest showed opposite results to those at the peak of smiling. These variables were greater in the older group at rest, and smaller at the peak of smiling in the older group than in the younger group (P < 0.001), suggesting that the mouth was transversely greater in size in the older group at rest and transversely smaller in size in the older group at the peak of smiling. This is due to a significantly smaller widening movement of the corners of the mouth (by approximately 8 mm) in the older group than in the younger group (|Ch-Ch|, P < 0.001).

The width of the gonial angle (|Go’-Go’|, |Go’-Go’|/|Zy’-Zy’|) was greater in the older group than in the younger group at rest and at the peak of smiling. This suggests that the gonial angle was horizontally greater in the older group, i.e., the older group showed sagging of the face both at rest and at the peak of smiling. As for the changes from rest to smile, both the older and the younger group showed significant increases of the gonial width. However, with regard to the degree of the increase, the older group showed a significant smaller degree of the increase compared with the younger group (|Go’-Go’|, |N-Gn|/|Go’-Go’|, P < 0.001).

***Y-value.*** When the deepest point of the nasofrontal region (nasion) in the sagittal plane was set as the origin, the positions of eyes, nose, upper lip, mouth, and chin (Rest: |N-En|, |N-Prn|, |N-Ls|, |N-Sn|, |N-Zy|, |N-Sto|, |N-Gn|; Smile: |N-En|, |N-Prn|, |N-Ls|, |N-Sto|, |N-Gn|) were vertically lower in the older group than in the younger group in both the resting and smiling postures (P < 0.01, S3 Table) When the glabella was set as the origin, the position of the lips was also lower in the older group than in the younger group in both the resting and smiling postures (|Gla-Ls|, |Gla-Sto|, P < 0.001). From rest to smile, the eyes, nose, and cheek showed significant upward movement in the younger group, whereas the older group showed no significant vertical movements (|Gla-En|, |Gla-Sn|, |Gla-Zy|, |N-En|, |N-Zy|, P < 0.01).

The upper lip moved significantly upward from rest to smile in both groups, by approximately 3-4 mm and 5 mm in the older and younger group, respectively (|Gla-Ls|, |Gla-Sto|, |N-Ls|, |N-Sto|, P < 0.01). There were no significant differences between the groups regarding the upward movement of the upper lip when the glabella and the nasion were set as the origin. Further, the vertical distance between the subnasale and the inferior border of the upper lip (|Sn-Sto|) was greater in the older group for both facial postures, and this value was significantly decreased from rest to smile (P < 0.001), indicating upward movement of the upper lip from rest to smiling. The degree of the change in this distance (|Sn-Sto|) was smaller in the older group than in the younger group, suggesting a smaller upward movement of the upper lip relative to the nose.

With regard to the nose, the nasal bridge length was greater in the older group than in the younger group both at rest and smiling (|N-Prn|, P<0.001). Both groups showed significant upward movement of the nasal tip from rest to smile (|N-Prn|, P < 0.001) and there were no significant differences between the groups regarding the upward movement of the nasal tip from rest to smile.

As for the chin height (|Go’-Gn|), there were no significant differences between the groups at rest, whereas at the peak of smiling the older group showed significantly greater chin height than the younger group (P < 0.001). Both groups showed significant increases from rest to smile in chin height (P < 0.001), but the younger group showed a greater increase in chin height from rest to smile (P < 0.001), indicating a greater upward movement of the gonion from rest to smile in the younger group.

The vertical height of the eye fissures (|Ps-Pi|) and the height-to-width ratio of the eyes (|Ps-Pi|/|Ex-En|) were smaller in the older group than in the younger group (P < 0.001), suggesting that the older women’s eye fissures were vertically smaller than the younger women’s in both resting and smiling postures. From rest to smile, the older group showed a significantly greater decrease in eye height than the younger group (|Ps-Pi|, P < 0.01).

The heights of the upper and lower lip vermilions (|Ls-Sto|, |Sto-Li|) did not differ significantly between the groups (P > 0.01) at rest or at the peak of smiling; however only the younger group showed a significant decrease in the upper lip vermilion height from rest to smile (|Ls-Sto|, P < 0.01).

The lower facial height (|Sn-Gn|) was significantly longer in the older group than in the younger group at the both facial postures. From rest to smile, this value showed a decrease in both groups.

***Height-to-height ratio.*** The mandible-upper face height index (|Sto-Gn|/|N-Sto|), mandible-lower face height index (|Sto-Gn|/|Sn-Gn|), and chin-lower face height index (|Sm-Gn|/|Sn-Gn|) were all significantly smaller in the older group for both facial postures (P < 0.001). These are related to the greater upper and lower face height in the older group, whereas the mandible height (|Sto-Gn|) did not differ significantly between the groups (P > 0.01).

With regard of the vertical position of the cheek, the older group showed a significantly smaller downward movement from rest to smile than the younger group (|N-Zy|, P<0.01)

***Height-to-width ratio.*** The facial index (|N-Gn|/|Zy′-Zy′|) and upper face index (|N-Sto|/|Zy’-Zy’|) were significantly greater in the older group for both facial postures (P < 0.01), suggesting longer vertical face height and upper face height in the older group. The ratio of the total upper face height to the zygomatic width (|Gla-Sto|/|Zy’-Zy’|, |Gla-Ls|/|Zy′-Zy′|) were also significantly greater in the older group for both facial postures (P < 0.01).

**Inter-landmark sections (S6 Table, S8 Fig)**

***Ex-Ac//z (Inter-landmark contour of Ex and Ac parallel to the Z-axis; the orbital and infraorbital regions)****.* The angle formed by the line connecting the outer corners of the eyes (Ex), the nasal ala (Ac) and the Y-axis (∠L_1_-L_2_) was smaller in the older group than in the younger group (left; P < 0.01) for both facial postures, suggesting the nasal ala was anterior-posteriorly retruded in the older group. The distance between Ex and Ac showed no differences between the groups at rest, but was vertically longer at the peak of smiling in the older group than in the younger group (|L_1_-L_2_|, P < 0.01), suggesting that the nasal ala showed smaller movement from the resting to the smiling posture in the older group. The ratio of the distance between Ex and Ac to the length of the contour along its curvature (|L_1_-L_2_|/L_1_^L_2_) was smaller in the older group than in the younger group (P < 0.01), suggesting that the sagging of skin in the orbital and infraorbital regions was greater in the older group at rest. When smiling, this value showed a significant decrease in the younger group, i.e., protrusion of the cheek (P < 0.001), and as a result, there were no differences in either group in this value when smiling. Both groups also showed decreases of the distance between Ex and Ac (|L_1_-L_2_|) from resting to smiling as well as an increase in the area enclosed by the line connecting Ex and Ac and the facial surface contour (∫(L_1_-L_2_)) and the distance between the line Ex-Ac and the most prominent point on the facial surface contour (|P|, P < 0.001).

***En-Ac//z (Inter-landmark contour of En and Ac parallel to the Z-axis; the orbital and infraorbital regions).*** The line connecting the inner corners of the eyes (En) and the nose (Ac) was vertically longer in the older group than in the younger group at rest and at the peak of the smile (|L_1_-L_2_|, P < 0.001). The area enclosed by the line connecting En and Ac and the facial surface contour (∫(L_1_-L_2_)) and the distance between the line En-Ac and the most prominent point on the facial surface contour (|P|) were greater in the older group than in the younger group (P < 0.001). The ratio of the distance between En and Ac to the length of the contour along its curvature (|L_1_-L_2_|/L_1_^L_2_) was smaller in the older group than in the younger group (P < 0.001). The angle formed by the line connecting En and the most prominent point of the infraorbital regions and the Y-axis was greater in the older group than in the younger group (∠L_1_-P, left, P < 0.01) for both facial postures. These results mean that the sagittal protrusion of the orbital and infraorbital regions was greater in the older group. The angle formed by the line connecting the inner corners of the eyes (En) and the nasal ala (Ac) was significantly decreased from rest to smile smiling in the younger group (∠L_1_- L_2_, right, P < 0.001), suggesting greater retrusive movement of the nasal ala relative to the inner corner of the eyes from rest to smiling in the younger group.

***Ex-Ch//z (Inter-landmark contour of Ex and Ch parallel to the Z-axis; the orbital, infraorbital, buccal, and oral regions).*** The line connecting the outer corners of the eyes (Ex) and the mouth (Ch) was vertically longer in the older group than in the younger group (|L_1_-L_2_|, P < 0.001). The area enclosed by the line connecting Ex and Ch and the facial surface contour (∫(L_1_-L_2_)), and the distance between the line Ex-Ch and the most prominent point on the facial surface contour (|P|), were smaller in the older group than in the younger group (P < 0.01) at the peak of the smile and greater in the older group (P<0.01) than in the younger group at rest, which means that the older group showed a greater sagittal cheek protrusion in the posterior part of the infraorbital region at rest and that this changed to a smaller cheek protrusion than in the younger group in the smiling state. The angle formed by the line connecting Ex and the most prominent point and the Y-axis (∠L_1_-L_2_) was smaller (rest, P < 0.001) in the older group than in the younger group. This means that the sagittal cheek protrusion in the posterior part of the infraorbital region was smaller in the older group. The ratio of the distance between Ex and Ch to the length of the contour along its curvature (|L1-L2|/L1^L2) was smaller in the older group than in the younger group (left, rest; P < 0.001), suggesting a greater sagittal protrusion of the oral regions at rest in the older group. The angle formed by the line connecting Ex and the most prominent point of the infraorbital regions and the Y-axis (∠L_1_-P) was smaller at the peak of smiling in the older group than in the younger group (left, P < 0.01), indicating flatter cheeks in the orbital regions when smiling.

As for the changes from rest to smile, almost all the variables in the Ex-Ch//z contours were significantly changed in both groups, i.e., the corner of the mouth moved to the posterior (∠L1-L2) and upwards (|L1-L2|) with a significant cheek movement (∫(L1-L2), ∠L1-P, |L1-L2|/L1^L2) from rest to smile. However, only one variable of |L1-P|, the distance from Ex to the most prominent point of the infraorbital region, did not show significant changes from rest to smile in the older group, whereas the younger group showed significant downward movement of the cheek prominence (P < 0.001).

The older group showed a significantly smaller movement of the corner of the mouth both vertically and anterior-posteriorly (∠L1-L2 and |L1-L2|, P < 0.001). The increase of the anterior-posterior height of the most prominent point on the facial surface contour (|P| and |L1-L2|/L1^L2 (%)) from rest to smile was significantly smaller in the older group than in the younger group (P < 0.001).

***Ac-Ch//z (Inter-landmark contour of Ac and Ch parallel to the Z-axis; subnasal region).*** The distance between Ac and Ch (|L_1_-L_2_|) was greater by approximately 3–4 mm in the older group than in the younger group, which suggests a vertically longer subnasal region in the older group (P < 0.001). The angle formed by the line connecting Ac and Ch and the Y-axis (∠L_1_-L_2_) was greater in the older group than in the younger group on the left side at rest. This means that the upper lip was more retruded in the older group at rest (P < 0.001). The variable ∠L_1_-L_2_ decreased significantly from rest to smile, by approximately by 7° and 17° in the older and younger groups, respectively. These results mean that the upper lip moved more posteriorly from rest to smile in the younger group, whereas the older group showed a posterior posture of the corner of the mouth already at rest and showed smaller changes from rest to smile. The area enclosed by the line connecting Ac and Ch and the facial surface contour (∫(L1-L2)), and the distance between the line Ac-Ch and the most prominent point on the facial surface contour (|P|), were greater in the older group than in the younger group (P < 0.01). The ratio of the distance between En and Ac to the length of the contour along its curvature (|L1-L2|/L1^L2) was smaller in the older group than in the younger group (P < 0.01). The angle formed by the line connecting En and the most prominent point of the infraorbital regions and the Y-axis was greater in the older group than in the younger group (∠L1-P, P < 0.01) at the peak of smiling. These results indicate that the sagittal protrusion of the subnasal regions was greater in the older group.

The older group showed a significantly smaller movement of the corner of the mouth in the anterior-posterior direction (∠L1-L2, P < 0.001).

**Sagittal sections (S7 Table, S7 Fig)**

***N//sagittal (nasal profile).*** The value of variable v5 was smaller in the older group than in the younger group at rest and at the peak of smiling, suggesting that the nasal columella as seen in the lateral view was shorter in the older group than in the younger group (P < 0.001). The value of variable v6 was also greater in the older group than in the younger group at rest, indicating a rounder nasal tip related to the aforementioned smaller columella in the older group (at rest, P < 0.01). The values of variables v7 and v8 increased significantly from rest to smile in the younger group, whereas the older group showed no changes, suggesting that the nasal dorsum and nasal columella inclined posteriorly from rest to smiling in the younger group (P < 0.001).

***Prn//sagittal (naso-lip-chin profile).*** At rest, the value of variable v3 (labio-mental angle) was greater and that of v11 (the depth of the labio-mental fold) was smaller at rest in the older group than in the younger group (P < 0.001). From rest to smile, significant increases of the labio-mental angle (v3) by approximately 4.6° and 21.6° and significant decreases of the depth of the labio-mental fold (v11) by approximately 1.3 mm and 6.1 mm were observed in the older and younger groups, respectively (P < 0.001). The variables v3 and v6 showed significantly greater changes in the younger group than in the older group. As for the antero-posterior position of the labio-mental fold (v10), only the younger group showed greater retrusive movement from rest to smile. These results indicate that both older and younger groups showed significant changes of the labio-mental fold, but the degree of the changes was greater in the younger group than in the older group. Consequently, at the peak of the smile, the variable v3 was greater and v11 was smaller in the younger group than in the older group. These results suggest that the labio-mental fold was shallower at rest but deeper at the peak of the smile in the older group than in the younger group (P < 0.001). The value of variable v5 was greater in the older group than in the younger group, suggesting greater subnasal vertical length in the older group (P < 0.001).

The values of variables v6, v7 and v8 were greater at the peak of the smile in the older group than in the younger group, suggesting that the upper and lower lips were significantly protruded in the older group compared with the younger group at the peak of the smile (P < 0.001). The variable v9 was smaller in the older group than in the younger group, indicating a less protuberant lower lip relative to its upper counterpart in the older group at the peak of the smile.

The value of variable v12 was greater in the older group than in the younger group, suggesting a more convex upper lip curvature as seen in the lateral view in the older group in both facial expressions (P < 0.001). The value of variable v5 showed decreases from smile to rest in both groups, suggesting that the upper moved significantly upwards from rest to smile in the younger group (P < 0.001).

The values of variables v6, v7, and v8 also showed decreases from smile to rest in both groups, suggesting that the upper and lower lips moved significantly backwards from rest to smile in the younger group (P < 0.001). However, the value of v9 (defined by the value of v7 – v6) showed a significant decrease only in the younger group, which indicates that the younger group showed greater retrusive movement of the upper lip from rest to smiling compared with the lower lip. With regard to the degree of the retrusive movement of the upper and lower lip (v6, v7, v8), the younger group showed a greater displacement from rest to smile (P < 0.001)

**Transverse sections (S8 Table, S9 Fig)**

At rest, the main significant differences between the older and younger groups were observed in the transverse sections at the upper lip vermilion and lower lip vermilions. In contrast, at the peak of the smile, the main significant differences were in the transverse section at the tip of the nose. The details are as follows.

***Gla//axial (transverse section at the eyebrows).*** The value of variable |E-E| was greater in the older group than in the younger group, indicating a greater facial width at the eyebrow level in the older group. The value of variable ∠E-P-M was smaller in the older group than in the younger group, indicating that the horizontal hangover of the forehead was greater in the older group at rest (P < 0.01, at rest, right). From rest to smile, transversely, older group showed no changes in the value of variable |E-E| (the facial width at the eyebrow level), whereas the younger group showed a significant decrease (P<0.001). Antero-posteriorly, older group showed protrusive movement of the forehead (|M| and |P|), whereas younger group showed no differences. Thus, the forehead morphological changes from rest to smile were characterized the antero-posterior direction in the older group instead of the transverse changes.

***N//axial (transverse section at the upper limit of the nose).*** The values of variable |M| and |P| were smaller in the older group than in the younger group at rest; that is, the facial outline defined as a series of surface points with surface inclinations at 60° angles to the Z-axis located in the forward position relative to the deepest point of the nasofrontal region in the older group (P < 0.001), indicating a sagging of the facial outline in the older group. From rest to smile, the values of variable |M| and |P| showed a decrease in only the younger group, indicating that only younger group showed greater changes in the facial outline from rest to smiling.

***Or//axial (transverse section at the orbital).*** The value of variable |E-E| was greater in the older group than in the younger group, indicating a greater facial width at the orbital level (P < 0.01). The value of variable |N-N| was greater in the older group than in the younger group at the peak of smiling, which means that the protrusion of the nasal dorsum at the orbital level was transversely greater in the older group (P < 0.001). From rest to smile, the value of variable ∠E-P-N and |M| showed significant decrease and |P| showed significant increase in both groups (right, P<0.01), indicating an increased cheek in the infraorbital region while smiling in both groups (P<0.01)

***Prn//axial (transverse section at the tip of the nose).*** The value of variable |E-E| was greater in the older group than in the younger group at both facial postures, indicating a greater facial width at the nasal tip level (P < 0.001). The antero-posterior protrusion of the nose (|M|) showed no significant differences at the both facial postures (P > 0.01), but from the rest to the smile, the value significantly decreased (P < 0.001), indicating the nasal tip moved backwards from rest to smiling in both groups. The degree of the backward movement was significantly smaller in the older group than in the younger group (|M|, ∠N-M, P < 0.001). The value of variable ∠N-M was greater (P < 0.01) in the older group than in the younger group at the both facial postures, which means that the protrusion of the nose was smaller in the older group. The nasal width (|N-N|) showed no significant differences at rest, at the peak of smiling this value significantly increased in only the younger group, thus the younger group showed a greater nasal width at the peak of the smiling. The value of variable |P| was smaller and that of ∠E-P-N was greater in the older group than in the younger group, which suggests smaller protrusion of the cheek of the zygomatic region in the older group than in the younger group (P < 0.01; left at rest, left and right at the peak of smiling). Variable |NP|, representing the overhang of the alar lobule area, was smaller in the older group than in the younger group (left; P < 0.01) at the both facial postures; that is, the overhang of the alar lobule area was smaller in the older group. From rest to smile, the younger group showed nine variables showing significant changes whereas the older group showed only two variables showing significant changes; that is, the younger group’s smile was characterized by an increase of the nasal alar width (|N-N|) and a protrusion of the nasal wing, |NP|, P<0.001), and an increase of the cheek protrusion (∠E-P-N, ∠N-M, |P| , P<0.001).

***Sn//axial (transverse section at the base of the columella).*** The value of variable |E-E| was greater at rest in the older group than in the younger group, indicating a greater facial width at the base of the columella level (P < 0.001). From rest to smile, the value of variable |E-E| showed an increase by approximately 6 mm from rest to smiling in the older group than in the younger group, indicating a greater facial width at the base of the columella level (P < 0.001), whereas the older group showed no significant differences. The value of variables |M| showed a significant decrease in the both groups (`< 0.001), indicating retrusive movement of the nasal base in the older and younger groups when smiling, respectively. The value of variables ∠E-P-M and |P| showed significant decrease in only the younger groups (P<0.01), which may indicate a greater retrusive movement of the nasal base in the younger group.

***Ls//axial (transverse section at the upper lip vermilion).*** The value of variable |E-E| was greater in the older group than in the younger group, indicating a greater facial width at the base of the upper lip level (P < 0.01). The value of variable |P| was smaller and that of ∠E-P-M was greater in the older group than in the younger group at rest, which suggests smaller protrusion of the cheek at the level of the upper lip in the older group than in the younger group (P < 0.01). The value of variable |E-E| increased significantly in the older and younger groups from rest to smiling, indicating an increased facial width from rest to smiling at the base of the upper lip level in the both groups (P < 0.001). In contrast, the value of variable |M| showed significant decrease in only the younger group from rest to smiling, indicating that a retrusive movement of the upper lip was only observed in the younger group (P < 0.001). The value of variable |P| also showed a significant decrease and that of ∠E-P-M showed a significant increase from rest to smile in the only younger group (P < 0.01). This is related to the greater retrusive movement of the upper lip at the peak of smiling. Overall, transverse section at the upper lip vermilion (S9 Fig) showed that the older group, even at rest, showed a contour that were similar to that at the peak of smiling in the younger group, thus the older group showed no changes from rest to smile.

***Li//axial (transverse section at the lower lip vermilion).*** The value of variable |E-E| was greater in the older group than in the younger group, indicating a greater facial width at the base of the lower lip level (P < 0.001). In relation to a greater facial width in the older group, the value of variable |M| relative to the |E-E| was also smaller in the older group than in the younger group (P<0.001). The value of variable |P| was smaller and that of ∠E-P-M was greater in the older group than in the younger group at rest, which suggests smaller protrusion of the cheek at the level of the lower lip in the older group than in the younger group (P < 0.01). The value of variable |E-E| increased significantly by approximately 3 mm and 6 mm from rest to smiling in the older and younger groups, respectively, indicating an increased facial width from rest to smiling at the base of the lower lip level (P < 0.001). The value of variable |P| also significantly decreased from rest to smiling in the both group, which suggests decreased protrusion of the cheek of the parotid-masseteric region from rest to smiling in the both groups (left, P < 0.01; right P < 0.05). However, the degree of the decreased movement of the cheek of the parotid-masseteric region from rest to smile was significantly smaller in the older group than in the younger group. Furthermore, the value of variable |M| showed significant decrease by in only the younger group from rest to smiling, indicating that the retrusive movement of the lower lip was only observed in the younger group (P < 0.001). Similar to the Ls//axial, transverse section at the lower lip vermilion (S9 Fig) showed that the older group, even at rest, showed a contour that were similar to that at the peak of smiling in the younger group, thus the older group showed no changes from rest to smiling.

***Sm//axial (transverse section at the chin).*** The value of variable |E-E| was greater in the older group than in the younger group, indicating a greater facial width at the base of the chin level (P < 0.001). In the older group, there were no significant changes from rest to smiling whereas the younger group showed significantly decreased |M|, increased ∠E-P-M, and decreased |P|. These results suggest that the younger group showed retrusive movement of the chin from rest to smiling (P < 0.001).

**Facial outline (S9 Table, S7 Fig)**

The value of variables ∠Go′-Gn, and ∠Zy′-Go′-Gn was smaller in the older group than in the younger group at the both facial postures, (P < 0.01). Further, at rest, the value of variable ∠Zy′-Go′ was greater in the older group than in the younger group. These results indicate that the facial outline of the older group showed a greater sagging at the both facial postures. From rest to smile, ∠Go′-Gn, and ∠Zy′-Go′ showed a significant increase only the younger group, whereas the older group showed no significant changes from rest to smile in the facial outline.
