# Supplementary material for: Functional decline in facial expression generation in older women: A cross-sectional study using three-dimensional morphometry
Source: PLoS One. 2019 Jul 10;14(7):e0219451. doi: 10.1371/journal.pone.0219451 (PMC6636602; doi:10.1371/journal.pone.0219451)
Supplement: S1 Table — (DOCX) [file pone.0219451.s003.docx]

***S1 Table.*** *Definitions of the soft tissue landmarks on the facial 3D images.*

| Landmark |  | Definition | Single | Paired |
| --- | --- | --- | --- | --- |
| Glabella | Gla | The most prominent midpoint between the eyebrows | ✓ |  |
| Nasion | N | The deepest point on the nasofrontal region in a sagittal plane | ✓ |  |
| Exocanthion | Ex | The point at the outer commissure of the eye fissure |  | ✓ |
| Endocanthion | En | The point at the inner commissure of the eye fissure |  | ✓ |
| Palpebrale superius | Ps | The highest point on the middle portion of the free margin of each upper eyelid |  | ✓ |
| Palpebrale inferius | Pi | The lowest point on the middle portion of the free margin of each lower eyelid |  | ✓ |
| Porion | Po | The most superior point on each ear canal |  | ✓ |
| Orbitale | Or | The lowest point on the margin of the orbitale area |  | ✓ |
| Zygomaticus | Zy | The most prominent point on the zygomatic area from the 45° oblique view |  | ✓ |
| Pronasale | Prn | The most protruding point of the apex nasi in a sagittal plane | ✓ |  |
| Alar curvature point | Ac | The most posterolateral point in the curved base line of the alar, indicating the facial insertion of the base of the nasal wing base onto the curvature of the base |  | ✓ |
| Subnasale | Sn | The midpoint of the base of the columella where the lower border of the nasal septum meets the surface of the upper lip | ✓ |  |
| Labiale superius | Ls | The outermost point on the mucocutaneous border of the upper lip in the mid-sagittal plane | ✓ |  |
| Stomion | Sto | The point between the lowermost point on the vermillion of the upper lip and the uppermost point on the vermillion of the lower lip in the mid-sagittal plane | ✓ |  |
| Cheilion | Ch | The outer corner of the mouth where the outer edges of the upper and lower vermilions meet |  | ✓ |
| Labiale inferius | Li | The outermost point on the mucocutaneous border of the lower lip in the mid-sagittal plane | ✓ |  |
| Submentale | Sm | The deepest point of the midline concavity between the lower lip and chin in the mid-sagittal plane | ✓ |  |
| Pogonion | Pog | The most prominent point of the chin in the mid-sagittal plane | ✓ |  |
| Gnathion | Gn | The most anterior inferior point of the chin in the mid-sagittal plane | ✓ |  |
| Zygomaticus′ | Zy′ | The most lateral point on the facial outline (Mathematically defined; see Fig. 6) |  | ✓ |
| Gonion′ | Go′ | The most inferior and lateral point on the external angle of the mandible (Mathematically defined; see Fig. 6) |  | ✓ |
