# Supplementary material for: Functional decline in facial expression generation in older women: A cross-sectional study using three-dimensional morphometry
Source: PLoS One. 2019 Jul 10;14(7):e0219451. doi: 10.1371/journal.pone.0219451 (PMC6636602; doi:10.1371/journal.pone.0219451)
Supplement: S2 Table — (DOCX) [file pone.0219451.s004.docx]

***S2 Table.*** *Five types of curving lines.*

| **Category** | **Variable (Contour)** | **Landmark 1**  **(L_1_)** | **Landmark 2**  **(L_2_)** | **Measurement** |
| --- | --- | --- | --- | --- |
| Inter-landmark contour parallel to the Z-axis | Ex-Ac//z | Ex | Ac | 28 variables for each side. See S2 Fig |
|  | En-Ac//z | En | Ac |  |
|  | Ex-Ch//z | Ex | Ch |  |
|  | Ac-Ch//z | Ac | Ch |  |
| Sagittal section  (parallel to the Z-Y plane) | N//sagittal | N | Sn | 8 variables. See S3 Fig. |
|  | Prn//sagittal | Prn | Pog | 13 variables. See S4 Fig. |
| Axial section  (parallel to the Z-X plane) | Gla//axial | Gla | N/A | 36 variables.  See S5 Fig A. |
|  | N//axial | N | N/A |  |
|  | Sn//axial | Sn | N/A |  |
|  | Ls//axial | Ls | N/A |  |
|  | Li//axial | Li | N/A |  |
|  | Sm//axial | Sm | N/A |  |
|  | Or//axial | Or | N/A | 22 variables. See S5 Fig B. |
|  | Prn//axial | Prn | N/A |  |
| Facial outline | Facial outline | N/A | N/A | 6 variables. See S6 Fig. |
| Supraorbital ridge outline | Supraorbital ridge outline | N/A | N/A | Height-to-width ratio of the outline of the supraorbital ridge |
