# Supplementary material for: Functional decline in facial expression generation in older women: A cross-sectional study using three-dimensional morphometry
Source: PLoS One. 2019 Jul 10;14(7):e0219451. doi: 10.1371/journal.pone.0219451 (PMC6636602; doi:10.1371/journal.pone.0219451)
Supplement: S3 Table — (DOCX) [file pone.0219451.s005.docx]

***S3 Table.*** *Result summary of facial characteristics unique to the older group when compared with the younger group at rest and on smiling.*

|  | **Facial characteristics** | **Findings unique to the older group when compared with the younger group** | | |
| --- | --- | --- | --- | --- |
|  |  | **Rest posture** | **Smile posture** | **Changes from rest to smile** |
| **Facial outline** | Facial width | Greater* | NS | Smaller increase |
|  | Vertical position of the gonial angle of the facial outline | NS | Lower* | Smaller increase |
|  | Antero-posterior position of the facial outline | More protruded* | NS | Smaller change |
| **Eyes** | Distances between the eyes | Smaller | Smaller | NS |
|  | Height of the eye fissures | Smaller | Smaller* | Greater decrease |
|  | Vertical position of eyes | Lower | Lower | Smaller upward movement |
| **Cheek** | Vertical position of cheek | Lower | Lower | Smaller downward movement |
|  | Sagging of skin in the orbital and infraorbital regions | Greater* | Greater* | Smaller increase |
|  | Flabbiness of the cheeks at the level of the lips and the chin | More flabby* | NS | NS |
| **Nose** | Nasal width | Greater | Greater | Smaller increase |
|  | Width of the nasal dorsum at the orbital level | NS | Greater | NS |
|  | Vertical position of nose | Lower | Lower | Smaller upward movement |
|  | Antero-posterior position of the nasal ala | More protruded* | NS | Smaller backward movement |
|  | Antero-posterior position of the nasal tip | NS | NS | Smaller backward movement |
|  | Nasal tip shape | Rounder | Rounder | NS |
|  | Overhang of the alar lobule area | Smaller | Smaller | NS |
| **Subnasal region** | Vertical length of subnasal region | Greater* | Greater | NS |
|  | Antero-posterior protrusion of the subnasal region | Greater | Greater | NS |
|  | Shape of the subnasal region | More convex* | More convex* | NS |
| **Mouth** | Mouth width | Smaller | Smaller | Smaller lateral movement |
|  | Vertical position of corner of the mouth | Lower | Lower* | Smaller upward movement |
|  | Antero-posterior position of the lips | NS | More protruded* | Smaller retrusive movement |
|  | Antero-posterior position of corner of the mouth | More retruded | Less retruded | Smaller retrusive movement |
| **Chin** | Chin width | Greater | Greater | Smaller increase |
|  | Lower facial height | Greater | Greater | NS |
|  | Depth of the labio-mental fold | NS | Deeper | Smaller decrease |

*, predictor variables selected in a stepwise discriminant function analysis; NS, not significant.
