# Supplementary material for: Functional decline in facial expression generation in older women: A cross-sectional study using three-dimensional morphometry
Source: PLoS One. 2019 Jul 10;14(7):e0219451. doi: 10.1371/journal.pone.0219451 (PMC6636602; doi:10.1371/journal.pone.0219451)
Supplement: S4 Table — (DOCX) [file pone.0219451.s006.docx]

***S4 Table.*** *Means and their standard deviations (S.D.) of the 29 inter-landmark distances that had been reported in previous studies and the height-to-width ratio of the outline of the supraorbital ridge for each group.*

|  |  | **Rest** | | | | | | **Smile** | | | | | | | **P-value (Rest vs. Smile)** | | | |
| --- | --- | --- | --- | --- | --- | --- | --- | --- | --- | --- | --- | --- | --- | --- | --- | --- | --- | --- |
| **Variable** | | **Older** | | **Younger** | | **P-value**  **(Older vs. Younger)** | | **Older** | | **Younger** | | **P-value**  **(Older vs. Younger)** | | | **Older** | | **Younger** | |
|  |  | **Mean** | **S.D.** | **Mean** | **S.D.** |  |  | **Mean** | **S.D.** | **Mean** | **S.D.** |  |  |  |  |  |  |  |
| X-value | \|Ex-Ex\| (mm) | 87.8 | 3.3 | 91.7 | 4.0 | 3E-06 | ** | 87.9 | 3.4 | 92.6 | 4.2 | | 1E-07 | ** | 0.788 |  | 6E-04 | ** |
|  | \|Ac-Ac\| (mm) ^a)^ | 39.0 | 2.9 | 36.8 | 2.0 | 6E-06 | ** | 41.1 | 3.5 | 40.9 | 2.7 | | 0.717 |  | 7E-07 | ** | 7E-32 | ** |
|  | \|Zy-Zy\| (mm) ^a)^ | 113.0 | 5.4 | 111.9 | 4.8 | 0.295 |  | 113.3 | 6.0 | 111.2 | 5.1 | | 0.067 |  | 0.826 |  | 2E-04 | ** |
|  | \|Zy′-Zy′\| (mm) ^a)^ | 128.4 | 6.0 | 129.1 | 5.2 | 0.49 |  | 128.5 | 5.9 | 128.8 | 5.0 | | 0.835 |  | 0.955 |  | 4E-04 | ** |
|  | \|Go′-Go′\| (mm) ^a)^ | 103.6 | 6.6 | 98.4 | 6.6 | 3E-04 | ** | 105.8 | 7.4 | 106.7 | 6.7 | | 0.543 |  | 0.009 | * | 9E-21 | ** |
|  | \|Ch-Ch\| (mm) ^a)^ | 49.5 | 3.5 | 46.5 | 4.0 | 4E-04 | ** | 53.3 | 4.9 | 58.3 | 5.5 | | 1E-05 | ** | 7E-05 | ** | 8E-37 | * * |
| Y-value | \|Gla-N\| (mm) ^a)^ | 22.0 | 6.2 | 23.5 | 6.0 | 0.212 |  | 22.5 | 6.1 | 23.3 | 5.6 | | 0.493 |  | 0.158 |  | 0.190 |  |
|  | \|Gla-En\| (mm) ^a)^ | 28.4 | 6.4 | 28.7 | 6.1 | 0.826 |  | 28.1 | 6.8 | 28.0 | 5.6 | | 0.913 |  | 0.526 |  | 0.006 | * |
|  | \|Gla-Sn\| (mm) Total midface height ^a)^ | 73.9 | 7.1 | 73.3 | 6.2 | 0.676 |  | 73.2 | 7.1 | 72.8 | 5.8 | | 0.753 |  | 0.229 |  | 0.006 | * |
|  | \|Gla-Zy\| (mm)^a)^ | 52.3 | 7.1 | 51.3 | 6.3 | 0.45 |  | 53.9 | 8.1 | 55.3 | 6.7 | | 0.356 |  | 0.083 |  | 5E-09 | ** |
|  | \|Gla-Prn\| (mm)^a)^ | 63.4 | 6.8 | 62.0 | 6.5 | 0.301 |  | 62.8 | 7.1 | 61.3 | 6.1 | | 0.254 |  | 0.259 |  | 2E-04 | ** |
|  | \|Gla-Ls\| (mm)^a)^ | 88.9 | 8.0 | 84.7 | 6.9 | 0.005 | * | 86.0 | 8.3 | 80.9 | 6.5 | | 7E-04 | ** | 0.001 | ** | 8E-16 | ** |
|  | \|Gla-Sto\| (mm) Total upper face height ^a)^ | 96.3 | 7.7 | 92.2 | 6.6 | 0.005 | * | 92.7 | 8.5 | 87.4 | 6.4 | | 4E-04 | ** | 5E-04 | ** | 1E-19 | ** |
|  | \|Gla-Gn\| (mm) Total face height ^a)^ | 138.5 | 9.1 | 135.7 | 7.0 | 0.07 |  | 138.6 | 9.2 | 136.2 | 6.6 | | 0.112 |  | 0.947 |  | 0.178 |  |
|  | \|N-En\| (mm)^a)^ | 6.4 | 1.9 | 5.1 | 1.4 | 1E-04 | ** | 5.6 | 1.7 | 4.7 | 1.4 | | 0.003 | * | 0.038 |  | 0.003 | * |
|  | \|N-Sn\| (mm) Midface height ^a)^ | 51.9 | 3.5 | 49.8 | 2.8 | 6E-04 | ** | 50.7 | 3.4 | 49.5 | 2.8 | | 0.05 |  | 0.01 | * | 0.003 | * |
|  | \|N-Zy\| (mm) ^a)^ | 30.3 | 5.0 | 27.7 | 4.6 | 0.009 | * | 31.4 | 6.3 | 31.9 | 4.9 | | 0.626 |  | 0.206 |  | 7E-12 | ** |
|  | \|N-Prn\| (mm) Nasal bridge length ^a)^ | 41.5 | 3.1 | 38.5 | 2.9 | 3E-06 | ** | 40.3 | 3.3 | 38.0 | 3.0 | | 4E-04 | ** | 0.015 |  | 6E-04 | ** |
|  | \|N-Ls\| (mm) ^a)^ | 67.0 | 5.2 | 61.2 | 3.8 | 5E-10 | ** | 63.5 | 5.3 | 57.6 | 3.5 | | 6E-11 | ** | 2E-05 | ** | 7E-23 | ** |
|  | \|N-Sto\| (mm)Upper face height ^a)^ | 74.3 | 5.0 | 68.7 | 3.7 | 7E-10 | ** | 70.2 | 5.5 | 64.1 | 3.6 | | 4E-11 | ** | 2E-05 | ** | 6E-28 | ** |
|  | \|N-Gn\| (mm) Face height ^a)^ | 116.5 | 6.7 | 112.1 | 5.6 | 5E-04 | ** | 116.1 | 6.3 | 112.8 | 5.2 | | 0.005 | * | 0.443 |  | 0.352 |  |
|  | \|Go′-Gn\| (mm) Chin height ^d)^ | 24.8 | 3.4 | 25.4 | 3.9 | 0.398 |  | 26.5 | 4.0 | 31.4 | 4.6 | | 7E-07 | ** | 0.004 | * | 1E-19 | ** |
|  | \|Ps-Pi\| (mm) Eye height ^a)^ | 11.8 | 1.5 | 13.5 | 1.5 | 2E-07 | ** | 11.4 | 1.4 | 13.7 | 1.5 | | 2E-11 | ** | 0.148 |  | 0.320 |  |
|  | \|Sn-Sto\| (mm) Maxilla height ^a)^ | 22.4 | 2.5 | 18.9 | 2.3 | 7E-11 | ** | 19.5 | 3.3 | 14.6 | 2.6 | | 2E-14 | ** | 2E-06 | ** | 5E-39 | ** |
|  | \|Sn-Gn\| (mm) Lower face height ^a)^ | 63.8 | 4.4 | 60.2 | 4.7 | 3E-04 | ** | 59.7 | 5.4 | 56.5 | 4.9 | | 0.003 | * | 1E-05 | ** | 1E-15 | ** |
|  | \|Ls-Sto\| (mm) Upper lip vermillion height ^a)^ | 7.4 | 1.4 | 7.5 | 1.5 | 0.582 |  | 6.7 | 1.3 | 6.5 | 1.6 | | 0.447 |  | 0.025 |  | 9E-10 | ** |
|  | \|Sto-Li\| (mm) Lower lip vermillion height ^a)^ | 8.7 | 1.9 | 9.2 | 1.7 | 0.18 |  | 7.2 | 1.5 | 7.6 | 1.8 | | 0.326 |  | 1E-05 | ** | 8E-10 | ** |
|  | \|Sto-Gn\| (mm) Mandible height ^a)^ | 41.4 | 3.7 | 41.3 | 4.9 | 0.949 |  | 40.2 | 4.6 | 41.9 | 4.6 | | 0.074 |  | 0.017 |  | 0.634 |  |
| Z-value | \|Gla-N\| (mm) ^a)^ | 3.6 | 2.4 | 4.1 | 2.1 | 0.256 |  | 3.2 | 2.2 | 3.7 | 1.9 | | 0.222 |  | 0.936 |  | 0.106 |  |

* P < 0.01; ** P < 0.001; a) Farkas and Munro. (1987); b) Sarver (1998); c) Carre et al., 2009; d) Newly defined.

***S4 Table Contd.*** *Means and their standard deviations (S.D.) of the 29 inter-landmark distances that had been reported in previous studies and the height-to-width ratio of the outline of the supraorbital ridge for each group.*

|  |  | **Smile-Rest** | | | | | |
| --- | --- | --- | --- | --- | --- | --- | --- |
| **Variable** | | **Older** | | **Younger** | | **P-value**  **(Older vs. Younger)** | |
|  |  | **Mean** | **S.D.** | **Mean** | **S.D.** |  |  |
| X-value | \|Ex-Ex\| (mm) | 0.1 | 2.4 | 0.9 | 2.7 | 0.129 |  |
|  | \|Ac-Ac\| (mm) ^a)^ | 2.3 | 2.0 | 4.0 | 2.3 | 3E-04 | ** |
|  | \|Zy-Zy\| (mm) ^a)^ | 0.2 | 4.0 | -2.0 | 5.3 | 0.04 |  |
|  | \|Zy′-Zy′\| (mm) ^a)^ | 0.0 | 3.8 | -1.8 | 4.9 | 0.059 |  |
|  | \|Go′-Go′\| (mm) ^a)^ | 2.5 | 4.8 | 7.9 | 6.6 | 6E-05 | ** |
|  | \|Ch-Ch\| (mm) ^a)^ | 4.2 | 5.0 | 12.3 | 6.1 | 1E-09 | ** |
| Y-value | \|Gla-N\| (mm) ^a)^ | 0.6 | 2.4 | -0.5 | 3.4 | 0.109 |  |
|  | \|Gla-En\| (mm) ^a)^ | -0.3 | 2.6 | -1.0 | 3.5 | 0.316 |  |
|  | \|Gla-Sn\| (mm) Total midface height ^a)^ | -0.8 | 3.5 | -1.3 | 4.5 | 0.586 |  |
|  | \|Gla-Zy\| (mm)^a)^ | 1.8 | 5.6 | 3.8 | 5.9 | 0.107 |  |
|  | \|Gla-Prn\| (mm)^a)^ | -0.7 | 3.6 | -1.4 | 3.7 | 0.387 |  |
|  | \|Gla-Ls\| (mm)^a)^ | -3.4 | 5.2 | -5.0 | 5.2 | 0.148 |  |
|  | \|Gla-Sto\| (mm) Total upper face height ^a)^ | -4.1 | 5.7 | -6.2 | 5.4 | 0.073 |  |
|  | \|Gla-Gn\| (mm) Total face height ^a)^ | -0.1 | 5.0 | -0.9 | 6.4 | 0.525 |  |
|  | \|N-En\| (mm)^a)^ | -0.9 | 2.3 | -0.6 | 1.8 | 0.348 |  |
|  | \|N-Sn\| (mm) Midface height ^a)^ | -1.4 | 2.8 | -0.8 | 2.7 | 0.304 |  |
|  | \|N-Zy\| (mm) ^a)^ | 1.2 | 5.1 | 4.2 | 5.5 | 0.007 | * |
|  | \|N-Prn\| (mm) Nasal bridge length ^a)^ | -1.4 | 2.9 | -1.0 | 2.7 | 0.465 |  |
|  | \|N-Ls\| (mm) ^a)^ | -4.0 | 4.4 | -4.5 | 3.5 | 0.524 |  |
|  | \|N-Sto\| (mm)Upper face height ^a)^ | -4.7 | 5.0 | -5.7 | 3.7 | 0.245 |  |
|  | \|N-Gn\| (mm) Face height ^a)^ | -0.6 | 4.6 | -0.5 | 5.3 | 0.889 |  |
|  | \|Go′-Gn\| (mm) Chin height ^d)^ | 1.9 | 3.3 | 6.1 | 5.4 | 8E-05 | ** |
|  | \|Ps-Pi\| (mm) Eye height ^a)^ | -0.4 | 1.6 | 0.0 | 0.0 | 0.007 | * |
|  | \|Sn-Sto\| (mm) Maxilla height ^a)^ | -3.3 | 3.0 | -4.9 | 2.3 | 0.003 | * |
|  | \|Sn-Gn\| (mm) Lower face height ^a)^ | -4.7 | 5.0 | -4.7 | 4.9 | 0.976 |  |
|  | \|Ls-Sto\| (mm) Upper lip vermillion height ^a)^ | -0.7 | 1.6 | -1.2 | 1.7 | 0.173 |  |
|  | \|Sto-Li\| (mm) Lower lip vermillion height ^a)^ | -1.7 | 1.8 | -1.8 | 2.7 | 0.8 |  |
|  | \|Sto-Gn\| (mm) Mandible height ^a)^ | -1.4 | 3.0 | 0.2 | 4.4 | 0.065 |  |
| Z-value | \|Gla-N\| (mm) ^a)^ | 0.0 | 0.4 | -0.1 | 0.8 | 0.372 |  |

* P < 0.01; ** P < 0.001; a) Farkas and Munro. (1987); b) Sarver (1998); c) Carre et al., 2009; d) Newly defined
