# Supplementary material for: Functional decline in facial expression generation in older women: A cross-sectional study using three-dimensional morphometry
Source: PLoS One. 2019 Jul 10;14(7):e0219451. doi: 10.1371/journal.pone.0219451 (PMC6636602; doi:10.1371/journal.pone.0219451)
Supplement: S5 Table — (DOCX) [file pone.0219451.s007.docx]

***S5 Table.*** *Means and their standard deviations (S.D.) of the 15 ratios that had been reported in previous studies and the height-to-width ratio of the outline of the supraorbital ridge for each group.*

| **Variable** | | **Rest** | | | | | | **Smile** | | | | | | **P-value (Rest vs. Smile)** | | | |
| --- | --- | --- | --- | --- | --- | --- | --- | --- | --- | --- | --- | --- | --- | --- | --- | --- | --- |
|  |  | **Older** | | **Younger** | | **P-value (Older vs. Younger)** | | **Older** | | **Younger** | | **P-value (Older vs. Younger)** | | **Older** | | **Younger** | |
|  |  | **Mean** | **S.D.** | **Mean** | **S.D.** |  |  | **Mean** | **S.D.** | **Mean** | **S.D.** |  |  |  |  |  |  |
| Height-to-height ratio | \|Gla-Sn\|/\|Sn-Gn\| Total midface-lower face height index^-a)^ | 1.16 | 0.12 | 1.23 | 0.16 | 0.04 |  | 1.24 | 0.15 | 1.30 | 0.16 | 0.05 |  | 7E-06 | ** | 1E-09 | ** |
|  | \|N-Sn\|/\|Sn-Gn\| Midface-lower face height index ^a)^ | 0.82 | 0.06 | 0.83 | 0.08 | 0.328 |  | 0.86 | 0.09 | 0.88 | 0.09 | 0.178 |  | 9E-04 | ** | 7E-11 | ** |
|  | \|Sn-Gn\|/\|N-Gn\| Lower face-face height index ^a)^ | 0.55 | 0.02 | 0.54 | 0.02 | 0.026 |  | 0.51 | 0.04 | 0.50 | 0.03 | 0.063 |  | 4E-06 | ** | 4E-21 | ** |
|  | \|Sto-Gn\|/\|N-Sto\| Mandible-upper face height index ^a)^ | 0.56 | 0.06 | 0.6 | 0.09 | 0.008 | * | 0.58 | 0.09 | 0.66 | 0.08 | 1E-05 | ** | 0.037 |  | 1E-12 | ** |
|  | \|Sto-Gn\|/\|Sn-Gn\| Mandible-lower face height index ^a, b)^ | 0.65 | 0.03 | 0.68 | 0.04 | 2E-05 | ** | 0.67 | 0.05 | 0.74 | 0.04 | 5E-12 | ** | 7E-05 | ** | 3E-33 | ** |
|  | \|Sm-Gn\|/\|Sn-Gn\| Chin-lower face height index ^a)^ | 0.4 | 0.05 | 0.43 | 0.06 | 0.002 | * | 0.43 | 0.06 | 0.48 | 0.06 | 6E-05 | ** | 2E-07 | ** | 3E-14 | ** |
| Height-to-width ratio | \|N-Gn\|/\|Zy′-Zy′\| Facial index ^a)^ | 0.91 | 0.06 | 0.87 | 0.05 | 5E-04 | ** | 0.90 | 0.05 | 0.88 | 0.05 | 0.008 | * | 0.315 |  | 0.003 | * |
|  | \|N-Sto\|/\|Zy′-Zy′\| Upper face index ^a) c)^ | 0.69 | 0.06 | 0.66 | 0.05 | 0.002 | * | 0.67 | 0.07 | 0.63 | 0.05 | 7E-04 | ** | 1E-04 | ** | 4E-18 | ** |
|  | \|Gla-Ls\|/\|Zy′-Zy′\| ^a)^ | 0.58 | 0.04 | 0.53 | 0.03 | 1E-09 | ** | 0.55 | 0.05 | 0.5 | 0.03 | 3E-10 | ** | 1E-06 | ** | 8E-31 | ** |
|  | \|Gla-Sto\|/\|Zy′-Zy′\| ^a)^ | 0.75 | 0.06 | 0.71 | 0.05 | 0.002 | * | 0.72 | 0.07 | 0.68 | 0.05 | 4E-04 | ** | 3E-05 | ** | 3E-24 | ** |
|  | \|N-Gn\|/\|Go′-Go′\| Face height-mandible width index ^a)^ | 1.13 | 0.09 | 1.14 | 0.09 | 0.452 |  | 1.10 | 0.09 | 1.06 | 0.07 | 0.014 |  | 0.008 | * | 2E-22 | ** |
|  | \|Sto-Gn\|/\|Go′-Go′\| Mandibular index ^a)^ | 0.40 | 0.04 | 0.42 | 0.05 | 0.042 |  | 0.38 | 0.05 | 0.39 | 0.04 | 0.176 |  | 5E-04 | ** | 4E-12 | ** |
|  | Height-to-width ratio of the outline of the supraorbital ridge ^d)^ | 0.52 | 0.23 | 0.62 | 0.24 | 0.063 |  | 0.54 | 0.25 | 0.62 | 0.23 | 0.098 |  | 0.283 |  | 0.611 |  |
|  | \|Ps-Pi\|/\|Ex-En\| Height-to-width ratio of the eye ^b)^ | 0.44 | 0.05 | 0.5 | 0.05 | 4E-07 | ** | 0.43 | 0.06 | 0.50 | 0.05 | 4E-09 | ** | 0.213 |  | 0.320 |  |
| Width-to-width ratio | \|Ch-Ch\|/\|Zy′-Zy′\| Mouth-face index ^a)^ | 0.39 | 0.03 | 0.36 | 0.03 | 3E-04 | ** | 0.41 | 0.04 | 0.45 | 0.04 | 3E-05 | ** | 4E-05 | ** | 1E-38 | ** |
|  | \|Go′-Go′\|/\|Zy′-Zy′\| ^a)^ | 0.81 | 0.05 | 0.76 | 0.04 | 2E-06 | ** | 0.82 | 0.05 | 0.83 | 0.04 | 0.542 |  | 0.001 | ** | 2E-28 | ** |

* P < 0.01; ** P < 0.001; a) Farkas and Munro. (1987); b) Sarver (1998); c) Carre et al., 2009; d) Newly defined

***S5 Table Contd.*** *Means and their standard deviations (S.D.) of the 15 ratios that had been reported in previous studies and the height-to-width ratio of the outline of the supraorbital ridge for each group.*

| **Variable** | | **Smile - Rest** | | | | | |
| --- | --- | --- | --- | --- | --- | --- | --- |
|  |  | **Older** | | **Younger** | | **P-value (Older vs. Younger)** | |
|  |  | **Mean** | **S.D.** | **Mean** | **S.D.** |  |  |
| Height-to-height ratio | \|Gla-Sn\|/\|Sn-Gn\| Total midface-lower face height index^-a)^ | 0.07 | 0.07 | 0.07 | 0.11 | 0.978 |  |
|  | \|N-Sn\|/\|Sn-Gn\| Midface-lower face height index ^a)^ | 0.04 | 0.06 | 0.05 | 0.07 | 0.437 |  |
|  | \|Sn-Gn\|/\|N-Gn\| Lower face-face height index ^a)^ | -0.03 | 0.03 | -0.04 | 0.03 | 0.651 |  |
|  | \|Sto-Gn\|/\|N-Sto\| Mandible-upper face height index ^a)^ | 0.02 | 0.04 | 0.05 | 0.06 | 0.006 | * |
|  | \|Sto-Gn\|/\|Sn-Gn\| Mandible-lower face height index ^a, b)^ | 0.03 | 0.03 | 0.06 | 0.03 | 3E-06 | ** |
|  | \|Sm-Gn\|/\|Sn-Gn\| Chin-lower face height index ^a)^ | 0.03 | 0.03 | 0.04 | 0.05 | 0.179 |  |
| Height-to-width ratio | \|N-Gn\|/\|Zy′-Zy′\| Facial index ^a)^ | 0.00 | 0.03 | 0.01 | 0.03 | 0.021 |  |
|  | \|N-Sto\|/\|Zy′-Zy′\| Upper face index ^a) c)^ | -0.02 | 0.03 | -0.03 | 0.03 | 0.464 |  |
|  | \|Gla-Ls\|/\|Zy′-Zy′\| ^a)^ | -0.03 | 0.03 | -0.03 | 0.02 | 0.727 |  |
|  | \|Gla-Sto\|/\|Zy′-Zy′\| ^a)^ | -0.03 | 0.03 | -0.04 | 0.03 | 0.218 |  |
|  | \|N-Gn\|/\|Go′-Go′\| Face height-mandible width index ^a)^ | -0.03 | 0.05 | -0.08 | 0.06 | 5E-05 | ** |
|  | \|Sto-Gn\|/\|Go′-Go′\| Mandibular index ^a)^ | -0.02 | 0.03 | -0.03 | 0.03 | 0.301 |  |
|  | Height-to-width ratio of the outline of the supraorbital ridge ^d)^ | 0.02 | 0.10 | 0.01 | 0.15 | 0.688 |  |
|  | \|Ps-Pi\|/\|Ex-En\| Height-to-width ratio of the eye ^b)^ | -0.01 | 0.05 | 0.00 | 0.00 | 0.019 |  |
| Width-to-width ratio | \|Ch-Ch\|/\|Zy′-Zy′\| Mouth-face index ^a)^ | 0.03 | 0.03 | 0.09 | 0.04 | 2E-11 | ** |
|  | \|Go′-Go′\|/\|Zy′-Zy′\| ^a)^ | 0.02 | 0.03 | 0.07 | 0.04 | 1E-08 | ** |

* P < 0.01; ** P < 0.001; a) Farkas and Munro. (1987); b) Sarver (1998); c) Carre et al., 2009; d) Newly defined. For definition of the variables, please see http://dx.doi.org/10.17632/wby6gkyfft.1
