# Supplementary material for: Functional decline in facial expression generation in older women: A cross-sectional study using three-dimensional morphometry
Source: PLoS One. 2019 Jul 10;14(7):e0219451. doi: 10.1371/journal.pone.0219451 (PMC6636602; doi:10.1371/journal.pone.0219451)
Supplement: S6 Table — (DOCX) [file pone.0219451.s008.docx]

***S6 Table.*** *Means and their standard deviations (S.D.) for the 28 variables for the contours Ex-Ac//z, En-Ac//z, Ex-Ch//z, and Ac-Ch//z for each side.*

| **Contour** | | **Variable** | **Rest** | | | | | | **Smile** | | | | | | **P-value (Rest vs. Smile)** | | | |
| --- | --- | --- | --- | --- | --- | --- | --- | --- | --- | --- | --- | --- | --- | --- | --- | --- | --- | --- |
|  |  |  | **Older** | | **Younger** | | **P-value** | | **Older** | | **Younger** | | **P-value** | | **Older** | | **Younger** | |
|  |  |  | **Mean** | **S.D.** | **Mean** | **S.D.** |  |  | **Mean** | **S.D.** | **Mean** | **S.D.** |  |  |  |  |  |  |
| Left | Ex-Ac//z | ∠L_1_-L_2_ (°) | 8.7 | 3.6 | 11.4 | 4.1 | 0.002 | * | 8.8 | 3.9 | 11.0 | 3.9 | 0.008 | * | 0.724 |  | 0.196 |  |
|  |  | \|L_1_-L_2_\| (%) | 53.1 | 3.0 | 52 | 2.8 | 0.056 |  | 51.4 | 4.1 | 49.5 | 2.9 | 0.005 | * | 0.001 | ** | 6E-15 | ** |
|  |  | \|P\| (% to \|L_1_-L_2_\|) | 7.0 | 3.1 | 7.3 | 2.2 | 0.562 |  | 8.4 | 3.0 | 8.8 | 2.5 | 0.404 |  | 0.001 | ** | 9E-08 | ** |
|  |  | \|L_1_-P\| (% to \|L_1_-L_2_\|) | 34.2 | 9.8 | 31.1 | 12.4 | 0.221 |  | 32.4 | 12.3 | 32.7 | 15.4 | 0.924 |  | 0.446 |  | 0.29 |  |
|  |  | ∫ (L_1_-L_2_) (% to \|L_1_-L_2_\|) | 4.5 | 1.7 | 4.4 | 1.5 | 0.842 |  | 5.7 | 2.0 | 6.0 | 1.7 | 0.448 |  | 5E-04 | ** | 5E-14 | ** |
|  |  | ∠L_1_-P (°) | 11.8 | 5.7 | 14.3 | 5.0 | 0.02 |  | 15.1 | 6.3 | 16.5 | 5.6 | 0.248 |  | 3E-04 | ** | 2E-04 | ** |
|  |  | \|L_1_-L_2_\|/L_1_^L_2_ (%) | 96.3 | 1.7 | 97.3 | 1.4 | 0.001 | * | 95.5 | 1.9 | 95.7 | 1.6 | 0.69 |  | 0.031 |  | 3E-16 | ** |
|  | En-Ac//z | ∠L_1_-L_2_ (°) | 4.9 | 3.4 | 4.5 | 3.9 | 0.559 |  | 4.7 | 3.7 | 3.1 | 3.8 | 0.053 |  | 0.413 |  | 7E-08 | ** |
|  |  | \|L_1_-L_2_\| (%) | 42.8 | 3.4 | 38.9 | 3.7 | 9E-07 | ** | 41.8 | 4.5 | 37 | 3.7 | 2E-08 | ** | 0.095 |  | 7E-10 | ** |
|  |  | \|P\| (% to \|L_1_-L_2_\|) | 8.5 | 4.1 | 4.6 | 3.2 | 1E-07 | ** | 8.6 | 4.7 | 5.2 | 4.3 | 3E-04 | ** | 0.936 |  | 0.137 |  |
|  |  | \|L_1_-P\| (% to \|L_1_-L_2_\|) | 51.9 | 19.3 | 45.0 | 26 | 0.183 |  | 50.1 | 22.7 | 45.1 | 28.1 | 0.375 |  | 0.603 |  | 0.987 |  |
|  |  | ∫ (L_1_-L_2_) (% to \|L_1_-L_2_\|) | 5.3 | 1.6 | 3.0 | 1.4 | 5E-12 | ** | 5.6 | 1.6 | 3.3 | 1.6 | 4E-10 | ** | 0.319 |  | 0.012 |  |
|  |  | ∠L_1_-P (°) | 8.8 | 3.0 | 5.9 | 5.4 | 0.005 | * | 9.3 | 2.7 | 5.4 | 4.4 | 1E-05 | ** | 0.399 |  | 0.515 |  |
|  |  | \|L_1_-L_2_\|/L_1_^L_2_ (%) | 97.5 | 1.1 | 98.7 | 0.9 | 3E-08 | ** | 97.1 | 1.4 | 98.2 | 1.2 | 3E-05 | ** | 0.032 |  | 1E-04 | ** |
|  | Ex-Ch//z | ∠L_1_-L_2_ (°) | 0.2 | 3.8 | 4.8 | 4.0 | 1E-07 | ** | -2.7 | 4.0 | -2.9 | 4.5 | 0.823 |  | 4E-06 | ** | 3E-37 | ** |
|  |  | \|L_1_-L_2_\| (%) | 82.9 | 5.7 | 78.7 | 4.5 | 5E-05 | ** | 79 | 5.6 | 70.1 | 4.5 | 3E-15 | ** | 2E-05 | ** | 1E-38 | ** |
|  |  | \|P\| (% to \|L_1_-L_2_\|) | 9.7 | 4.7 | 7.8 | 3.4 | 0.013 |  | 14.7 | 5.6 | 18.6 | 4.4 | 1E-04 | ** | 1E-06 | ** | 5E-45 | ** |
|  |  | \|L_1_-P\| (% to \|L_1_-L_2_\|) | 48.6 | 17.4 | 42.2 | 17.1 | 0.076 |  | 49.8 | 12.1 | 53.5 | 8.1 | 0.054 |  | 0.768 |  | 7E-09 | ** |
|  |  | ∫ (L_1_-L_2_) (% to \|L_1_-L_2_\|) | 6.9 | 2.0 | 5.7 | 2.0 | 0.005 | * | 9.4 | 2.4 | 11.4 | 2.4 | 1E-04 | ** | 4E-08 | ** | 6E-41 | ** |
|  |  | ∠L_1_-P (°) | 10.4 | 8.0 | 11.1 | 5.1 | 0.547 |  | 15.9 | 4.9 | 19.4 | 4.6 | 4E-04 | ** | 1E-05 | ** | 6E-20 | ** |
|  |  | \|L_1_-L_2_\|/L_1_^L_2_ (%) | 95.5 | 1.8 | 96.1 | 1.5 | 0.044 |  | 92.3 | 2.3 | 91 | 2.7 | 0.02 |  | 6E-10 | ** | 3E-34 | ** |
|  | Ac-Ch//z | ∠L_1_-L_2_ (°) | -12.1 | 5.4 | -6.3 | 5.9 | 4E-06 | ** | -18.9 | 6.6 | -23.8 | 6.6 | 6E-04 | ** | 1E-07 | ** | 1E-49 | ** |
|  |  | \|L_1_-L_2_\| (%) | 36.9 | 3.3 | 33.6 | 3.0 | 2E-06 | ** | 36.1 | 3.1 | 32.2 | 2.9 | 5E-09 | ** | 0.217 |  | 2E-05 | ** |
|  |  | \|P\| (% to \|L_1_-L_2_\|) | 16.2 | 5.7 | 13.3 | 7.7 | 0.059 |  | 16.7 | 4.5 | 13.9 | 4.7 | 0.006 | * | 0.687 |  | 0.387 |  |
|  |  | \|L_1_-P\| (% to \|L_1_-L_2_\|) | 61.8 | 16.2 | 54.2 | 27.5 | 0.152 |  | 63.7 | 12.6 | 65 | 9.2 | 0.553 |  | 0.591 |  | 4E-04 | ** |
|  |  | ∫ (L_1_-L_2_) (% to \|L_1_-L_2_\|) | 10.0 | 2.5 | 8.9 | 2.6 | 0.04 |  | 9.9 | 2.7 | 8.1 | 3.0 | 0.004 | * | 0.866 |  | 0.008 | * |
|  |  | ∠L_1_-P (°) | 14.0 | 4.6 | 12.0 | 6.1 | 0.115 |  | 15.1 | 4 | 12.1 | 4.1 | 4E-04 | ** | 0.228 |  | 0.954 |  |
|  |  | \|L_1_-L_2_\|/L_1_^L_2_ (%) | 91.1 | 2.7 | 91.9 | 2.6 | 0.179 |  | 91.1 | 2.5 | 93.5 | 3.3 | 3E-04 | ** | 0.938 |  | 3E-06 | ** |
| Right | Ex-Ac//z | ∠L_1_-L_2_ (°) | 8.7 | 3.8 | 11.5 | 3.4 | 3E-04 | ** | 9 | 3.9 | 10.5 | 3.7 | 0.062 |  | 0.457 |  | 5E-04 | ** |
|  |  | \|L_1_-L_2_\| (%) | 54.2 | 4.2 | 53.2 | 2.7 | 0.128 |  | 52.6 | 4.5 | 49.9 | 3.1 | 4E-04 | ** | 0.002 | * | 4E-25 | ** |
|  |  | \|P\| (% to \|L_1_-L_2_\|) | 6.2 | 3.4 | 6.4 | 2.2 | 0.678 |  | 7.5 | 2.9 | 8 | 2.5 | 0.31 |  | 0.003 | * | 4E-08 | ** |
|  |  | \|L_1_-P\| (% to \|L_1_-L_2_\|) | 36.9 | 12.6 | 32.4 | 10.2 | 0.048 |  | 35.7 | 12.3 | 37.1 | 17.1 | 0.669 |  | 0.089 |  | 0.003 | * |
|  |  | ∫ (L_1_-L_2_) (% to \|L_1_-L_2_\|) | 4.2 | 1.5 | 3.8 | 1.5 | 0.21 |  | 5.2 | 1.5 | 5.2 | 1.8 | 0.955 |  | 5E-04 | ** | 2E-12 | ** |
|  |  | ∠L_1_-P (°) | 9.8 | 6.2 | 11.6 | 4.4 | 0.079 |  | 12.3 | 5.1 | 13.5 | 5.2 | 0.269 |  | 0.001 | ** | 4E-04 | ** |
|  |  | \|L_1_-L_2_\|/L_1_^L_2_ (%) | 96.0 | 1.3 | 97.6 | 0.9 | 4E-11 | ** | 95.3 | 1.7 | 96 | 1.5 | 0.032 |  | 0.025 | * | 3E-18 | ** |
|  | En-Ac//z | ∠L_1_-L_2_ (°) | 5.3 | 3.7 | 5.5 | 3.9 | 0.821 |  | 5.1 | 3.8 | 4.2 | 3.9 | 0.234 |  | 0.476 |  | 5E-08 | ** |
|  |  | \|L_1_-L_2_\| (%) | 42.5 | 4 | 38.4 | 3.4 | 1E-07 | ** | 41.4 | 4.6 | 36.4 | 3.8 | 2E-08 | ** | 0.071 |  | 6E-10 | ** |
|  |  | \|P\| (% to \|L_1_-L_2_\|) | 8.5 | 3.6 | 4.9 | 3.3 | 1E-06 | ** | 8.7 | 4.2 | 4.8 | 4.0 | 9E-06 | ** | 0.806 |  | 0.636 |  |
|  |  | \|L_1_-P\| (% to \|L_1_-L_2_\|) | 52.1 | 18.9 | 40.8 | 25.6 | 0.027 |  | 51.6 | 21.1 | 38.9 | 27.8 | 0.023 |  | 0.879 |  | 0.51 |  |
|  |  | ∫ (L_1_-L_2_) (% to \|L_1_-L_2_\|) | 5.2 | 1.5 | 3.2 | 1.6 | 2E-08 | ** | 5.3 | 1.5 | 3.5 | 1.6 | 6E-08 | ** | 0.300 |  | 0.038 |  |
|  |  | ∠L_1_-P (°) | 9.3 | 2.3 | 7.4 | 4.3 | 0.018 |  | 9.8 | 3.5 | 6.2 | 5.6 | 9E-04 | ** | 0.430 |  | 0.081 |  |
|  |  | \|L_1_-L_2_\|/L_1_^L_2_ (%) | 97.4 | 1.1 | 98.6 | 0.8 | 1E-08 | ** | 97 | 1.2 | 98 | 1.3 | 2E-04 | ** | 0.005 | * | 8E-07 | ** |
|  | Ex-Ch//z | ∠L_1_-L_2_ (°) | 0.1 | 3.9 | 4.8 | 3.4 | 2E-09 | ** | -2.8 | 4.1 | -3.1 | 4.2 | 0.762 |  | 4E-05 | ** | 3E-42 | ** |
|  |  | \|L_1_-L_2_\| (%) | 83.5 | 5.4 | 79.6 | 4.3 | 8E-05 | ** | 79.6 | 5.9 | 70.8 | 4.8 | 1E-13 | ** | 2E-05 | ** | 4E-39 | ** |
|  |  | \|P\| (% to \|L_1_-L_2_\|) | 10.3 | 4.5 | 8.0 | 3.3 | 0.003 | * | 15.5 | 4.9 | 18.2 | 4.2 | 0.004 | * | 2E-06 | ** | 1E-43 | ** |
|  |  | \|L_1_-P\| (% to \|L_1_-L_2_\|) | 52.9 | 15.1 | 48.6 | 14.7 | 0.163 |  | 51.8 | 9.4 | 55.2 | 6.7 | 0.029 |  | 0.616 |  | 3E-05 | ** |
|  |  | **∫** (L_1_-L_2_) (% to \|L_1_-L_2_\|) | 6.8 | 1.9 | 5.3 | 1.9 | 5E-04 | ** | 9.4 | 2.4 | 10.7 | 2.5 | 0.011 |  | 3E-06 | ** | 3E-40 | ** |
|  |  | ∠L_1_-P (°) | 9.8 | 7.4 | 9.4 | 3.6 | 0.708 |  | 16.3 | 4.7 | 18.4 | 4.4 | 0.024 |  | 9E-06 | ** | 1E-34 | ** |
|  |  | \|L_1_-L_2_\|/L_1_^L_2_ (%) | 95.7 | 1.9 | 96.9 | 1.3 | 1E-04 | ** | 92.6 | 2.5 | 91.5 | 2.7 | 0.043 |  | 2E-06 | ** | 9E-37 | ** |
|  | Ac-Ch//z | ∠L_1_-L_2_ (°) | -12.9 | 5.6 | -6.6 | 5.6 | 4E-07 | ** | -19.8 | 6.5 | -23.0 | 6.3 | 0.017 |  | 7E-06 | ** | 1E-49 | ** |
|  |  | \|L_1_-L_2_\| (%) | 36.4 | 3.3 | 33.9 | 2.8 | 6E-05 | ** | 35.7 | 3.0 | 32.5 | 2.9 | 4E-07 | ** | 0.287 |  | 6E-06 | ** |
|  |  | \|P\| (% to \|L_1_-L_2_\|) | 16.8 | 4.3 | 12.6 | 5.2 | 7E-05 | ** | 16.0 | 4.7 | 11.3 | 4.6 | 3E-06 | ** | 0.493 |  | 0.048 |  |
|  |  | \|L_1_-P\| (% to \|L_1_-L_2_\|) | 63.5 | 12.3 | 61.7 | 20 | 0.63 |  | 62.7 | 12.7 | 63.9 | 10.6 | 0.606 |  | 0.801 |  | 0.273 |  |
|  |  | ∫ (L_1_-L_2_) (% to \|L_1_-L_2_\|) | 9.8 | 1.9 | 7.4 | 2.0 | 3E-08 | ** | 9.4 | 2.5 | 6.5 | 2.7 | 1E-06 | ** | 0.369 |  | 0.005 | * |
|  |  | ∠L_1_-P (°) | 13.4 | 9.2 | 9.7 | 7.8 | 0.03 |  | 13.4 | 6.3 | 9.8 | 5.3 | 0.002 | * | 0.986 |  | 0.932 |  |
|  |  | \|L_1_-L_2_\|/L_1_^L_2_ (%) | 91.2 | 1.7 | 93.8 | 2.0 | 2E-09 | ** | 91.6 | 2.5 | 95.6 | 2.7 | 4E-11 | ** | 0.399 |  | 4E-09 | ** |

* P < 0.01, ** P < 0.001. For definition of the variables, please see http://dx.doi.org/10.17632/wby6gkyfft.1

***S6 Table Contd.*** *Means and their standard deviations (S.D.) for the 28 variables for the contours Ex-Ac//z, En-Ac//z, Ex-Ch//z, and Ac-Ch//z for each side.*

| **Contour** | | **Variable** | **Smile - Rest** | | | | | |
| --- | --- | --- | --- | --- | --- | --- | --- | --- |
|  |  |  | **Older** | | **Younger** | | **P-value** | |
|  |  |  | **Mean** | **S.D.** | **Mean** | **S.D.** |  |  |
| Left | Ex-Ac//z | ∠L_1_-L_2_ (°) | 0.1 | 2.1 | -0.4 | 2.9 | 0.368 |  |
|  |  | \|L_1_-L_2_\| (%) | -1.7 | 2.6 | -2.4 | 2.6 | 0.169 |  |
|  |  | \|P\| (% to \|L_1_-L_2_\|) | 1.3 | 2.0 | 1.5 | 2.6 | 0.760 |  |
|  |  | \|L_1_-P\| (% to \|L_1_-L_2_\|) | -1.7 | 12.2 | 1.6 | 15.2 | 0.273 |  |
|  |  | ∫ (L_1_-L_2_) (% to \|L_1_-L_2_\|) | 1.2 | 1.7 | 1.5 | 1.8 | 0.343 |  |
|  |  | ∠L_1_-P (°) | 3.3 | 4.5 | 2.2 | 5.7 | 0.308 |  |
|  |  | \|L_1_-L_2_\|/L_1_^L_2_ (%) | -0.7 | 1.8 | -1.6 | 1.6 | 0.013 |  |
|  | En-Ac//z | ∠L_1_-L_2_ (°) | -0.3 | 1.7 | -1.3 | 2.3 | 0.017 |  |
|  |  | \|L_1_-L_2_\| (%) | -1.0 | 3.1 | -1.9 | 2.8 | 0.123 |  |
|  |  | \|P\| (% to \|L_1_-L_2_\|) | 0.1 | 4.2 | 0.6 | 4.1 | 0.519 |  |
|  |  | \|L_1_-P\| (% to \|L_1_-L_2_\|) | -1.8 | 19.0 | 0.1 | 35.1 | 0.779 |  |
|  |  | ∫ (L_1_-L_2_) (% to \|L_1_-L_2_\|) | 0.2 | 1.1 | 0.3 | 1.1 | 0.718 |  |
|  |  | ∠L_1_-P (°) | 0.5 | 3.1 | -0.4 | 6.8 | 0.469 |  |
|  |  | \|L_1_-L_2_\|/L_1_^L_2_ (%) | -0.5 | 1.1 | -0.4 | 1.1 | 0.901 |  |
|  | Ex-Ch//z | ∠L_1_-L_2_ (°) | -3.0 | 2.9 | -7.7 | 3.8 | 3E-09 | ** |
|  |  | \|L_1_-L_2_\| (%) | -3.8 | 4.2 | -8.5 | 4.0 | 1E-07 | ** |
|  |  | \|P\| (% to \|L_1_-L_2_\|) | 4.9 | 4.4 | 10.8 | 4.3 | 1E-09 | ** |
|  |  | \|L_1_-P\| (% to \|L_1_-L_2_\|) | 1.2 | 21.4 | 11.3 | 17.8 | 0.01 | * |
|  |  | ∫ (L_1_-L_2_) (% to \|L_1_-L_2_\|) | 2.5 | 1.9 | 5.7 | 2.5 | 4E-09 | ** |
|  |  | ∠L_1_-P (°) | 5.5 | 5.7 | 8.3 | 7.2 | 0.052 |  |
|  |  | \|L_1_-L_2_\|/L_1_^L_2_ (%) | -3.1 | 1.9 | -5.1 | 2.7 | 4E-04 | ** |
|  | Ac-Ch//z | ∠L_1_-L_2_ (°) | -6.8 | 5.3 | -17.5 | 6.1 | 2E-14 | ** |
|  |  | \|L_1_-L_2_\| (%) | -0.8 | 3.4 | -1.5 | 3.3 | 0.325 |  |
|  |  | \|P\| (% to \|L_1_-L_2_\|) | 0.5 | 6.7 | 0.7 | 7.7 | 0.913 |  |
|  |  | \|L_1_-P\| (% to \|L_1_-L_2_\|) | 2.0 | 19.9 | 10.8 | 29.6 | 0.127 |  |
|  |  | ∫ (L_1_-L_2_) (% to \|L_1_-L_2_\|) | -0.1 | 2.2 | -0.8 | 2.8 | 0.214 |  |
|  |  | ∠L_1_-P (°) | 1.2 | 5.3 | 0.0 | 6.1 | 0.353 |  |
|  |  | \|L_1_-L_2_\|/L_1_^L_2_ (%) | 0.0 | 2.8 | 1.6 | 3.3 | 0.013 |  |
| Right | Ex-Ac//z | ∠L_1_-L_2_ (°) | 0.3 | 2.2 | -1.0 | 2.7 | 0.02 |  |
|  |  | \|L_1_-L_2_\| (%) | -1.7 | 2.7 | -3.3 | 2.4 | 0.002 | * |
|  |  | \|P\| (% to \|L_1_-L_2_\|) | 1.3 | 2.2 | 1.6 | 2.8 | 0.543 |  |
|  |  | \|L_1_-P\| (% to \|L_1_-L_2_\|) | -1.2 | 3.8 | 4.7 | 15.2 | 0.037 |  |
|  |  | ∫ (L_1_-L_2_) (% to \|L_1_-L_2_\|) | 1.0 | 1.4 | 1.4 | 1.7 | 0.241 |  |
|  |  | ∠L_1_-P (°) | 2.5 | 3.9 | 1.9 | 5.2 | 0.563 |  |
|  |  | \|L_1_-L_2_\|/L_1_^L_2_ (%) | -0.6 | 1.5 | -1.5 | 1.4 | 0.003 | * |
|  | En-Ac//z | ∠L_1_-L_2_ (°) | -0.2 | 1.6 | -1.3 | 2.3 | 0.012 |  |
|  |  | \|L_1_-L_2_\| (%) | -1.1 | 3.2 | -1.9 | 2.8 | 0.178 |  |
|  |  | \|P\| (% to \|L_1_-L_2_\|) | 0.2 | 3.6 | -0.2 | 3.3 | 0.650 |  |
|  |  | \|L_1_-P\| (% to \|L_1_-L_2_\|) | -0.5 | 19.0 | -1.8 | 27.9 | 0.810 |  |
|  |  | ∫ (L_1_-L_2_) (% to \|L_1_-L_2_\|) | 0.2 | 1.0 | 0.3 | 1.2 | 0.805 |  |
|  |  | ∠L_1_-P (°) | 0.5 | 3.5 | -1.2 | 6.6 | 0.187 |  |
|  |  | \|L_1_-L_2_\|/L_1_^L_2_ (%) | -0.5 | 0.9 | -0.6 | 1.1 | 0.681 |  |
|  | Ex-Ch//z | ∠L_1_-L_2_ (°) | -2.9 | 3.3 | -7.9 | 3.3 | 5E-11 | ** |
|  |  | \|L_1_-L_2_\| (%) | -3.9 | 4.2 | -8.8 | 4.1 | 7E-08 | ** |
|  |  | \|P\| (% to \|L_1_-L_2_\|) | 5.2 | 4.8 | 10.2 | 4.2 | 2E-07 | ** |
|  |  | \|L_1_-P\| (% to \|L_1_-L_2_\|) | -1.1 | 11.6 | 6.7 | 15.3 | 0.012 |  |
|  |  | **∫** (L_1_-L_2_) (% to \|L_1_-L_2_\|) | 2.6 | 2.4 | 5.4 | 2.4 | 2E-07 | ** |
|  |  | ∠L_1_-P (°) | 6.5 | 6.6 | 9.0 | 4.8 | 0.024 |  |
|  |  | \|L_1_-L_2_\|/L_1_^L_2_ (%) | -3.0 | 2.8 | -5.4 | 2.7 | 5E-05 | ** |
|  | Ac-Ch//z | ∠L_1_-L_2_ (°) | -7.0 | 7.0 | -16.4 | 5.7 | 1E-11 | ** |
|  |  | \|L_1_-L_2_\| (%) | -0.7 | 3.6 | -1.5 | 3.0 | 0.260 |  |
|  |  | \|P\| (% to \|L_1_-L_2_\|) | -0.8 | 6.5 | -1.2 | 6.1 | 0.763 |  |
|  |  | \|L_1_-P\| (% to \|L_1_-L_2_\|) | -0.8 | 17.7 | 2.2 | 20.2 | 0.457 |  |
|  |  | ∫ (L_1_-L_2_) (% to \|L_1_-L_2_\|) | -0.4 | 2.5 | -0.8 | 2.9 | 0.487 |  |
|  |  | ∠L_1_-P (°) | 0.0 | 11.5 | 0.1 | 8.5 | 0.985 |  |
|  |  | \|L_1_-L_2_\|/L_1_^L_2_ (%) | 0.4 | 2.6 | 1.8 | 2.8 | 0.017 |  |

* P < 0.01; ** P < 0.001. For definition of the variables, please see S2 Fig.
