# Supplementary material for: Functional decline in facial expression generation in older women: A cross-sectional study using three-dimensional morphometry
Source: PLoS One. 2019 Jul 10;14(7):e0219451. doi: 10.1371/journal.pone.0219451 (PMC6636602; doi:10.1371/journal.pone.0219451)
Supplement: S7 Table — (DOCX) [file pone.0219451.s009.docx]

***S7 Table.*** *Means and their standard deviations (S.D.) for the 21 variables for the contours N//sagittal and Prn//sagittal.*

| **Contour** | **Variable** | **Rest** | | | | | | **Smile** | | | | | | **P-value (Rest vs. Smile)** | | | |
| --- | --- | --- | --- | --- | --- | --- | --- | --- | --- | --- | --- | --- | --- | --- | --- | --- | --- |
|  |  | **Older** | | **Younger** | | **P-value** |  | **Older** | | **Younger** | | **P-value** |  | **Older** | | **Younger** | |
|  |  | **Mean** | **S.D.** | **Mean** | **S.D.** |  |  | **Mean** | **S.D.** | **Mean** | **S.D.** |  |  |  |  |  |  |
| N//sagittal | v1 (% to \|N-Sn\|) | 0.6 | 3.3 | 0.0 | 0.0 | 0.068 |  | 0.0 | 0.0 | 0.0 | 0.2 | 0.586 |  | 0.325 |  | 0.320 |  |
|  | v2 (% to \|N-Sn\|) | 7.2 | 4.7 | 7.7 | 3.3 | 0.495 |  | 7.2 | 3.6 | 7.7 | 3.4 | 0.554 |  | 0.927 |  | 0.881 |  |
|  | v3 (% to \|N-Sn\|) | 29.7 | 6.6 | 32.3 | 7.2 | 0.08 |  | 28.8 | 5.5 | 31.3 | 6.9 | 0.070 |  | 0.044 |  | 0.008 | * |
|  | v4 (% to \|N-Sn\|) | 33.6 | 7.5 | 30.5 | 7.8 | 0.057 |  | 34.2 | 7.4 | 30.9 | 7.3 | 0.032 |  | 0.656 |  | 0.364 |  |
|  | v5 (% to \|N-Sn\|) | 11.5 | 1.4 | 13 | 1.8 | 1E-05 | ** | 11.9 | 1.7 | 13.4 | 2.3 | 9E-04 | ** | 0.182 |  | 0.121 |  |
|  | v6 (% to \|N-Sn\|) | 3.9 | 2.3 | 2.7 | 2.0 | 0.006 | * | 4.1 | 1.9 | 3.0 | 2.5 | 0.034 |  | 0.283 |  | 0.003 | * |
|  | v7 (°) | 27.7 | 3.3 | 28.9 | 3.5 | 0.097 |  | 28.1 | 3.7 | 28 | 3.6 | 0.936 |  | 0.042 |  | 7E-07 | ** |
|  | v8 (°) | 54.9 | 5.0 | 52.1 | 5.3 | 0.012 |  | 55.3 | 4.8 | 54.4 | 7.5 | 0.528 |  | 0.501 |  | 5E-04 | ** |
| Prn/sagittal | v1 (°) | 80.4 | 4.8 | 79.8 | 5.1 | 0.55 |  | 80.4 | 4.5 | 80.3 | 4.9 | 0.918 |  | 0.955 |  | 0.017 |  |
|  | v2 (°) | 116.3 | 9.5 | 118.8 | 9.3 | 0.207 |  | 116.1 | 9.9 | 118.9 | 8.8 | 0.139 |  | 0.790 |  | 0.869 |  |
|  | v3 (°) | 134.9 | 13.6 | 126.9 | 14.3 | 0.008 | * | 139.5 | 11.8 | 148.6 | 14.3 | 0.002 | * | 0.003 | * | 8E-26 | ** |
|  | v4 (°) | 82.8 | 6.2 | 81.1 | 6.6 | 0.21 |  | 83.5 | 4.3 | 80.0 | 8.3 | 0.027 |  | 0.275 |  | 0.155 |  |
|  | v5 (%) | 19.6 | 2.9 | 15.4 | 3.0 | 3E-10 | ** | 16.5 | 3.8 | 10.6 | 3.3 | 2E-13 | ** | 1E-05 | ** | 2E-27 | ** |
|  | v6 (% to \|Prn-Pog\|) | -2.2 | 3.6 | -3.8 | 3.5 | 0.034 |  | -5.1 | 4.4 | -9.3 | 4.1 | 4E-06 | ** | 2E-06 | ** | 2E-37 | ** |
|  | v7 (% to \|Prn-Pog\|) | -0.7 | 4.0 | 0.4 | 4.2 | 0.24 |  | -2.5 | 3.7 | -6.5 | 4.4 | 2E-05 | ** | 8E-05 | ** | 3E-39 | ** |
|  | v8 (% to \|Prn-Pog\|) | -2.9 | 7.2 | -3.4 | 7.3 | 0.72 |  | -7.6 | 7.4 | -15.7 | 7.7 | 1E-06 | ** | 2E-06 | ** | 4E-47 | ** |
|  | v9 (% to \|Prn-Pog\|) | 1.6 | 2.5 | 4.2 | 2.6 | 4E-06 | ** | 2.6 | 3.5 | 2.8 | 3.6 | 0.757 |  | 0.025 |  | 3E-04 | ** |
|  | v10 (% to \|Prn-Pog\|) | 9.2 | 2.9 | 8.6 | 2.8 | 0.337 |  | 9.3 | 3.0 | 9.5 | 3.3 | 0.794 |  | 0.712 |  | 0.006 | * |
|  | v11 (% to \|Prn-Pog\|) | 4.7 | 1.9 | 5.7 | 2.3 | 0.033 |  | 3.4 | 1.9 | -0.4 | 3.7 | 3E-07 | ** | 7E-04 | ** | 1E-25 | ** |
|  | v12 (°) | 177.9 | 7.2 | 163.2 | 7.0 | 6E-18 | ** | 174.9 | 9.8 | 162.4 | 8.0 | 6E-11 | ** | 0.006 | * | 0.34 |  |
|  | v13 (% to \|Prn-Pog\|) | 26.8 | 4.5 | 31.2 | 3.9 | 7E-07 | ** | 30.8 | 5.4 | 33.1 | 7.3 | 0.113 |  | 3E-05 | ** | 0.012 |  |

* P < 0.01; ** P < 0.001. For definition of the variables, please see S3 Fig and S4 Fig.

***S7 Table Contd.*** *Means and their standard deviations (S.D.) for the 21 variables for the contours N//sagittal and Prn//sagittal.*

| **Contour** | **Variable** | **Smile - Rest** | | | | | |
| --- | --- | --- | --- | --- | --- | --- | --- |
|  |  | **Older** | | **Younger** | | **P-value** |  |
|  |  | **Mean** | **S.D.** | **Mean** | **S.D.** |  |  |
| N//sagittal | v1 (% to \|N-Sn\|) | -0.6 | 3.3 | 0.0 | 0.2 | 0.061 |  |
|  | v2 (% to \|N-Sn\|) | 0.1 | 4.0 | 0.0 | 2.0 | 0.858 |  |
|  | v3 (% to \|N-Sn\|) | -0.9 | 2.4 | -1.0 | 3.7 | 0.912 |  |
|  | v4 (% to \|N-Sn\|) | 0.6 | 7.2 | 0.4 | 4.3 | 0.854 |  |
|  | v5 (% to \|N-Sn\|) | 0.4 | 1.6 | 0.3 | 2.2 | 0.881 |  |
|  | v6 (% to \|N-Sn\|) | 0.2 | 1.2 | 0.4 | 1.3 | 0.561 |  |
|  | v7 (°) | 0.4 | 1.1 | -0.8 | 1.6 | 9E-05 | ** |
|  | v8 (°) | 0.5 | 3.7 | 2.3 | 6.5 | 0.134 |  |
| Prn/sagittal | v1 (°) | 0.0 | 1.8 | 0.5 | 2.2 | 0.244 |  |
|  | v2 (°) | -0.2 | 4.0 | 0.1 | 8.1 | 0.830 |  |
|  | v3 (°) | 4.6 | 7.9 | 21.6 | 15.1 | 3E-08 | ** |
|  | v4 (°) | 0.7 | 3.5 | -1.1 | 7.9 | 0.218 |  |
|  | v5 (%) | -3.1 | 3.2 | -4.7 | 3.1 | 0.014 |  |
|  | v6 (% to \|Prn-Pog\|) | -2.9 | 2.7 | -5.5 | 2.7 | 7E-06 | ** |
|  | v7 (% to \|Prn-Pog\|) | -1.9 | 2.2 | -6.8 | 3.2 | 6E-13 | ** |
|  | v8 (% to \|Prn-Pog\|) | -4.7 | 4.4 | -12.3 | 4.6 | 6E-13 | ** |
|  | v9 (% to \|Prn-Pog\|) | 1.0 | 2.3 | -1.4 | 3.6 | 1E-03 | ** |
|  | v10 (% to \|Prn-Pog\|) | 0.1 | 1.5 | 0.8 | 3.0 | 0.194 |  |
|  | v11 (% to \|Prn-Pog\|) | -1.3 | 1.8 | -6.1 | 4.3 | 2E-08 | ** |
|  | v12 (°) | -3.0 | 5.6 | -0.8 | 8.7 | 0.200 |  |
|  | v13 (% to \|Prn-Pog\|) | 4.1 | 4.5 | 1.9 | 7.6 | 0.148 |  |

* P < 0.01; ** P < 0.001. For definition of the variables, please see S3 Fig and S4 Fig.
