# Supplementary material for: Functional decline in facial expression generation in older women: A cross-sectional study using three-dimensional morphometry
Source: PLoS One. 2019 Jul 10;14(7):e0219451. doi: 10.1371/journal.pone.0219451 (PMC6636602; doi:10.1371/journal.pone.0219451)
Supplement: S8 Table — (DOCX) [file pone.0219451.s010.docx]

***S8 Table.*** *Means and their standard deviations (S.D.) for the 58 variables for the contours Gla//axial, N//axial, Or//axial, Prn//axial, Sn//axial, Ls//axial, Li//axial, and Sm//axial.*

|  |  | **Rest** | | | | | | | **Smile** | | | | | | | | **P-value (Rest vs. Smile)** | | | |
| --- | --- | --- | --- | --- | --- | --- | --- | --- | --- | --- | --- | --- | --- | --- | --- | --- | --- | --- | --- | --- |
| **Contour** | **Variable** | **Older** | | **Younger** | | |  | | **Older** | | | **Younger** | | | **P-value** | | **Older** | | **Younger** | |
|  |  | **Mean** | **S.D.** | **Mean** | **S.D.** | |  |  | **Mean** | **S.D.** | | **Mean** | **S.D.** | |  |  |  |  |  |  |
| Gla//axial | \|E-E\| (% to \|Ex-Ex\|) | 144.4 | 4.5 | 139.6 | | 6.7 | 3E-04 | ** | 145 | 6.1 | 137.6 | | 7.1 | 1E-06 | | ** | 0.448 |  | 6E-05 | ** |
|  | \|M\| (% to \|E-E\|) | 15.7 | 1.8 | 16.0 | | 2.1 | 0.374 |  | 16.1 | 1.7 | 15.9 | | 1.9 | 0.491 | |  | 6E-04 | ** | 0.258 |  |
|  | ∠E-P-M (°, left) | 137.5 | 4.7 | 139.9 | | 5.4 | 0.032 |  | 137.2 | 5.4 | 139.7 | | 5.3 | 0.024 | |  | 0.437 |  | 0.615 |  |
|  | \|P\| (% to \|E-E\|, left) | 11.6 | 2.8 | 11.3 | | 4.1 | 0.742 |  | 12.3 | 3.1 | 11.1 | | 3.4 | 0.083 | |  | 0.004 | * | 0.417 |  |
|  | ∠E-P-M (°, right) | 137.1 | 6.1 | 140.1 | | 4.8 | 0.006 | * | 136.9 | 5.1 | 139.6 | | 5.0 | 0.011 | |  | 0.693 |  | 0.091 |  |
|  | \|P\| (% to \|E-E\|, right) | 12.4 | 3.3 | 11.0 | | 2.9 | 0.022 |  | 12.2 | 3.2 | 11.2 | | 3.5 | 0.1415 | |  | 0.341 |  | 0.429 |  |
| N//axial | \|E-E\| (% to \|Ex-Ex\|) | 141.5 | 6.4 | 141.5 | | 7.6 | 0.996 |  | 142.4 | 7.5 | 140.1 | | 7.9 | 0.16 | |  | 0.338 |  | 0.013 |  |
|  | \|M\| (% to \|E-E\|) | 11.9 | 1.2 | 13.3 | | 1 | 5E-10 | ** | 11.8 | 1.3 | 13 | | 1.1 | 2E-06 | | ** | 0.278 |  | 1E-05 | ** |
|  | ∠E-P-M (°, left) | 144.7 | 17.9 | 141.3 | | 6.4 | 0.120 |  | 144.4 | 14.6 | 142.6 | | 6.4 | 0.344 | |  | 0.885 |  | 0.046 |  |
|  | \|P\| (% to \|E-E\|, left) | 6.9 | 2.0 | 8.3 | | 1.8 | 6E-04 | ** | 6.7 | 2.1 | 7.7 | | 2.0 | 0.025 | |  | 0.276 |  | 0.001 | * |
|  | ∠E-P-M (°, right) | 140.4 | 8.8 | 142.9 | | 5.4 | 0.066 |  | 141.8 | 10.6 | 144.1 | | 6.7 | 0.165 | |  | 0.136 |  | 0.066 |  |
|  | \|P\| (% to \|E-E\|, right) | 7.1 | 2.4 | 7.8 | | 1.7 | 0.083 |  | 7.2 | 2.0 | 7.2 | | 1.9 | 0.916 | |  | 0.734 |  | 4E-05 | ** |
| Or//axial | \|E-E\| (% to \|Ex-Ex\|) | 146.7 | 5.3 | 142.2 | | 6.7 | 0.001 | * | 146.5 | 6.3 | 141 | | 6.9 | 2E-04 | | ** | 0.762 |  | 0.016 |  |
|  | \|M\| (% to \|E-E\|) | 28.0 | 2.5 | 28.8 | | 3.1 | 0.233 |  | 26.7 | 3.0 | 26.5 | | 3.1 | 0.736 | |  | 3E-04 | ** | 3E-22 | ** |
|  | \|N-N\| (% to \|E-E\|) | 23.8 | 7.4 | 21.7 | | 3.3 | 0.027 |  | 24.8 | 7.4 | 21.7 | | 3.0 | 9E-04 | | ** | 0.555 |  | 0.97 |  |
|  | ∠E-P-N (°, left) | 105.4 | 3.4 | 106.4 | | 2.8 | 0.088 |  | 103.5 | 4.5 | 103.5 | | 3.4 | 0.991 | |  | 0.041 |  | 2E-18 | ** |
|  | ∠N-M (°, left) | 55.8 | 10.0 | 53.7 | | 6.6 | 0.193 |  | 55.2 | 5.8 | 54.5 | | 5.3 | 0.529 | |  | 0.683 |  | 0.029 |  |
|  | \|P\| (% to \|E-E\|, left) | 11.2 | 2.3 | 11.4 | | 1.3 | 0.501 |  | 11.7 | 2.5 | 12.3 | | 1.8 | 0.143 | |  | 0.341 |  | 3E-09 | ** |
|  | \|NP\| (% to \|E-E\|, left) | 1.8 | 0.8 | 2.3 | | 1.1 | 0.025 |  | 2.0 | 0.8 | 2.3 | | 1.0 | 0.187 | |  | 0.190 |  | 0.45 |  |
|  | ∠E-P-N (°, right) | 105.8 | 2.9 | 106.7 | | 2.4 | 0.095 |  | 104.2 | 3.5 | 103.9 | | 3.4 | 0.684 | |  | 0.008 | * | 1E-15 | ** |
|  | ∠N-M (°, right) | 56 | 5.6 | 54.1 | | 6.9 | 0.192 |  | 55.9 | 4.2 | 55.1 | | 7.2 | 0.592 | |  | 0.865 |  | 0.014 |  |
|  | \|P\| (% to \|E-E\|, right) | 10.9 | 1.5 | 11.5 | | 1.5 | 0.054 |  | 11.5 | 1.8 | 12.4 | | 1.8 | 0.024 | |  | 0.007 | * | 9E-11 | ** |
|  | \|NP\| (% to \|E-E\|, right) | 2.4 | 1.2 | 1.8 | | 1.0 | 0.005 | * | 2.5 | 1.1 | 1.8 | | 1.0 | 7E-04 | | ** | 0.348 |  | 0.732 |  |
| Prn//axial | \|E-E\| (% to \|Ex-Ex\|) | 141.6 | 5.5 | 135 | | 7.0 | 6E-06 | ** | 142.1 | 7.1 | 135.6 | | 7.3 | 3E-05 | | ** | 0.512 |  | 0.229 |  |
|  | \|M\| (% to \|E-E\|) | 34.4 | 3.3 | 35.4 | | 3.0 | 0.118 |  | 32.5 | 3.8 | 31.7 | | 3.1 | 0.283 | |  | 8E-07 | ** | 1E-34 | ** |
|  | \|N-N\| (% to \|E-E\|) | 29 | 7.7 | 29.1 | | 2.6 | 0.949 |  | 29.0 | 4.0 | 31.6 | | 3.0 | 2E-04 | | ** | 0.967 |  | 8E-14 | ** |
|  | ∠E-P-N (°, left) | 109.6 | 8.0 | 104.6 | | 4.4 | 2E-05 | ** | 105.5 | 9.6 | 100.1 | | 5.8 | 2E-04 | | ** | 0.028 |  | 4E-12 | ** |
|  | ∠N-M (°, left) | 48.2 | 8.0 | 41.4 | | 4.7 | 7E-08 | ** | 48.3 | 7.2 | 45.8 | | 5.7 | 0.046 | |  | 0.917 |  | 1E-15 | ** |
|  | \|P\| (% to \|E-E\|, left) | 9.1 | 2.9 | 10.9 | | 1.6 | 3E-05 | ** | 10.8 | 2.6 | 12.3 | | 1.9 | 4E-04 | | ** | 0.004 | * | 1E-12 | ** |
|  | \|NP\| (% to \|E-E\|, left) | 3.9 | 1.8 | 5.8 | | 1.4 | 2E-08 | ** | 3.9 | 1.9 | 5.1 | | 1.7 | 0.001 | | * | 0.934 |  | 8E-08 | ** |
|  | ∠E-P-N (°, right) | 106.3 | 8.6 | 104 | | 3.8 | 0.037 |  | 104.2 | 8.8 | 98.2 | | 4.3 | 1E-06 | | ** | 0.301 |  | 2E-22 | ** |
|  | ∠N-M (°, right) | 46.6 | 7.7 | 41.6 | | 4.8 | 3E-05 | ** | 48.9 | 7.2 | 44.7 | | 4.1 | 9E-05 | | ** | 0.104 |  | 7E-12 | ** |
|  | \|P\| (% to \|E-E\|, right) | 10.4 | 2.7 | 11.3 | | 1.5 | 0.028 |  | 11.0 | 2.5 | 13.1 | | 1.8 | 1E-06 | | ** | 0.259 |  | 4E-22 | ** |
|  | \|NP\| (% to \|E-E\|, right) | 5.0 | 1.4 | 5.4 | | 1.5 | 0.175 |  | 4.8 | 1.7 | 5.1 | | 1.4 | 0.276 | |  | 0.513 |  | 0.033 |  |
| Sn//axial | \|E-E\| (% to \|Ex-Ex\|) | 137.7 | 4.8 | 129.9 | | 7.2 | 1E-07 | ** | 139.4 | 6.5 | 133.4 | | 7.5 | 1E-04 | | ** | 0.040 |  | 3E-10 | ** |
|  | \|M\| (% to \|E-E\|) | 13.1 | 1.8 | 13.9 | | 1.6 | 0.033 |  | 11.7 | 2.0 | 12.0 | | 1.4 | 0.365 | |  | 9E-07 | ** | 8E-32 | ** |
|  | ∠E-P-M (°, left) | 145.3 | 6.8 | 147 | | 7.9 | 0.275 |  | 145.1 | 9.7 | 144.3 | | 9.6 | 0.688 | |  | 0.913 |  | 0.007 | * |
|  | \|P\| (% to \|E-E\|, left) | 7.0 | 1.4 | 7.2 | | 1.2 | 0.482 |  | 6.7 | 1.2 | 6.9 | | 1.2 | 0.487 | |  | 0.115 |  | 0.022 |  |
|  | ∠E-P-M (°, right) | 144.4 | 7.0 | 146.4 | | 7.6 | 0.202 |  | 145 | 11 | 143.1 | | 8.4 | 0.313 | |  | 0.758 |  | 7E-04 | ** |
|  | \|P\| (% to \|E-E\|, right) | 7.0 | 1.1 | 7.3 | | 1.1 | 0.137 |  | 7.0 | 1.2 | 6.9 | | 1.2 | 0.880 | |  | 0.859 |  | 5E-04 | ** |
| Ls//axial | \|E-E\| (% to \|Ex-Ex\|) | 132.2 | 6.3 | 125.2 | | 7.5 | 1E-05 | ** | 138.1 | 6.9 | 133.3 | | 7.5 | 0.002 | | * | 8E-06 | ** | 4E-27 | ** |
|  | \|M\| (% to \|E-E\|) | 16.0 | 1.6 | 16.7 | | 1.6 | 0.033 |  | 15.4 | 1.8 | 14.6 | | 1.5 | 0.017 | |  | 0.011 |  | 6E-29 | ** |
|  | ∠E-P-M (°, left) | 158.5 | 7.3 | 153.3 | | 4.6 | 6E-06 | ** | 161.3 | 9.6 | 158.3 | | 10.4 | 0.163 | |  | 0.178 |  | 1E-05 | ** |
|  | \|P\| (% to \|E-E\|, left) | 6.2 | 1.4 | 7.6 | | 1.5 | 2E-05 | ** | 5.8 | 1.1 | 5.9 | | 1.1 | 0.711 | |  | 0.064 |  | 2E-16 | ** |
|  | ∠E-P-M (°, right) | 159.7 | 7.1 | 152.4 | | 4.4 | 3E-10 | ** | 159.6 | 9.6 | 158.4 | | 10.7 | 0.557 | |  | 0.964 |  | 1E-07 | ** |
|  | \|P\| (% to \|E-E\|, right) | 6.0 | 1.6 | 7.5 | | 1.4 | 7E-07 | ** | 6.0 | 1.3 | 5.9 | | 1.3 | 0.617 | |  | 0.922 |  | 2E-16 | ** |
| Li//axial | \|E-E\| (% to \|Ex-Ex\|) | 124.1 | 7.4 | 112.7 | | 8.4 | 6E-10 | ** | 127.1 | 8.2 | 118.6 | | 8.4 | 3E-06 | | ** | 0.003 | * | 1E-15 | ** |
|  | \|M\| (% to \|E-E\|) | 17.2 | 1.8 | 18.7 | | 1.6 | 4E-05 | ** | 16.9 | 1.5 | 16.5 | | 1.8 | 0.352 | |  | 0.063 |  | 3E-26 | ** |
|  | ∠E-P-M (°, left) | 165.3 | 9.2 | 159 | | 6.4 | 5E-05 | ** | 162.7 | 8.3 | 160.6 | | 6.6 | 0.155 | |  | 0.277 |  | 0.085 |  |
|  | \|P\| (% to \|E-E\|, left) | 6.4 | 2.0 | 8.4 | | 1.7 | 2E-07 | ** | 5.5 | 1.7 | 5.8 | | 1.5 | 0.454 | |  | 0.005 | * | 2E-25 | ** |
|  | ∠E-P-M (°, right) | 164.9 | 9.0 | 158.9 | | 6.3 | 7E-05 | ** | 165.4 | 7.0 | 161.9 | | 7.2 | 0.018 | |  | 0.818 |  | 0.002 | * |
|  | \|P\| (% to \|E-E\|, right) | 5.7 | 1.3 | 7.8 | | 1.7 | 4E-09 | ** | 5.0 | 1.3 | 5.1 | | 1.2 | 0.681 | |  | 0.039 |  | 3E-30 | ** |
| Sm//axial | \|E-E\| (% to \|Ex-Ex\|) | 119.8 | 8.0 | 109.5 | | 9.4 | 3E-07 | ** | 120.5 | 9.7 | 110.9 | | 9.9 | 7E-06 | | ** | 0.437 |  | 0.042 |  |
|  | \|M\| (% to \|E-E\|) | 19.0 | 3.1 | 19.2 | | 1.7 | 0.574 |  | 19.1 | 5.4 | 18.6 | | 1.9 | 0.417 | |  | 0.910 |  | 4E-04 | ** |
|  | ∠E-P-M (°, left) | 152.5 | 8.9 | 149.2 | | 4.3 | 0.005 | * | 153.2 | 8.5 | 154.1 | | 6.4 | 0.521 | |  | 0.746 |  | 3E-10 | ** |
|  | \|P\| (% to \|E-E\|, left) | 12.0 | 6.1 | 12.4 | | 2.5 | 0.609 |  | 11.2 | 8.1 | 10.0 | | 2.9 | 0.201 | |  | 0.660 |  | 8E-12 | ** |
|  | ∠E-P-M (°, right) | 155.6 | 5.3 | 149.7 | | 3.9 | 6E-10 | ** | 155.4 | 5.8 | 154.3 | | 6.5 | 0.429 | |  | 0.777 |  | 2E-11 | ** |
|  | \|P\| (% to \|E-E\|, right) | 8.6 | 1.6 | 10.6 | | 2.1 | 6E-06 | ** | 8.1 | 2.1 | 9.0 | | 2.2 | 0.059 | |  | 0.261 |  | 1E-09 | ** |

* P < 0.01; ** P < 0.001. For definition of the variables, please see S5 Fig.

***S8 Table Contd.*** *Means and their standard deviations (S.D.) for the 58 variables for the contours Gla//axial, N//axial, Or//axial, Prn//axial, Sn//axial, Ls//axial, Li//axial, and Sm//axial.*

|  |  | **Smile - Rest** | | | | | |
| --- | --- | --- | --- | --- | --- | --- | --- |
| **Contour** | **Variable** | **Older** | | **Younger** | |  | |
|  |  | **Mean** | **S.D.** | **Mean** | **S.D.** |  |  |
| Gla//axial | \|E-E\| (%) | 0.6 | 4.0 | -2.0 | 4.8 | 0.008 | * |
|  | \|M\| (% to \|E-E\|) | 0.5 | 0.7 | -0.2 | 1.5 | 0.026 |  |
|  | ∠E-P-M (°, left) | -0.3 | 2.1 | -0.2 | 3.6 | 0.869 |  |
|  | \|P\| (% to \|E-E\|, left) | 0.7 | 1.3 | -0.3 | 3.2 | 0.114 |  |
|  | ∠E-P-M (°, right) | -0.2 | 2.5 | -0.5 | 2.8 | 0.600 |  |
|  | \|P\| (% to \|E-E\|, right) | -0.2 | 1.2 | 0.2 | 2.5 | 0.386 |  |
| N//axial | \|E-E\| (%) | 0.8 | 4.8 | -1.4 | 5.5 | 0.046 |  |
|  | \|M\| (% to \|E-E\|) | -0.1 | 0.5 | -0.4 | 0.8 | 0.073 |  |
|  | ∠E-P-M (°, left) | -0.3 | 10.8 | 1.3 | 6.3 | 0.324 |  |
|  | \|P\| (% to \|E-E\|, left) | -0.2 | 1.1 | -0.6 | 1.8 | 0.267 |  |
|  | ∠E-P-M (°, right) | 1.4 | 4.9 | 1.2 | 6.4 | 0.882 |  |
|  | \|P\| (% to \|E-E\|, right) | 0.1 | 1.5 | -0.6 | 1.5 | 0.021 |  |
| Or//axial | \|E-E\| (%) | -0.2 | 4.2 | -1.2 | 5.0 | 0.328 |  |
|  | \|M\| (% to \|E-E\|) | -1.3 | 1.7 | -2.2 | 1.8 | 0.010 | * |
|  | \|N-N\| (% to \|E-E\|) | 1.0 | 9.2 | 0.0 | 2.5 | 0.333 |  |
|  | ∠E-P-N (°, left) | -1.9 | 4.8 | -2.9 | 2.7 | 0.129 |  |
|  | ∠N-M (°, left) | -0.6 | 7.9 | 0.7 | 3.3 | 0.181 |  |
|  | \|P\| (% to \|E-E\|, left) | 0.5 | 2.7 | 0.9 | 1.3 | 0.296 |  |
|  | \|NP\| (% to \|E-E\|, left) | 0.2 | 0.8 | 0.0 | 0.6 | 0.080 |  |
|  | ∠E-P-N (°, right) | -1.6 | 3.1 | -2.8 | 2.9 | 0.058 |  |
|  | ∠N-M (°, right) | -0.1 | 3.0 | 1.0 | 3.9 | 0.168 |  |
|  | \|P\| (% to \|E-E\|, right) | 0.6 | 1.2 | 0.9 | 1.2 | 0.281 |  |
|  | \|NP\| (% to \|E-E\|, right) | 0.1 | 0.6 | 0.0 | 0.6 | 0.352 |  |
| Prn//axial | \|E-E\| (%) | 0.5 | 4.3 | 0.6 | 4.6 | 0.971 |  |
|  | \|M\| (% to \|E-E\|) | -1.9 | 1.7 | -3.7 | 1.9 | 2E-05 | ** |
|  | \|N-N\| (% to \|E-E\|) | -0.1 | 7.2 | 2.5 | 2.8 | 0.005 | * |
|  | ∠E-P-N (°, left) | -4.2 | 9.9 | -4.5 | 5.7 | 0.808 |  |
|  | ∠N-M (°, left) | 0.1 | 7.2 | 4.3 | 4.5 | 2E-04 | ** |
|  | \|P\| (% to \|E-E\|, left) | 1.6 | 2.9 | 1.4 | 1.7 | 0.640 |  |
|  | \|NP\| (% to \|E-E\|, left) | 0.0 | 1.7 | -0.7 | 1.3 | 0.013 |  |
|  | ∠E-P-N (°, right) | -2.1 | 10.9 | -5.8 | 4.6 | 0.008 | * |
|  | ∠N-M (°, right) | 2.3 | 7.5 | 3.1 | 4.0 | 0.432 |  |
|  | \|P\| (% to \|E-E\|, right) | 0.6 | 2.6 | 1.8 | 1.4 | 1E-03 | ** |
|  | \|NP\| (% to \|E-E\|, right) | -0.2 | 1.6 | -0.3 | 1.2 | 0.781 |  |
| Sn//axial | \|E-E\| (%) | 1.7 | 4.2 | 3.6 | 5.1 | 0.064 |  |
|  | \|M\| (% to \|E-E\|) | -1.4 | 1.3 | -1.9 | 1.1 | 0.062 |  |
|  | ∠E-P-M (°, left) | -0.2 | 8.0 | -2.7 | 9.7 | 0.194 |  |
|  | \|P\| (% to \|E-E\|, left) | -0.3 | 1.0 | -0.3 | 1.3 | 0.973 |  |
|  | ∠E-P-M (°, right) | 0.5 | 9.4 | -3.4 | 9.7 | 0.053 |  |
|  | \|P\| (% to \|E-E\|, right) | 0.0 | 1.0 | -0.4 | 1.1 | 0.102 |  |
| Ls//axial | \|E-E\| (%) | 5.9 | 6.0 | 8.1 | 5.4 | 0.062 |  |
|  | \|M\| (% to \|E-E\|) | -0.7 | 1.3 | -2.2 | 1.4 | 4E-07 | ** |
|  | ∠E-P-M (°, left) | 2.8 | 11.2 | 5.0 | 11.0 | 0.339 |  |
|  | \|P\| (% to \|E-E\|, left) | -0.4 | 1.2 | -1.7 | 1.8 | 2E-04 | ** |
|  | ∠E-P-M (°, right) | -0.1 | 10.5 | 6.0 | 10.6 | 0.006 | * |
|  | \|P\| (% to \|E-E\|, right) | 0.0 | 1.0 | -1.7 | 1.7 | 1E-06 | ** |
| Li//axial | \|E-E\| (%) | 2.9 | 4.9 | 5.9 | 6.2 | 0.018 |  |
|  | \|M\| (% to \|E-E\|) | -0.3 | 0.9 | -2.1 | 1.5 | 4E-09 | ** |
|  | ∠E-P-M (°, left) | -2.6 | 12.9 | 1.6 | 8.9 | 0.047 |  |
|  | \|P\| (% to \|E-E\|, left) | -0.9 | 1.6 | -2.7 | 1.9 | 8E-06 | ** |
|  | ∠E-P-M (°, right) | 0.5 | 11.7 | 3.0 | 9.5 | 0.240 |  |
|  | \|P\| (% to \|E-E\|, right) | -0.7 | 1.8 | -2.7 | 1.6 | 5E-08 | ** |
| Sm//axial | \|E-E\| (%) | 0.7 | 5.1 | 1.4 | 6.9 | 0.618 |  |
|  | \|M\| (% to \|E-E\|) | 0.1 | 5.9 | -0.6 | 1.7 | 0.254 |  |
|  | ∠E-P-M (°, left) | 0.7 | 11.1 | 4.9 | 7.1 | 0.013 |  |
|  | \|P\| (% to \|E-E\|, left) | -0.8 | 9.8 | -2.4 | 3.1 | 0.149 |  |
|  | ∠E-P-M (°, right) | -0.3 | 5.2 | 4.6 | 6.2 | 1E-04 | ** |
|  | \|P\| (% to \|E-E\|, right) | -0.4 | 2.0 | -1.5 | 2.3 | 0.019 |  |

* P < 0.01; ** P < 0.01. For definition of the variables, please see S5 Fig.
