# Supplementary material for: Functional decline in facial expression generation in older women: A cross-sectional study using three-dimensional morphometry
Source: PLoS One. 2019 Jul 10;14(7):e0219451. doi: 10.1371/journal.pone.0219451 (PMC6636602; doi:10.1371/journal.pone.0219451)
Supplement: S9 Table — (DOCX) [file pone.0219451.s011.docx]

***S9 Table.*** *Means and their standard deviations (S.D.) for the 3 variables for the Facial outline contours for each side.*

|  |  | **Rest** | | | | | | **Smile** | | | | | | **P-value (Rest vs. Smile)** | | | |
| --- | --- | --- | --- | --- | --- | --- | --- | --- | --- | --- | --- | --- | --- | --- | --- | --- | --- |
|  | **Variable** | **Older** | | **Younger** | | **P-value** | | **Older** | | **Younger** | | **P-value** | | **Older** | | **Younger** | |
|  |  | **Mean** | **S.D.** | **Mean** | **S.D.** |  |  | **Mean** | **S.D.** | **Mean** | **S.D.** |  |  |  |  |  |  |
| Left | ∠Zy′-Go′ (°) | 80.4 | 2.1 | 78.1 | 2.1 | 5E-07 | ** | 80.9 | 2.4 | 80.6 | 2.4 | 0.398 |  | 0.05 |  | 3E-08 | ** |
|  | ∠Go′-Gn (°) | 25.2 | 4.3 | 27.3 | 4.0 | 0.014 |  | 27.1 | 7.2 | 30.2 | 3.7 | 0.002 | * | 0.17 |  | 3E-10 | ** |
|  | ∠Zy′-Go′-Gn (°) | 124.8 | 5.1 | 129.3 | 4.4 | 9E-06 | ** | 126.2 | 7.0 | 130 | 4.1 | 2E-04 | ** | 0.28 |  | 0.053 |  |
| Right | ∠Zy′-Go′ (°) | 80.6 | 2.4 | 78.8 | 2.2 | 3E-04 | ** | 80.9 | 2.2 | 80.1 | 2.8 | 0.152 |  | 0.14 |  | 8E-10 | ** |
|  | ∠Go′-Gn (°) | 26.1 | 3.1 | 27.4 | 3.3 | 0.067 |  | 26.5 | 3.8 | 30.5 | 3.3 | 1E-07 | ** | 0.52 |  | 1E-12 | ** |
|  | ∠Zy′-Go′-Gn (°) | 125.6 | 3.6 | 128.6 | 3.9 | 3E-04 | ** | 125.4 | 4.8 | 129.9 | 3.4 | 9E-08 | ** | 0.82 |  | 0.001 | * |

For definition of the variables, please see S6 Fig.

***S9 Table Contd.*** *Means and their standard deviations (S.D.) for the 3 variables for the Facial outline contours for each side.*

|  |  | **Smile - Rest** | | | | | |
| --- | --- | --- | --- | --- | --- | --- | --- |
|  | **Variable** | **Older** | | **Younger** | | **P-value** | |
|  |  | **Mean** | **S.D.** | **Mean** | **S.D.** |  |  |
| Left | ∠Zy′-Go′ (°) | 0.5 | 1.3 | 2.0 | 3.3 | 0.016 |  |
|  | ∠Go′-Gn (°) | 1.9 | 7.4 | 2.8 | 4.1 | 0.364 |  |
|  | ∠Zy′-Go′-Gn (°) | 1.4 | 7.0 | 0.8 | 4.3 | 0.592 |  |
| Right | ∠Zy′-Go′ (°) | 0.5 | 1.8 | 1.8 | 2.7 | 0.013 |  |
|  | ∠Go′-Gn (°) | 0.4 | 3.0 | 3.1 | 3.9 | 4E-04 | ** |
|  | ∠Zy′-Go′-Gn (°) | -0.2 | 3.8 | 1.3 | 3.8 | 0.070 |  |

For definition of the variables, please see S6 Fig.
