# Supplementary material for: Functional decline in facial expression generation in older women: A cross-sectional study using three-dimensional morphometry
Source: PLoS One. 2019 Jul 10;14(7):e0219451. doi: 10.1371/journal.pone.0219451 (PMC6636602; doi:10.1371/journal.pone.0219451)
Supplement: S1 Fig — (DOCX) [file pone.0219451.s012.docx]

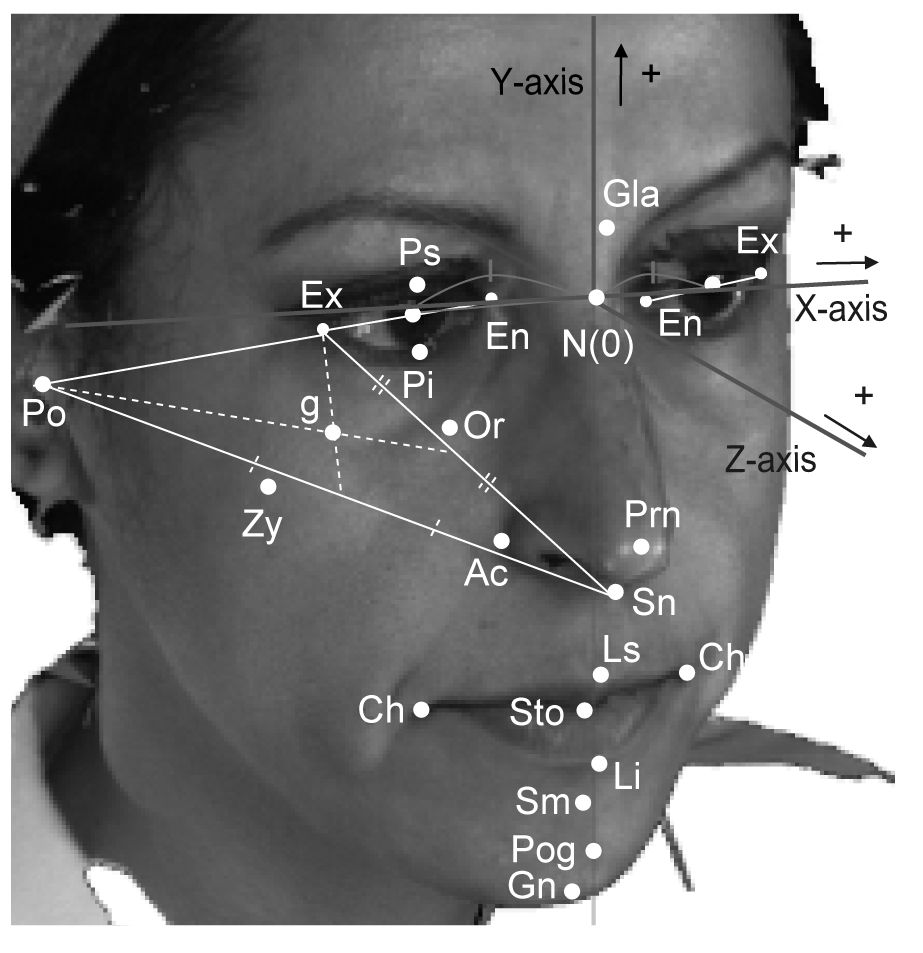


S1 Fig. The coordinate system and landmarks used in the present study. Please see S1 Table for the definitions of the landmarks (cited from Tanikawa et al., 2016 [11]).
