# Supplementary material for: Functional decline in facial expression generation in older women: A cross-sectional study using three-dimensional morphometry
Source: PLoS One. 2019 Jul 10;14(7):e0219451. doi: 10.1371/journal.pone.0219451 (PMC6636602; doi:10.1371/journal.pone.0219451)
Supplement: S2 Fig — (DOCX) [file pone.0219451.s013.docx]

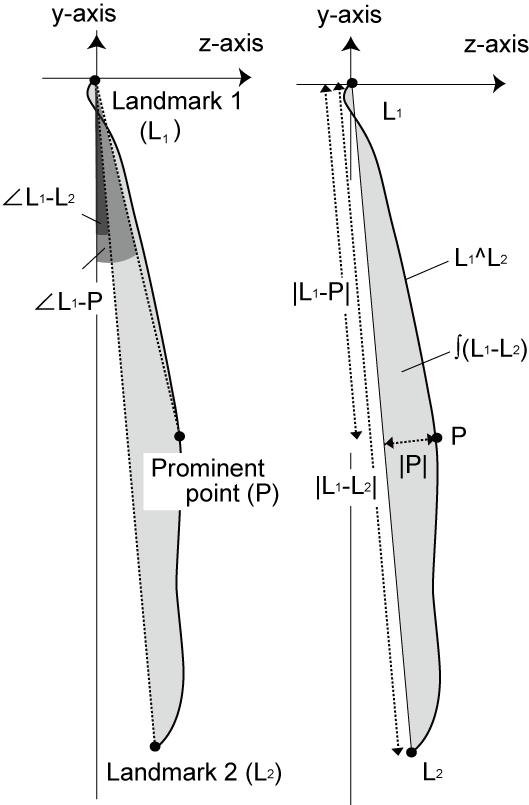


S2 Fig. Schematic diagram illustrating the measurements for the contours Ex-Ac//z, En-Ac//z, Ex-Ch//z, and Ac-Ch//z. The Landmark 1 (L_1_; please see S2 Table) was defined as the origin of the system. The z-axis was defined as the line that passes through the origin and is parallel to ground Z-axis in S1 Fig. The y-axis was defined as the line perpendicular to the z-axis and passing through the origin. ∠L_1_-L_2_ designates the angle formed by the line connecting L_1_ and L_2_ and the y-axis (protrusion of L_2_ relative to L_1_); |L_1_-L_2_|, the distance between L_1_ and L_2_; |P|, the distance between the line L_1_-L_2_ and the most prominent point (P) on the curving line L_1_-L_2_; |L_1_-P|, the distance between L_1_ and the base of the most prominent point along the line L_1_-L_2_; ∫(L_1_-L_2_), the area enclosed by the line L_1_-L_2_ and the contour; ∠L_1_-P, the angle formed by the line connecting L_1_ and the most prominent point and the y-axis; and L_1_^L_2_, the length of the contour along its curvature (cited from Tanikawa et al., 2016 [11]).
