# Supplementary material for: Functional decline in facial expression generation in older women: A cross-sectional study using three-dimensional morphometry
Source: PLoS One. 2019 Jul 10;14(7):e0219451. doi: 10.1371/journal.pone.0219451 (PMC6636602; doi:10.1371/journal.pone.0219451)
Supplement: S3 Fig — (DOCX) [file pone.0219451.s014.docx]

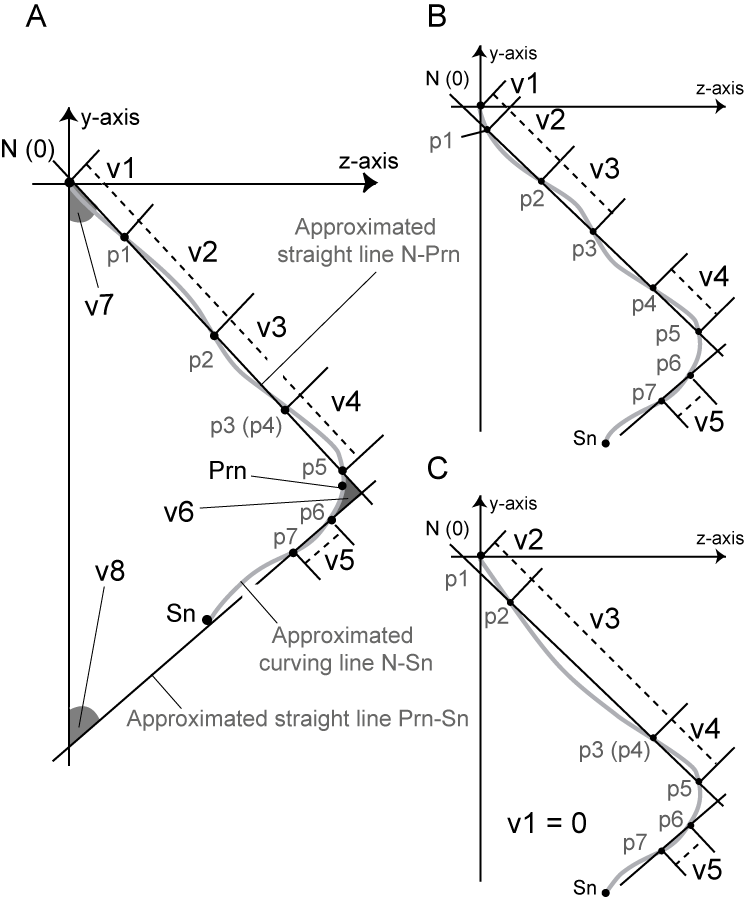


S3 Fig. Schematic diagram illustrating vector elements v1, v2, v3, v4, v5, v6, v7, and v8 of N//sagittal (i.e., the nasal profile). Please see [11] for details. Approximated curving line N-Sn: the 7^th^-order polynomial approximation generated from the contour data between N and Sn. Approximated straight lines N-Prn and Prn-Sn: the first-order polynomial approximations generated from the contour data divided into 2 segments (i.e., N-Prn and Prn-Sn). p1, p2, p3, p4, p5: the intersections between the approximated curving line N-Sn and the approximated straight line N-Prn (the numbers of intersections were 4, 5, and 3 in (A), (B), and (C), respectively). p6, p7: the intersections between the approximated curving line N-Sn and the approximated straight line Prn-Sn. v1 designates the distance between N and p1; v2, the distance between p1 and p2; v3, the distance between p2 and p3; v4, the distance between p4 and p5; v5, the distance between p6 and p7 (the subset [v1, v2, v3, v4] designates the presence or absence, size, and position of the nasal bump; a smaller v1 and greater v3 indicate the absence of the nasal bump); v6, the area enclosed by the approximated straight lines N-Prn and Prn-Sn and the approximated curving line N-Sn (a larger area suggests a more rounded nose tip); v7, the angle formed by the approximated straight line N-Prn and the y-axis (the inclination of the nasal dorsum); and v8, the angle formed by the approximated straight line Prn-Sn and the y-axis (the inclination of the nasal columella). All linear variables were normalized to the difference in the y-coordinate values of N and Sn (cited from Tanikawa et al., 2016 [11]).
