# Supplementary material for: Functional decline in facial expression generation in older women: A cross-sectional study using three-dimensional morphometry
Source: PLoS One. 2019 Jul 10;14(7):e0219451. doi: 10.1371/journal.pone.0219451 (PMC6636602; doi:10.1371/journal.pone.0219451)
Supplement: S4 Fig — (DOCX) [file pone.0219451.s015.docx]

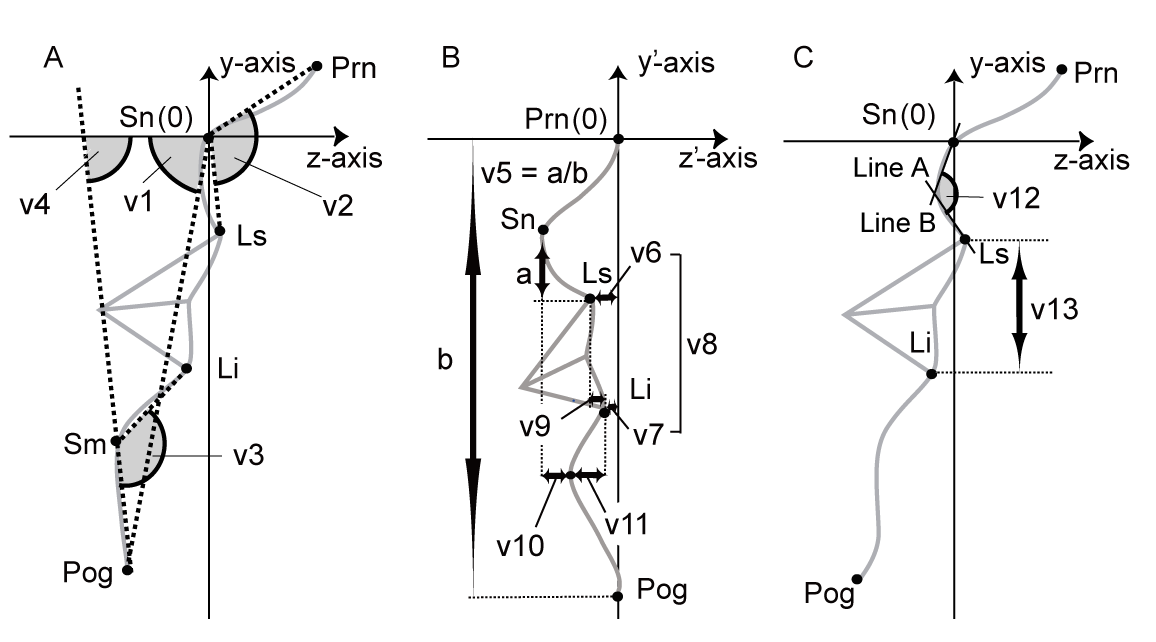


S4 Fig. Measurements for the contour Prn//sagittal (i.e., naso-lip-chin profile [11]). A: Schematic diagram illustrating vector elements v1, v2, v3, and v4. Sn was defined as the origin of the system. The z-axis was defined as the line that passes through the origin and is parallel to ground Z-axis in S1 Fig. The y-axis was defined as the line perpendicular to the z-axis and passing through the origin. The gray line denotes the contour Prn//sagittal. B: Schematic diagram illustrating the definitions of vector elements v5, v6, v7, v8, v9, v10, and v11. Prn was defined as the origin, the y′-axis as the line connecting Prn and Pog, and the z′-axis as the line perpendicular to the y′-axis passing through the origin. C: Schematic diagram illustrating the definitions of vector elements v12 and v13. Sn was defined as the origin, the z-axis as the line through the origin and parallel to ground Z-axis in S1 Fig, and the y-axis as the line through the origin perpendicular to the z-axis. Lines A and B are 1st-order polynomial approximations generated from the extracted contour data (for Line A, the data were extracted from Sn to the midpoint of Sn and Ls; for Line B, the data were extracted from Ls to the midpoint of Sn and Ls). v1 designates the angle formed by the Sn-Pog line and the z-axis (indicating the degree of protrusion of the mandible, with a large value indicating greater protrusion); v2, the angle formed by the Prn-Sn line and Sn-Ls line (indicating the naso-labial angle); v3, the angle formed by the Li-Sm line and Sm- Pog line, the labio-mental angle; v4, the angle formed by the Sm-Pog line and the z-axis (indicating the degree of prominence of the chin, with a small value indicating greater prominence); v5, the value of [the difference between the y′-coordinate values of Sn and Ls]/[the difference between the y′-coordinate values of Prn and Pog] (indicating the vertical length of the subnasal region); v6, the z′ coordinate value of Ls, the sagittal position of the upper lip vermilion, with a positive value indicating protrusion of the upper lip relative to the line connecting Prn and Pog; v7, the z′-coordinate value of Li, with a positive value indicating protrusion of the lower lip relative to the line connecting Prn and Pog; v8, the value of (v6 + v7) (indicating the sagittal position of the upper and lower lip vermilions, with a positive value indicating bilabially protruding lip vermilions); v9, the value of (v7 – v6) (indicating the sagittal relationship between the upper and lower lips, with a positive value indicating a protuberant lower lip relative to its upper counterpart); v10, the difference between the z′-coordinate values of Sn and Sm (indicating the degree of protrusion of the labio-mental sulcus); v11, the difference between the z′-coordinate values of Sm and Li (indicating the depth of the labiomental sulcus); v12, the angle formed by the approximated lines A and B, where Line A was defined as an approximated line between Sn and the midpoint of Sn and Ls and Line B as an approximated line between the midpoint of Sn and Ls (indicating the subnasal (Sn-Ls line) form, with a small value indicating a backward-curving subnasal form); and v13, the difference between the y-coordinate values of Ls and Li (indicating the vertical thickness of the lip vermilions). All linear variables were normalized to the difference between the y-coordinate values of Prn and Pog (cited from Tanikawa et al., 2016 [11]).
