# Supplementary material for: Functional decline in facial expression generation in older women: A cross-sectional study using three-dimensional morphometry
Source: PLoS One. 2019 Jul 10;14(7):e0219451. doi: 10.1371/journal.pone.0219451 (PMC6636602; doi:10.1371/journal.pone.0219451)
Supplement: S5 Fig — (DOCX) [file pone.0219451.s016.docx]

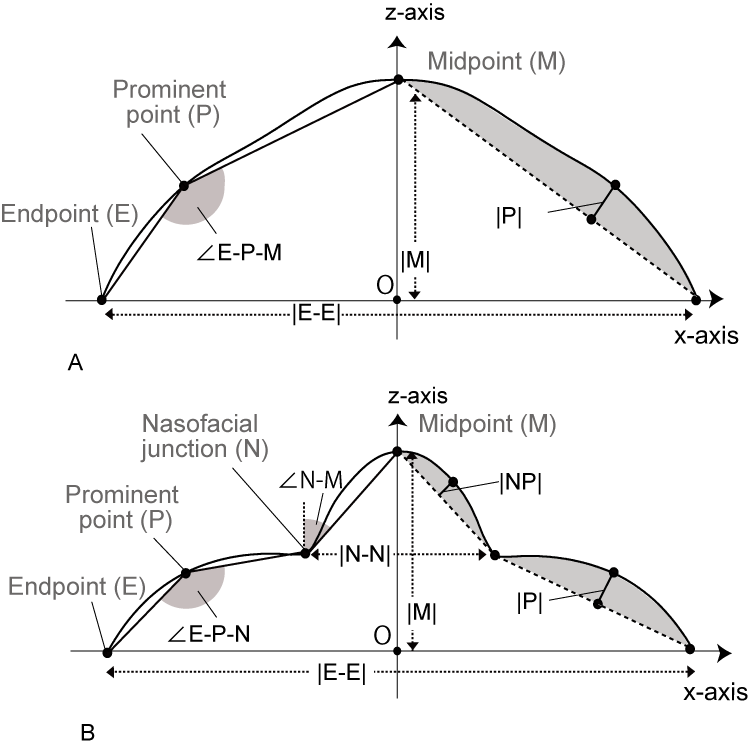


S5 Fig. (A) Schematic diagrams illustrating the measurements of the contours Gla//axial, N//axial, Sn//axial, Ls//axial, Li//axial, and Sm//axial. The endpoints (E) were defined as the 2 endpoints of the surface contour, the midpoint (M) as the mid-sagittal point on the surface contour, and the prominent point (P) as the most prominent point on the skin surface contour between the endpoint and the midpoint. The midpoint of the endpoints was defined as the origin (O) of the system. The x-axis was defined as the line that passes through the origin and is parallel to ground X-axis in S1 Fig. The z-axis was defined as the line perpendicular to the x-axis and passing through the origin. The variable |E-E| designated the distance between left E and right E; |M|, the distance between M and a line connecting the left and right endpoints (indicating the mid-sagittal prominence); ∠E-P-M, the angle formed by E, P, and M (a small value indicates greater cheek prominence); and |P|, the distance between P and the line connecting E and M (indicating a cheek prominence).

(B) Schematic diagram illustrating the measurements of the contours Or//axial and Prn//axial. The endpoints (E) were defined as the 2 endpoints of the surface contour; the midpoint (M) as the mid-sagittal point on the surface contour; the nasofacial junction (N) as the nasofacial junction point on the surface contour; the prominent point (P) as the most prominent point on the surface contour between the endpoint and the nasofacial junction; and the nasal prominent point (NP) as the most prominent point on the surface contour between the nasofacial junction and the midpoint. The midpoint of the endpoints was defined as the origin (O) of the system. The x-axis was defined as the line that passes through the origin and is parallel to ground X-axis in S1 Fig. The z-axis was defined as the line perpendicular to the x-axis and passing through the origin. |N-N| indicates the distance between the 2 nasofacial junction points (indicating the width of the nose at the level of Or or Prn); ∠E-P-N, the angle formed by E, P, and N; |P|, the distance between P and the line connecting E and N (indicating a cheek prominence); ∠N-M, the angle formed by the z-axis and the line connecting N and M (indicating the inclination of the nasal sidewall); and |NP|, the distance between NP and the line connecting N and M (indicating the nasal-dorsum prominence for the Or//axial contour and the nasal-alar prominence for the Prn//axial contour; cited from Tanikawa et al., 2016 [11]).
