# Supplementary material for: Functional decline in facial expression generation in older women: A cross-sectional study using three-dimensional morphometry
Source: PLoS One. 2019 Jul 10;14(7):e0219451. doi: 10.1371/journal.pone.0219451 (PMC6636602; doi:10.1371/journal.pone.0219451)
Supplement: S6 Fig — (DOCX) [file pone.0219451.s017.docx]

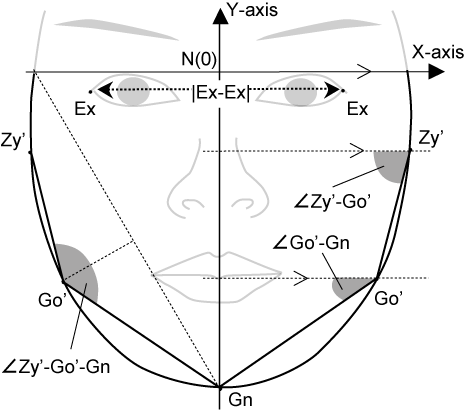


S6 Fig Schematic diagram illustrating the linear and angular measurements of the facial outline. |Ex-Ex| designated the horizontal distance between the exocanthions; ∠Zy′-Go′, the angle formed by the X-axis and the line connecting Zy′ and Go′; ∠Go′-Gn, the angle formed by the X-axis and the line connecting Go′ and Gn; and ∠Zy′-Go′-Gn, the angle formed by the line connecting Zy′ and Go′ and the line connecting Go′ and Gn. Zy′ was mathematically defined as the most lateral point on the facial outline. Go′ was mathematically defined as the most inferior and lateral point on the external angle of the mandible with respect to the line connecting Gn and the point at the intersection of the facial outline with the X-axis (cited from Tanikawa et al., 2016 [11]).
