# Supplementary material for: Functional decline in facial expression generation in older women: A cross-sectional study using three-dimensional morphometry
Source: PLoS One. 2019 Jul 10;14(7):e0219451. doi: 10.1371/journal.pone.0219451 (PMC6636602; doi:10.1371/journal.pone.0219451)
Supplement: S7 Fig — (DOCX) [file pone.0219451.s018.docx]

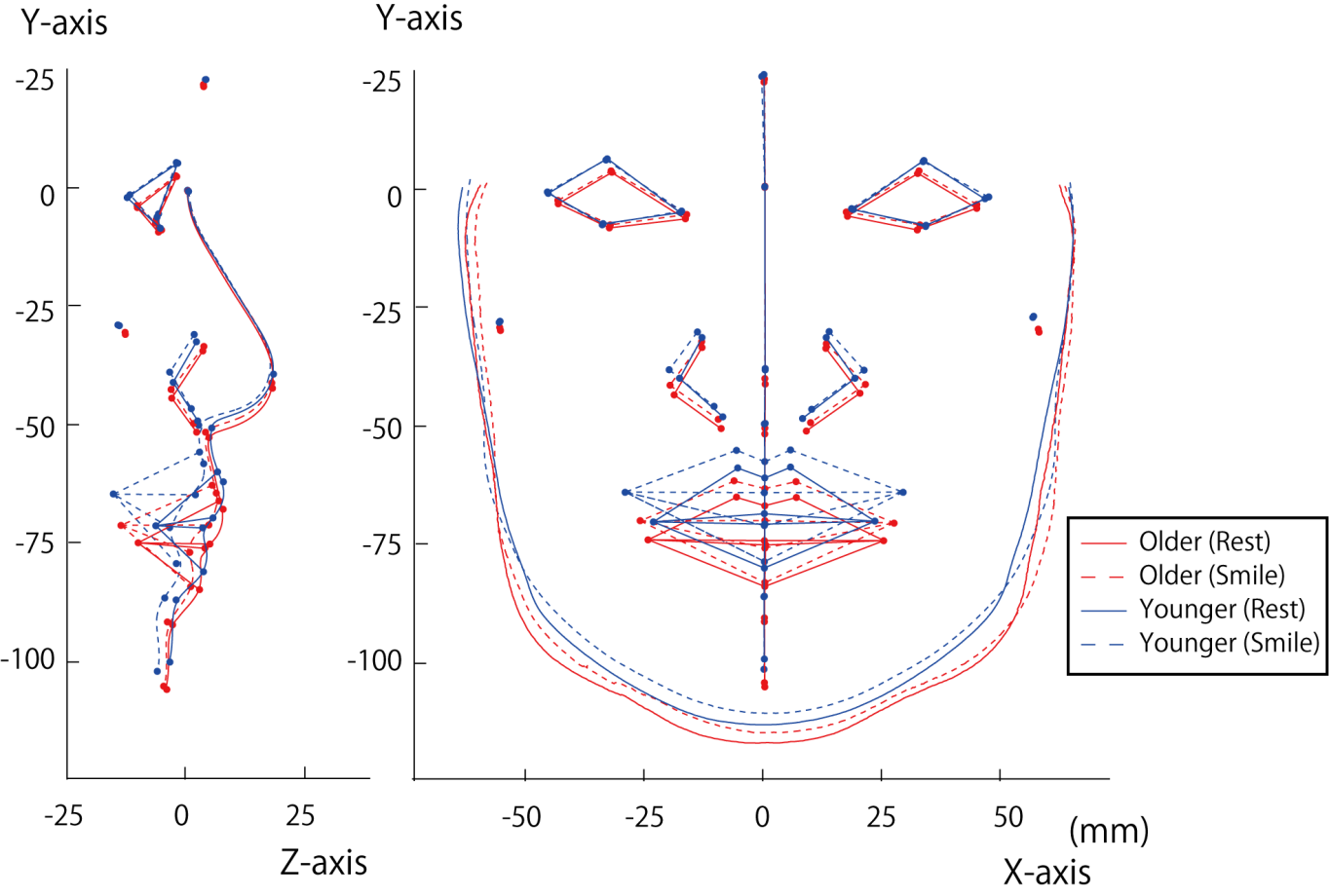


S7 Fig. The mean contours of sagittal sections (N//sagittal and Prn//sagittal [11]), mean facial outlines, and mean coordinates of the landmarks (Gla, Ex, En, Ps, Pi, Prn, Sn, Ls, Sto, Li, Ch, Pog, and Zy; For detail of the landmark definition, please see S1 Table.) for the younger and older groups. Origin, nasion. Red lines indicate the average contour of the older group; blue lines, the younger group; solid lines, rest posture; and dotted lines, smile.
