# Supplementary material for: Functional decline in facial expression generation in older women: A cross-sectional study using three-dimensional morphometry
Source: PLoS One. 2019 Jul 10;14(7):e0219451. doi: 10.1371/journal.pone.0219451 (PMC6636602; doi:10.1371/journal.pone.0219451)
Supplement: S9 Fig — (DOCX) [file pone.0219451.s020.docx]

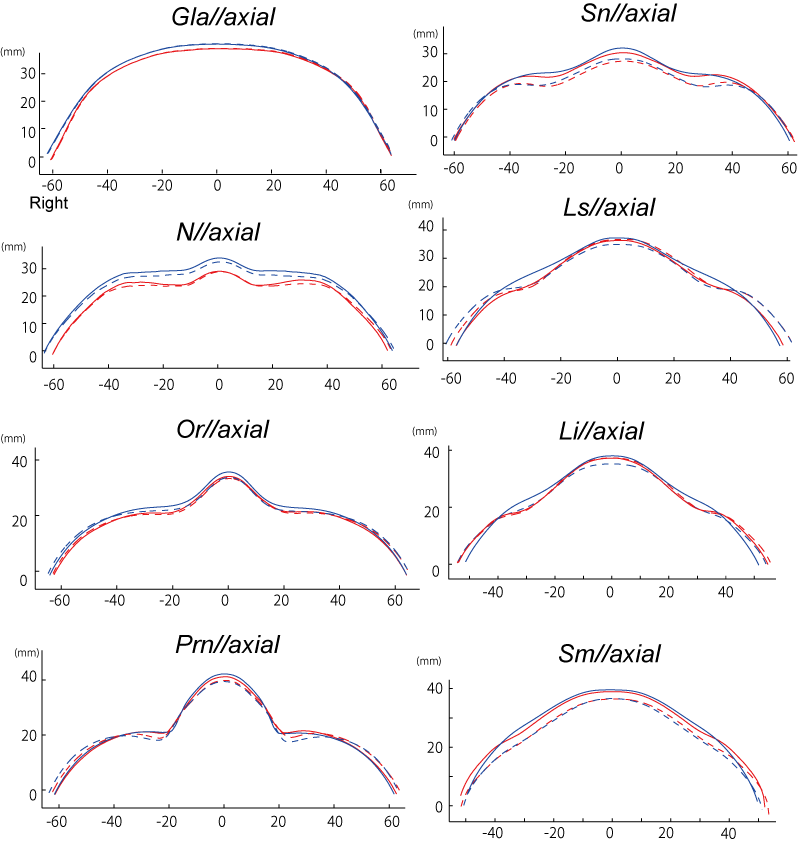


S9 Fig. The mean contours of Gla//axial, N//axial, Or//axial, Prn//axial, Sn//axial, Ls//axial, Li//axial, and Sm//axial in the older group (red lines) and the younger group (blue line). Rest posture was represented as the solid line and the smile posture was represented as the dotted line.
